# Efficacy and safety of ciclosporin versus methotrexate in the treatment of severe atopic dermatitis in children and young people (TREAT): a multicentre parallel group assessor-blinded clinical trial

**DOI:** 10.1093/bjd/ljad281

**Published:** 2023-09-19

**Authors:** Carsten Flohr, Anna Rosala-Hallas, Ashley P Jones, Paula Beattie, Susannah Baron, Fiona Browne, Sara J Brown, Joanna E Gach, Danielle Greenblatt, Ross Hearn, Eva Hilger, Ben Esdaile, Michael J Cork, Emma Howard, Marie-Louise Lovgren, Suzannah August, Farhiya Ashoor, Paula R Williamson, Tess McPherson, Donal O’Kane, Jane Ravenscroft, Lindsay Shaw, Manish D Sinha, Catherine Spowart, Leonie S Taams, Bjorn R Thomas, Mandy Wan, Tracey H Sach, Alan D Irvine, the TREAT Trial Investigators, Alison Layton, Tim Burton, Michael Grainge, Michael Arden-Jones, Saskia King, Michael Perkin, Alain Taieb, Anthony Ormerod, Robert Chalmers, Xinxue Liu, Amina Ahmed, Farhiya Ashoor, Carsten Flohr, Anna Rosala-Hallas, Amy Holton, Hannah Mason, Alan Irvine, Ashley Jones, Tracey Sach, Catherine Spowart, Mandy Wan, Charlotte Walker, Suzannah August, Paula Beattie, Sara Brown, Mike Cork, Ben Esdaile, Carsten Flohr, Joanna Gach, Emma Howard, Alan Irvine, Tess McPherson, Donal O'Kane, Jane Ravenscroft, Lindsay Shaw, Caroline Allen, Susannah Baron, Danielle Greenblatt, Robert Hearn, Susannah Hoey, Rachael Jarret, Catherine Jury, Charlie Mitchell, Ruth Murphy, Graham Ogg, Alice Plant, Louise Newell, Jothsana Srinivasan, Emma Wedgeworth, Fiona Browne

**Affiliations:** Department of Paediatric Dermatology, St John’s Institute of Dermatology, King's College London and Guy's and St Thomas' NHS Foundation Trust, London, UK; Liverpool Clinical Trials Centre, University of Liverpool, Liverpool, UK; Liverpool Clinical Trials Centre, University of Liverpool, Liverpool, UK; Royal Hospital for Children NHS Trust, Glasgow, UK; Department of Paediatric Dermatology, St John’s Institute of Dermatology, King's College London and Guy's and St Thomas' NHS Foundation Trust, London, UK; Paediatric Dermatology, Children’s Health Ireland at Crumlin, Dublin, Ireland; Centre for Genomic and Experimental Medicine, University of Edinburgh, Edinburgh, UK; University Hospitals Coventry and Warwickshire, Coventry, UK; Department of Paediatric Dermatology, St John’s Institute of Dermatology, King's College London and Guy's and St Thomas' NHS Foundation Trust, London, UK; Ninewells Hospital and Medical School, Dundee, UK; Department of Paediatric Dermatology, St John’s Institute of Dermatology, King's College London and Guy's and St Thomas' NHS Foundation Trust, London, UK; Whittington Hospital, Whittington Health NHS Trust, London, UK; Sheffield Children’s NHS Foundation Trust and Sheffield Dermatology Research, Department of Infection, Immunity and Cardiovascular Disease, University of Sheffield, Sheffield, UK; Department of Paediatric Dermatology, St John’s Institute of Dermatology, King's College London and Guy's and St Thomas' NHS Foundation Trust, London, UK; Birmingham Children’s Hospital, Birmingham Women’s and Children’s NHS Foundation Trust, Birmingham, UK; University Hospitals Dorset NHS Foundation Trust, Poole, UK; Liverpool Clinical Trials Centre, University of Liverpool, Liverpool, UK; Liverpool Clinical Trials Centre, University of Liverpool, Liverpool, UK; Oxford University Hospitals NHS Foundation Trust, Oxford, UK; Department of Dermatology, Belfast Health and Social Care Trust, Belfast, UK; Nottingham University Hospitals NHS Trust, Nottingham, UK; Bristol Royal Hospital for Children, Bristol, UK; Kings College London, Department of Paediatric Nephrology, Evelina London Children’s Hospital, Guy’s & St Thomas’s Foundation Hospitals NHS Trust, London; Liverpool Clinical Trials Centre, University of Liverpool, Liverpool, UK; Centre for Inflammation Biology and Cancer Immunology, King's College London, UK; Royal Free Hospital and Blizard Institute, Queen Mary University London, UK; Evelina London Children’s Hospital, Guys’ and St Thomas’ NHS Foundation Trust, London, UK; Institute of Pharmaceutical Science, King’s College London, London, UK; Health Economics Group, Norwich Medical School, University of East Anglia, Norwich, UK; Paediatric Dermatology, Children’s Health Ireland at Crumlin, Dublin, Ireland; Clinical Medicine, Trinity College Dublin, Ireland; National Children’s Research Centre, Crumlin, Dublin, Ireland

## Abstract

**Background:**

Conventional systemic drugs are used to treat children and young people (CYP) with severe atopic dermatitis (AD) worldwide, but no robust randomized controlled trial (RCT) evidence exists regarding their efficacy and safety in this population. While novel therapies have expanded therapeutic options, their high cost means traditional agents remain important, especially in lower-resource settings.

**Objectives:**

To compare the safety and efficacy of ciclosporin (CyA) with methotrexate (MTX) in CYP with severe AD in the TREatment of severe Atopic Eczema Trial (TREAT) trial.

**Methods:**

We conducted a parallel group assessor-blinded RCT in 13 UK and Irish centres. Eligible participants aged 2–16 years and unresponsive to potent topical treatment were randomized to either oral CyA (4 mg kg^–1^ daily) or MTX (0.4 mg kg^–1^ weekly) for 36 weeks and followed-up for 24 weeks. Co-primary outcomes were change from baseline to 12 weeks in Objective Severity Scoring of Atopic Dermatitis (o-SCORAD) and time to first significant flare (relapse) after treatment cessation. Secondary outcomes included change in quality of life (QoL) from baseline to 60 weeks; number of participant-reported flares following treatment cessation; proportion of participants achieving ≥ 50% improvement in Eczema Area and Severity Index (EASI 50) and ≥ 75% improvement in EASI (EASI 75); and stratification of outcomes by filaggrin status.

**Results:**

In total, 103 participants were randomized (May 2016–February 2019): 52 to CyA and 51 to MTX. CyA showed greater improvement in disease severity by 12 weeks [mean difference in o-SCORAD –5.69, 97.5% confidence interval (CI) –10.81 to –0.57 (*P* = 0.01)]. More participants achieved ≥ 50% improvement in o-SCORAD (o-SCORAD 50) at 12 weeks in the CyA arm vs. the MTX arm [odds ratio (OR) 2.60, 95% CI 1.23–5.49; *P* = 0.01]. By 60 weeks MTX was superior (OR 0.33, 95% CI 0.13–0.85; *P* = 0.02), a trend also seen for ≥ 75% improvement in o-SCORAD (o-SCORAD 75), EASI 50 and EASI 75. Participant-reported flares post-treatment were higher in the CyA arm (OR 3.22, 95% CI 0.42–6.01; *P* = 0.02). QoL improved with both treatments and was sustained after treatment cessation. Filaggrin status did not affect outcomes. The frequency of adverse events (AEs) was comparable between both treatments. Five (10%) participants on CyA and seven (14%) on MTX experienced a serious AE.

**Conclusions:**

Both CyA and MTX proved effective in CYP with severe AD over 36 weeks. Participants who received CyA showed a more rapid response to treatment, while MTX induced more sustained disease control after discontinuation.

## Teaser

Ciclosporin and methotrexate are among the most commonly used conventional systemic medications in paediatric patients with moderate-to-severe atopic dermatitis (AD). However, to date, there has been no adequately powered randomized controlled trial (RCT) to assess the comparative efficacy and safety of both therapies in children and young people with AD. Therefore, we conducted a multicentre RCT in the UK and Ireland, which showed that both drugs are safe and effective treatments, with ciclosporin working faster initially but methotrexate showing a more sustained treatment effect, even post-treatment cessation.

## Graphical Abstract

**Graphical Abstract:**
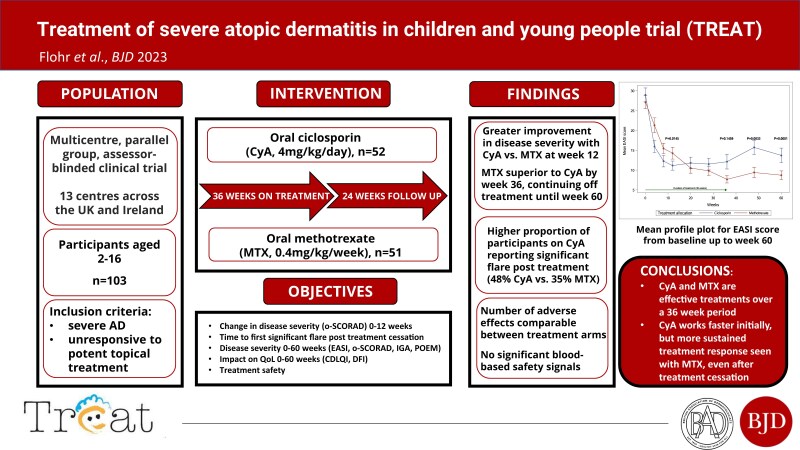

Plain language summary available online

**What is already known about this topic?**

- There is a rapidly evolving novel systemic treatment pipeline for children and young people (CYP) with atopic dermatitis (AD).
- Methotrexate (MTX) and ciclosporin (CyA) are the main conventional systemic treatments used for AD in paediatric patients worldwide.
- Most healthcare settings require patients to travel through a conventional systemic before novel agents are tried; however, there has been no adequately powered randomized controlled trial to establish a gold-standard conventional systemic treatment.

**What does this study add?**

- We show that CyA and MTX are effective treatments over a 36-week period for AD in CYP, with CyA working faster initially and MTX showing a more sustained treatment response, even after treatment cessation.
- We also show that blood monitoring in this age group can be rationalized, as there were few safety signals on safety testing, making the drugs more acceptable to CYP and reducing the overall cost of treatment.

##  

Atopic dermatitis (AD; also called ‘atopic eczema’) is a chronic inflammatory skin disease characterized by intense pruritus, affecting one in five children in the UK and other high-income settings.^1^ Prevalence varies, with a rising incidence in developing countries.^1^ AD is associated with a high-cost burden on patients and families, and on healthcare systems.^2,3^ Children and young people (CYP) with moderate-to-severe AD often suffer significant sleep disturbance and poor mental health, poor attendance at school and social withdrawal. Most cases of AD are adequately controlled with emollients, topical corticosteroids (TCS) or topical calcineurin inhibitors (TCIs).^4^ Treatment options for CYP who do not respond to these topical therapies remain limited.^5^ Around 5% of paediatric patients with AD require systemic drugs to induce and maintain disease control.^6,7^ While a number of monoclonal antibodies and novel small molecules have recently been approved for AD, only dupilumab and upadacitinib are widely approved for CYP older than 12 years, and only dupilumab for those aged ≥ 6 months. Many third-party payers and health technology assessment agencies, such as the UK’s National Institute for Health and Care Excellence, restrict the prescribing of newer drugs to those failing to respond to conventional systemic treatment. With increasing interest in AD globally, cost-effective treatments are in focus for payers. Ciclosporin (CyA) is the most used conventional systemic medication in paediatric patients with moderate-to-severe AD, with methotrexate (MTX) emerging as a potential alternative.^7,8^

A recent network meta-analysis of AD treatments in adults showed that high-dose CyA generally resulted in better improvement than MTX in clinical AD signs, with the therapeutic results comparable to dupilumab up to 16 weeks.^9^ These results correspond to an early systematic review published prior to the introduction of biologic therapies, which recommended CyA over MTX as a treatment for moderate-to-severe AD in adults.^10^ However, there is sparse evidence comparing the efficacy of CyA to MTX in CYP with AD. To date, only one randomized controlled trial (RCT) has compared these two treatments in a paediatric population; it was underpowered (20 patients in each arm) and lacked an intention-to-treat (ITT) analysis.^11^ Participants were given drug doses that were lower than those conventionally used (CyA 2 mg kg–1 daily; MTX 7.5 mg weekly) and were only treated for 12 weeks.^11^

CyA is a calcineurin inhibitor that works by decreasing the production of the inflammatory cytokines associated with AD and inhibiting the activation of T cells by blocking nuclear factor of activated T cell-dependent cytokine production. CyA has a rapid onset of action in AD. There is an increased risk of hypertension and renal toxicity, especially when used long term, and treatment duration in CYP is only recommended up to a maximum of 1 year.^12,13^ In addition, patients on CyA are quick to relapse following treatment cessation.^12^ For a child weighing 38 kg a 36-week treatment course of CyA (4 mg kg^–1^ PO daily) without dose modifications would be £875.70 (or £24.33 per week) in the UK, excluding dispensing costs or National Health Service (NHS) discount.^14^

MTX is a folic acid antagonist that modulates immune system activity and hinders cell division, DNA/RNA synthesis and repair, and protein synthesis. One putative additional mechanism of action is inhibition of the Janus kinase (JAK)/signal transducer and activator of transcription (STAT) pathway.^15,16^ MTX is considered safe for use in CYP,^17,18^ although typical side-effects include nausea, fatigue, deranged liver enzymes and, rarely, bone marrow suppression. MTX has a slower onset of action than CyA. Clinical experience suggests that MTX may have disease-modifying potential, but this has not been formally assessed. The cost of a 36-week treatment course of MTX (0.4 kg^–1^ weekly equating to 15 mg weekly) without dose modifications is a fraction of the cost of the 36-week treatment cost of CyA: £19.65 (or £0.55 per week) for a child weighing 38 kg, excluding the cost of folic acid, dispensing costs or NHS discount.^14^

Here we report the results from the TREatment of severe Atopic Eczema Trial (TREAT), which investigated the efficacy and safety of CyA and MTX in severe AD in CYP.

## Patients and methods

### Study design and participants

TREAT was a multicentre parallel group assessor-blinded superiority RCT (EudraCT 2015-002013-29) conducted at 12 paediatric dermatology departments across the UK and 1 in Ireland. Patients were identified from paediatric dermatology clinics. Eligible patients were between 2 and 16 years old; had severe recalcitrant AD [defined as an Objective Severity Scoring of Atopic Dermatitis (o-SCORAD) ≥ 30]; and an inadequate response to potent topical treatment. AD was diagnosed using the UK refinement of the Hanifin and Rajka criteria.^19^ Patients who had previous exposure to any biologic agents or systemic immunosuppressive therapy were excluded. Any patients who had received systemic corticosteroids within 14 days prior to the screening visit and 28 days of the baseline visit or received phototherapy within 4 weeks prior to the screening visit and 6 weeks of the baseline visit were also excluded, as were patients considered to have a serious underlying medical condition that could have compromised their safety in the study. Full inclusion and exclusion criteria are provided in the published study protocol and in Appendix S2 (see Supporting Information).^6^

The trial was registered in the ISRCTN Registry on 9 March 2016 (ISRCTN1583774).

### Randomization and blinding

Patients were randomly assigned CyA or MTX in a 1 : 1 ratio at the baseline visit using an online randomization program, which concealed allocation and was controlled centrally by the Liverpool Clinical Trials Centre. Owing to the nature of the trial interventions, blinding of the local investigator, research nurse and participants was not possible. The assessor who performed the severity assessments was blinded to the reatment group.

### Procedures

Participants were identified by participating sites. Patients and guardians who expressed an initial interest in the trial were given a Patient Information Sheet and were invited for a screening visit. Each screening visit included a full medical history and concomitant medication review, pregnancy test (where applicable), height, safety blood tests, collection of demographic data and completion of o-SCORAD. Participants suspected of having active tuberculosis underwent a chest radiograph. Those eligible returned for a baseline visit. Baseline assessor-blinded o-SCORAD, Eczema Area and Severity Index (EASI) and validated Investigator’s Global Assessment (v-IGA) assessments were conducted, and Patient-Oriented Eczema Measure (POEM) questionnaires completed. Once all baseline assessments had been performed, participants were randomized to the study drug, which was then dispensed by the local hospital pharmacy.

Participants randomized to the CyA arm (Neoral^®^; Novartis Pharmaceuticals, Basel, Switzerland) were prescribed 4 mg kg^–1^ daily in two divided oral doses for the treatment period of 36 weeks. After 12 weeks, dose increases (up to a maximum of 5 mg kg^–1^ daily) or decreases were allowed, depending on individual treatment response.

Participants randomized to the MTX arm [any brands with UK/European Union (EU) marketing authorization] were prescribed a single oral test dose of 0.1 mg kg^–1^ at week 0 and then 0.4 mg kg^–1^ weekly (maximum dose 25 mg PO weekly) until week 36. Only the MTX 2.5 mg tablets were dispensed. Participants in the MTX arm were also prescribed oral folic acid 1 mg once daily apart from on the day of MTX administration.

Participants randomized to the MTX arm were followed up at week 1, to monitor for potential myelosuppression. All participants were seen at weeks 2, 4, 8, 12, 20, 28, 36, 48 and 60 for efficacy and safety parameters. Quality of life (QoL) questionnaires were collected at weeks 12, 36, 48 and 60. All participants were given diaries to complete weekly over the course of the study.

### Outcomes

The co-primary outcomes were (i) the change in AD severity between baseline and 12 weeks of treatment, using the o-SCORAD severity index; and (ii) time to first significant flare (relapse) after treatment cessation. Significant flare was defined as either having to restart systemic treatment or returning to baseline o-SCORAD, following cessation of trial treatment.

Secondary outcomes were (i) AD severity (EASI, v-IGA, o-SCORAD and POEM); (ii) the number of participant-­reported flares in each study arm following treatment cessation; (iii) the proportion of participants achieving ≥ 50% and ≥ 75% improvement in the EASI (EASI 50 and EASI 75, respectively); IGA and o-SCORAD; (iv) the proportion of participants who withdrew from treatment because of adverse events (AEs); and (v) disease-­specific participant and parental QoL measured with the Children’s Dermatology Life Quality Index (CDLQI)/Infants' Dermatology Quality of Life Index (IDQOL)/Dermatitis Family Impact (DFI) questionnaire. Additional secondary outcomes were number of days on anti-inflammatory treatment during and after treatment reported by participants, and modulation of treatment response by *FLG* loss-of-function mutation inheritance.

All AEs were reported from randomization until 4 weeks after treatment cessation, irrespective of severity or perceived relationship to the study drug. AEs were coded into preferred term and system organ class using the Medical Dictionary for Regulatory Activities (MedDRA; version 19.0).

### Statistical analysis

#### Sample size

For the first co-primary outcome, the study was powered to detect a minimal clinically important difference (MCID) of 8 o-SCORAD points (assuming a SD of 10)^20^ in the change from baseline to 12 weeks for each participant. A sample size of 41 per group, increasing to 49 per group to allow for an estimated 18% loss to follow-up rate, would be required to provide 90% power using a *t*-test with a 0.025 two-sided significance level.

For the second co-primary outcome, the study was powered to detect a difference of 30% (from 86% to 56%) based on the results reported by Harper *et al*.,^12^ which indicated that 86% of participants reflared after the first 3 months of CyA pulse treatment. A sample size of 43 per group, increasing to 51 per group to allow for an estimated 18% loss to follow-up rate, would be required to provide 80% power to detect a reduction in reflare of 30% (from 86% to 56%), using a two-sided test with a 0.025 significance level. A total of 102 participants randomized equally across both arms (*n* = 51) satisfied both outcome calculations.

#### Statistical analysis

All analyses were prespecified in a statistical analysis plan (Appendix S3; see Supporting Information). Evaluation of clinical efficacy followed the ITT principle. We analysed safety in participants who received at least one dose of their allocated trial medication (the safety population). Analyses were performed using SAS (version 9.3 or later; SAS Institute, Cary, NC, USA).

The first co-primary outcome was analysed using an Ancova model and 97.5% confidence intervals (CIs). A sensitivity analysis was conducted that included study site as a random effect in a linear mixed model. The second co-primary outcome assessment was analysed using the Cox proportional hazards model and 97.5% CIs. The assumption of proportional hazards was investigated by the inclusion of an interaction term between time and treatment allocation in the model. A sensitivity analysis was conducted that included only those who completed 36 weeks of treatment. A log-rank χ^2^ test was also performed to compare the difference in number of reflares, as defined in co-primary outcome 2, between treatment groups.

Missing data were monitored throughout the trial with reasons for withdrawals from study captured on the case report form. Withdrawals from the study were censored observations at time of withdrawal within the second co-primary outcome.

Statistical analyses for the secondary outcomes are detailed in Appendix S4.

## Results

**Figure 1:**
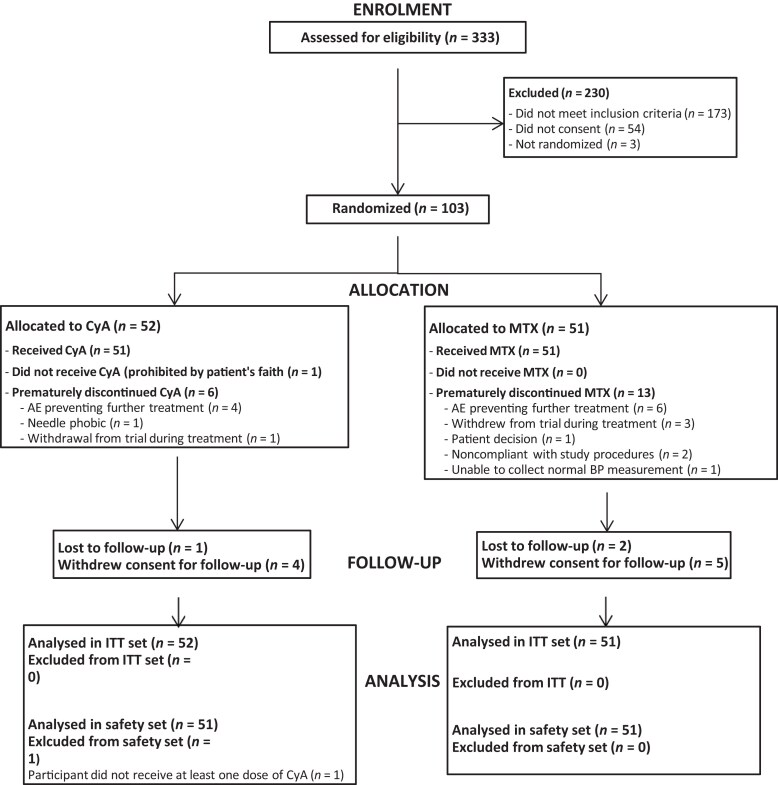
Trial profile. AE, adverse event; BP, blood pressure; CyA, ciclosporin; ITT, intention to treat; MTC, methotrexate.

Between 26 May 2016 and 5 February 2019, 333 participants were screened, of whom 103 were deemed eligible and randomized to CyA (*n* = 52) or MTX (*n* = 51). Recruitment closed once the target was reached. One participant randomized to the CyA group did not receive study treatment for religious reasons (alcohol in the CyA solution; Figure 1). Seven (13%) and 13 (25%) participants prematurely discontinued CyA and MTX treatment, respectively. All 103 participants randomized were included in the ITT analysis. The baseline demographics and clinical characteristics of participants were well balanced across both groups, including the disease severity and QoL scores (Table 1). The final follow-up visit was conducted on 14 May 2020.

**Table 1:** Demographic and baseline characteristics of 103 patients included in the TREatment of severe Atopic Eczema Trial (TREAT) trial

	Ciclosporin (*n* = 52)	Methotrexate (*n* = 51)
Sex		
Female	21 (40)	28 (55)
Male	31 (60)	23 (45)
Ethnicity		
White	31 (60)	30 (59)
Black	7 (13)	4 (8)
Asian	11 (21)	12 (24)
Other	3 (6)	5 (10)
Age (years), mean (SD)	10.34 (4.21)	9.82 (4.01)
BMI (kg m–2)^a^	18.80 (4.16)	19.30 (4.15)
o-SCORAD, mean (SD)	48.34 (11.35)	45.25 (9.60)
EASI, mean (SD)	28.97 (12.53)	27.12 (11.62)
v-IGA		
Mild	0 (0)	1 (2)
Moderate	16 (31)	18 (35)
Severe	31 (60)	29 (57)
Very severe	5 (10)	3 (6)
POEM, mean (SD)^b^	20.40 (5.26)	20.84 (5.47)
DFI, mean (SD)^a^	15.24 (7.89)	15.59 (7.67)
CDLQI, mean (SD)^c^	14.67 (6.96)	15.26 (6.57)

Data are presented as *n* (%) unless otherwise stated. BMI, body mass index; CDLQI, Children’s Dermatology Life Quality Index; DFI, Dermatitis Family Impact; EASI, Eczema Area and Severity Index; POEM, Patient Oriented Eczema Measure; o-SCORAD, Objective Severity Scoring of Atopic Dermatitis; v-IGA, validated Investigator’s Global Assessment. ^a^One missing ciclosporin (CyA) measurement; ^b^two missing CyA and two missing methotrexate (MTX) assessments; and ^c^three excluded assessments and one missing CyA assessment, and four missing MTX assessments.

There was a statistically significant improvement in o-SCORAD in the CyA group vs. the MTX group at week 12, with a mean difference in change between baseline and 12 weeks of –5.69 (97.5% CI –10.81 to –0.57; *P* = 0.01). Forty-three participants experienced a significant flare (relapse) after treatment cessation: 25 (48%) in the CyA group and 18 (35%) in the MTX group. Six participants in the CyA group had a significant flare after stopping treatment (four participants returned to baseline o-SCORAD or worse and two restarted a systemic) and one participant in the MTX group had a significant flare after restarting a systemic treatment. There was no statistically significant difference between treatment groups with regard to the second co-primary outcome: time to first significant flare after treatment cessation [log-rank test *P* = 0.15; hazard ratio 1.55 (97.5% CI 0.77–3.10), *P* = 0.16] (Figure S1; see Supporting Information). Sensitivity analyses yielded comparable results (Tables S1–S3; see Supporting Information).

**Figure 2:**
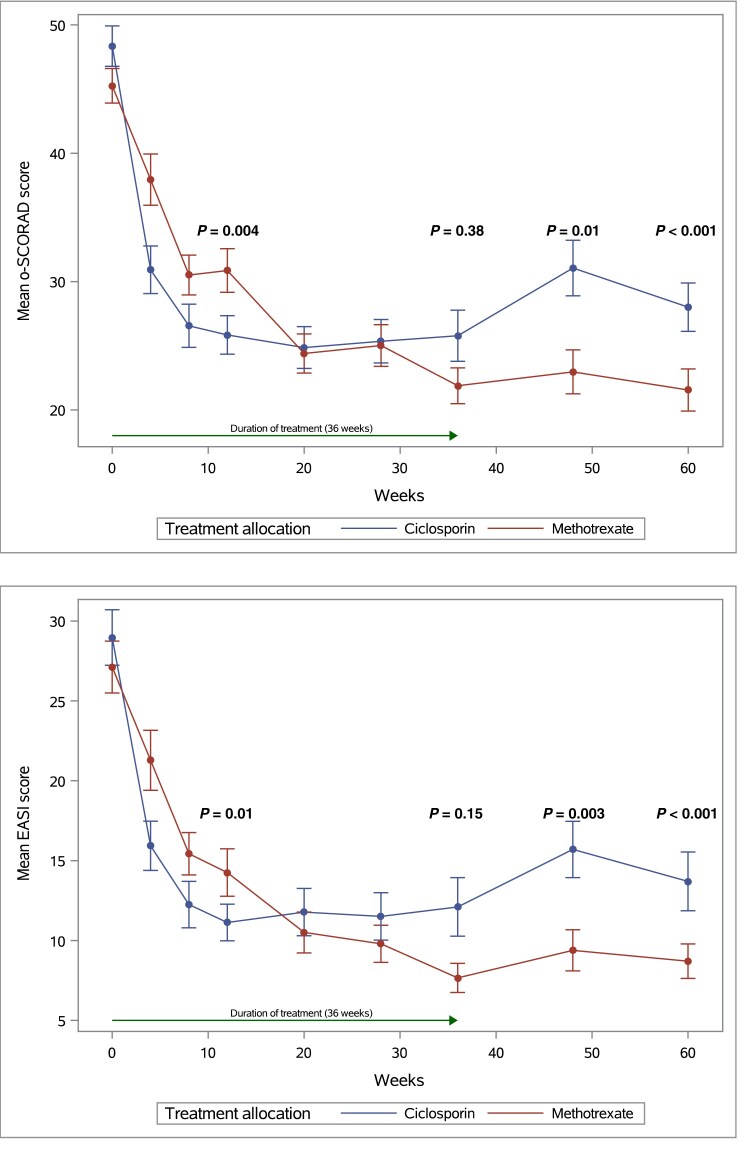
Mean profile plots for Objective Severity Scoring of Atopic Dermatitis (o-SCORAD) and Eczema Area and Severity Index (EASI) from baseline up to week 60. Point estimates at each timepoint are means with standard error bars; *P*-values are taken from linear mixed-model estimates.

Regarding the secondary outcomes, mean profile plots showed greater improvement in disease severity scores in the CyA group at 12 weeks, no difference at 36 weeks and in favour of MTX at 48 (12 weeks post-treatment) and 60 weeks (24 weeks post-treatment) [Figure 2; Figures S2, S3 (see Supporting Information)]. The linear mixed models confirmed these findings [Table 2; Table S4 (see Supporting Information)].

**Table 2:** Estimates from the random-effects models for the longitudinal secondary outcomes o-SCORAD, Objective Severity Scoring of Atopic Dermatitis (o-SCORAD) and Eczema Area and Severity Index (EASI) at each timepoint in the TREatment of severe Atopic Eczema Trial (TREAT) trial

	Time (weeks)	Ciclosporin		Methotrexate		Estimated difference in means (SE)	95% confidence interval	*P*-value
		*n*	Estimated mean (SE) score	*n*	Estimated mean (SE) score			
o-SCORAD	12	52	26.53 (1.13)	51	31.32 (1.15)	–4.80 (1.62)	–8.00, –1.59	0.004
	36	48	27.09 (1.10)	46	25.64 (1.11)	1.44 (1.57)	–1.67, 4.56	0.36
	48	47	27.37 (1.21)	45	22.80 (1.23)	4.56 (1.74)	1.14–7.99	0.009
	60	46	27.64 (1.39)	44	19.96 (1.41)	7.68 (1.99)	3.77–11.60	< 0.001
EASI	12	52	12.36 (0.86)	51	15.49 (0.87)	–3.13 (1.22)	–5.55, –0.72	0.01
	36	48	12.81 (0.82)	46	11.19 (0.84)	1.61 (1.18)	–0.72, 3.94	0.17
	48	47	13.03 (0.93)	45	9.04 (0.94)	3.99 (1.33)	1.37–6.60	0.003
	60	46	13.25 (1.09)	44	6.89 (1.10)	6.36 (1.55)	3.31–9.41	< 0.001

The proportion of participants achieving ≥ 50% improvement in o-SCORAD (o-SCORAD 50) was significant at 12 weeks in favour of the CyA group (OR 2.60, 95% CI 1.23–5.49; *P* = 0.01). There were no significant differences between treatment groups at 36 or 48 weeks, but by 60 weeks the proportion of participants achieving o-SCORAD-50 was in favour of the MTX group (OR 0.33, 95% CI 0.13–0.85; *P* = 0.02) (Table S5; see Supporting Information).

Comparison of the mean number of participant-reported flares following trial treatment cessation showed a significant difference between the two groups (3.22, 95% CI 0.42–6.01; *P* = 0.02), with a higher number in the CyA group (9.41) vs. the MTX group (6.19).

There was no evidence that *FLG* mutation status modified treatment effect at 12, 36 or 60 weeks (Table S6; see Supporting Information).

Post-hoc analysis indicated that the proportions of participants achieving EASI 50, EASI 75 and EASI 90 at week 12 in the CyA group was significantly higher compared with those in the MTX group, although by week 60 this effect had reversed (Table S7; see Supporting Information). The proportion of participants achieving v-IGA 0 or 1 was higher in the CyA group at week 12 (*n* = 6/52; 11%) than in the MTX group (*n* = 1/51; 2.0%), similar at week 36 and higher in the MTX group at weeks 48 and 60 (Table S8; see Supporting Information).

In both treatment groups, QoL (estimated by CDLQI, DFI and IDQOL) improved postbaseline to a level of the MCID for these scores (Figures S4–S6; see Supporting Information). There were no significant differences in these scores between the treatment groups at any time point (Tables S9, S10; see Supporting Information).

Overall, participants in the CyA group reported a higher number of days on topical anti-inflammatory treatments than those in the MTX group over the entire course of the trial (Table S11; see Supporting Information). The mean (SD) total number of days on TCS was 94.50 (37.36) in the CyA group vs. 78.72 (56.46) in the MTX group. The mean (SD) total number of days on TCIs was 51.16 (56.60) in the CyA group vs. 26.09 (35.46) in the MTX group. A higher number of mean (SD) total days on emollients [159.52 (67.86)] was reported in the MTX group vs. the CyA group [142.00 (35.25)].

### Treatment safety

Safety data were collected for 102 participants (51 in the CyA group and 51 in the MTX group) who had at least one dose of trial treatment. Overall, 776 nonserious AEs were reported over the course of the study. In total, 369 AEs were experienced by 48 (94.1%) participants in the CyA cohort and 407 by 47 (92%) participants in the MTX arm. The five most frequently reported AEs in the CyA group in descending order were AD flares (43%), headache (27%), abnormal (decrease of > 20% from baseline) estimated glomerular filtration rate (GFR; 27.5%), upper abdominal pain (18%) and vomiting (18%). In the MTX group, the five most frequently reported AEs (in descending order) were nausea (43%), AD flares (29%), fatigue (23%), headache (22%) and vomiting (18%) [Table 3; Table S12 (see Supporting Information)]. All GFRs with a > 20% drop from baseline corrected when participants were encouraged to hydrate prior to repeat testing.

**Table 3:** Most common nonserious adverse events (AEs) occurring in at least 10% of participants in the TREatment of severe Atopic Eczema Trial (TREAT) trial

	Ciclosporin (*n* = 51)		Methotrexate (*n* = 51)		Total (*n* = 102)	
	Events	Participants	Events	Participants	Events	Participants
Any nonserious AE	369	48 (94)	407	47 (92)	776	95 (93.1)
Most common nonserious AEs						
*Skin and subcutaneous tissue disorders*						
Eczema	45	22 (43)	19	15 (29)	64	37 (36.3)
*Nervous system disorders*						
Headache	24	14 (27)	27	11 (22)	51	25 (24.5)
*Gastrointestinal disorders*						
Abdominal pain upper	18	9 (18)	11	3 (6)	29	12 (11.8)
Vomiting	13	9 (18)	11	9 (18)	24	18 (17.6)
Nausea	12	9 (18)	35	22 (43)	47	31 (30.4)
Abdominal pain	10	7 (14)	14	2 (4)	24	9 (8.8)
Diarrhoea	10	8 (16)	8	7 (14)	18	15 (14.7)
Mouth ulceration	0	0 (0)	12	6 (12)	12	6 (5.9)
Investigations						
*Glomerular filtration rate abnormal*	17	14 (27)	14	8 (15.7)	31	22 (21.6)
Infections and infestations						
*Nasopharyngitis*	8	7 (14)	9	9 (18)	17	16 (15.7)
*Eczema infected*	8	6 (12)	8	6 (12)	16	12 (11.8)
General disorders and administration site conditions						
*Fatigue*	4	3 (6)	35	12 (23)	39	15 (14.7)
Metabolism and nutrition disorders						
*Decreased appetite*	4	3 (6)	11	8 (16)	15	11 (10.8)

Data are presented as *n* (%).

Serious AEs (SAEs) were experienced by five participants in the CyA group (10%) and seven participants in the MTX group (14%; Table 4). Of the five SAEs reported in the CyA group, two were deemed by the investigator to be either possibly or probably related to study treatment. One participant developed a bacterial lower respiratory tract infection of moderate severity, and one developed eczema herpeticum of moderate severity, requiring hospital admission. The latter participant subsequently withdrew from the study. Of the seven SAEs reported in the MTX group, two were deemed by the investigator to be either possibly or probably related to study treatment. One participant developed herpes zoster shingles infection of mild severity, and one developed eczema herpeticum classified as severe. Both required hospital admission and both were subsequently withdrawn from study treatment. Overall, 10 participants withdrew from study medication due to an adverse event: 8% in the CyA group and 12% in the MTX group (OR 0.63; *P* = 0.53) (Figure 1). Two participants in the MTX arm discontinued treatment because of nausea. No blood abnormalities were recorded as SAEs and, even among nonserious AEs (excluding abnormal estimated GFR), these were rare (Table S12).

**Table 4:** Serious adverse events in the TREatment of severe Atopic Eczema Trial (TREAT) trial

	Ciclosporin (*n* = 51)		Methotrexate (*n* = 51)		Total (*n* = 102)	
	Events	Participants	Events	Participants	Events	Participants
Skin and subcutaneous tissue disorders	1	1 (2)	0	0 (0)	1	1 (1.0)
Infections and infestations	3	3 (6)	4	4 (8)	7	7 (6.9)
Ear and labyrinth disorders	1	1 (2)	1	1 (2)	2	2 (2.0)
Respiratory, thoracic and mediastinal disorders	0	0 (0)	2	2 (4)	2	2 (2.0)

Data are presented as *n* (%).

## Discussion

We conducted a multicentre assessor-blinded RCT comparing CyA and MTX in paediatric patients with AD recalcitrant to potent topical therapy. Those treated with CyA had a greater improvement in o-SCORAD between baseline and 12 weeks than those given MTX. By 36 weeks there was no difference between treatment groups, measured by o-SCORAD. After treatment discontinuation (weeks 48 and 60), the o-SCORAD of participants in the MTX group was significantly lower compared with those treated with CyA. These results were mirrored by the mean reduction in EASI, o-SCORAD and POEM scores, as well as the categorical severity measure scores (EASI and o-SCORAD 50, 75 and 90, and IGA 0/1) across the study timepoints. There was no difference between treatment groups in the number of participants needing to restart systemic therapy or returning to baseline o-SCORAD following treatment cessation – a very high bar as a definition of significant disease reflare (relapse). However, there was a higher number of participant-reported flares in the CyA vs. the MTX group. There were no statistically significant differences noted in CDLQI/IDQoL or DFI scores across treatment groups, although both showed a clear decrease in scores from baseline to week 12 above the MCID; this effect was largely sustained during follow-up off therapy.

The number of participants in the CyA group using either TCS or TCI in the 24 weeks post-treatment discontinuation was consistently higher than in the MTX group. Although marginally fewer participants in the CyA group were diagnosed with a skin infection or were prescribed antibiotics post-treatment discontinuation vs. the MTX group, the mean number of participant-reported flares post-treatment cessation was higher in the CyA group than in the MTX group. Taken together, this suggests that flares were more common in the CyA group, once treatment was discontinued.

The incidence of SAEs was relatively low in both treatment groups but slightly higher than in two other monotherapy novel systemic trials recently conducted in adolescents, one with subcutaneous dupilumab (interleukin-4 receptor α-­antagonist) and another with oral abrocitinib (JAK1 inhibitor).^21,22^ The number of participants who discontinued treatment due to treatment-related AEs was low in both groups in the TREAT trial, as was the incidence of serious and severe infections. Only two participants in the MTX arm discontinued treatment due to nausea. The majority of AEs were mild and there were no significant abnormalities on blood-safety testing.

Both CyA and MTX resulted in similar disease improvement above the MCID for all severity scores after week 36, indicating that both are effective options for CYP with severe AD. Owing to its slightly faster action, CyA may be a better choice where rapid disease control would benefit the participant. However, participants continued to be assessed over 24 weeks off treatment, and these data showed better disease control in the MTX vs. CyA groups, in keeping with a degree of disease modification by MTX – an outcome our trial was designed to evaluate. Looking at the treatment response curves at 36 weeks, MTX appeared to not have reached its full therapeutic potential, and the trial could have benefitted from an even longer phase on treatment. A further shortcoming of the trial is the absence of patient-reported itch parameters, which at the time of trial conception were not routinely collected in AD clinical trials.

EASI 75 at week 12 was higher in the CyA arm (44%) than in the MTX arm (20%) (Table S7). EASI 75 results from three other monotherapy novel systemic trials conducted in adolescents showed that 51% achieved EASI 75 at week 16 using subcutaneous dupilumab, 61% at week 12 with oral abrocitinib and 33% at week 16 with subcutaneous tralokinumab.^21–23^ Both CyA and MTX were more effective by week 12 than oral baricitinib, as measured by EASI 75.^24^ The EASI 75 response was maintained until the end of treatment at week 36 for CyA (42%), with an improved EASI 75 response in the MTX arm (46%), suggesting equal if not greater efficacy than CyA over a longer treatment period.

In the MTX group the mean post-treatment EASI score was 8 (Table 2), aligning with a proposed therapeutic target for systemic therapy in AD.^25^ The mechanism of action of MTX in immune-mediated inflammatory dieases is incompletely understood. One explanation is that MTX reduces the expression of T helper (Th)2 and Th22 cytokines, possibly through JAK/STAT inhibition,^15,16^ which have been implicated in a decrease of filaggrin production. Natural moisturizing factor (NMF) is significantly reduced in severe AD, independent of *FLG* loss-of-function status.^26,27^ In this trial we found that MTX leads to prolonged disease control, even after treatment cessation. Further investigations as part of the TREAT trial are underway to understand the potential role of NMF in the mechanism of action of MTX.

Neither CyA nor MTX is licensed for the treatment of AD in CYP. CyA has a treatment label for AD in adults in the UK/EU and was the most widely prescibed conventional systemic in CYP in Europe and North America, despite its significantly higher cost.^7,8,28^ Higher drug costs restrict the use of CyA in middle- and lower-income settings, where MTX is the only affordable systemic AD medication. Here, we present a robust evidence base for the efficacy of MTX. Furthermore, this study fills a significant research gap comparing the efficacy of two frequently prescribed treatments in CYP in AD. Future research should take advantage of AD registers, such as the UK–Irish Atopic Eczema Systemic Therapy Register (A-STAR; www.astar-register.org), which provide prospective ‘real-world’ cohorts, from which further comparative analyses can be done.

In conclusion, the TREAT trial demonstrated that both CyA and MTX are effective, well-tolerated treatments for CYP with severe AD. CyA acts more quickly, while MTX induces better disease control after treatment discontinuation. Where first-line novel systemic biologics and small-molecule prescribing is restricted by regulatory and/or funding bodies, MTX provides an efficacious and low-cost alternative to CyA. This is particularly relevant for healthcare settings with limited financial resources. The optimum duration for MTX therapy and the possibility of MTX inducing disease modification merit additional investigation.

## Supplementary Material

ljad281_Supplementary_Data

## Data Availability

Data collected for the study, including deidentified individual participant data and a data dictionary defining each field in the set, can be made available to researchers who provide a methodologically sound proposal to the corresponding author with a signed data-access agreement. The study protocol, statistical analysis plan and health economics analysis plan are available on the trial website (https://www.nottingham.ac.uk/research/groups/cebd/projects/1eczema/beep-maintrial.aspx) and the National Institute for Health Research journals library (https://www.journalslibrary.nihr.ac.uk/programmes/hta/126712/#/). All other related documents are available upon request at any point.
