# Correction to: Efficacy and safety of ciclosporin versus methotrexate in the treatment of severe atopic dermatitis in children and young people (TREAT): a multicentre parallel group assessor-blinded clinical trial

**DOI:** 10.1093/bjd/ljad442

**Published:** 2023-11-16

This is a correction to: Carsten Flohr, Anna Rosala-Hallas, Ashley P Jones, Paula Beattie, Susannah Baron, Fiona Browne, Sara J Brown, Joanna E Gach, Danielle Greenblatt, Ross Hearn, Eva Hilger, Ben Esdaile, Michael J Cork, Emma Howard, Marie-Louise Lovgren, Suzannah August, Farhiya Ashoor, Paula R Williamson, Tess McPherson, Donal O’Kane, Jane Ravenscroft, Lindsay Shaw, Manish D Sinha, Catherine Spowart, Leonie S Taams, Bjorn R Thomas, Mandy Wan, Tracey H Sach, Alan D Irvine, the TREAT Trial Investigators, Efficacy and safety of ciclosporin versus methotrexate in the treatment of severe atopic dermatitis in children and young people (TREAT): a multicentre parallel group assessor-blinded clinical trial, *British Journal of Dermatology*, Volume 189, Issue 6, December 2023, Pages 674–684, https://doi.org/10.1093/bjd/ljad281

In the originally published version of this manuscript, Manish D Sinha's affiliation was incorrectly given as ‘Department of Paediatric Nephrology, Evelina London Children's Hospital, King's College London, London, UK’. This has been corrected.

Additionally, the second sentence of the Acknowledgements section originally read: ‘We are grateful to Edel O'Toole and her team (Royal London Hospital and Blizard Institute, Queen Mary University of London) for their assistance in testing participants’ blood samples for *FLG* mutations.’ This has been corrected to: ‘We are grateful to Edel O'Toole and her team (Royal London Hospital and Genome Centre, Blizard Institute, Queen Mary University of London) for their assistance in testing participants’ blood samples for mutations in the *FLG* gene.’

