# Supplementary material for: Efficacy and safety of ciclosporin versus methotrexate in the treatment of severe atopic dermatitis in children and young people (TREAT): a multicentre parallel group assessor-blinded clinical trial
Source: Br J Dermatol. 2023 Sep 19;189(6):674–84. doi: 10.1093/bjd/ljad281 (PMC13077216; doi:10.1093/bjd/ljad281)
Supplement: ljad281_Supplementary_Data [file ljad281_supplementary_data.zip › 23_534 JC.pptx]

## Slide 1
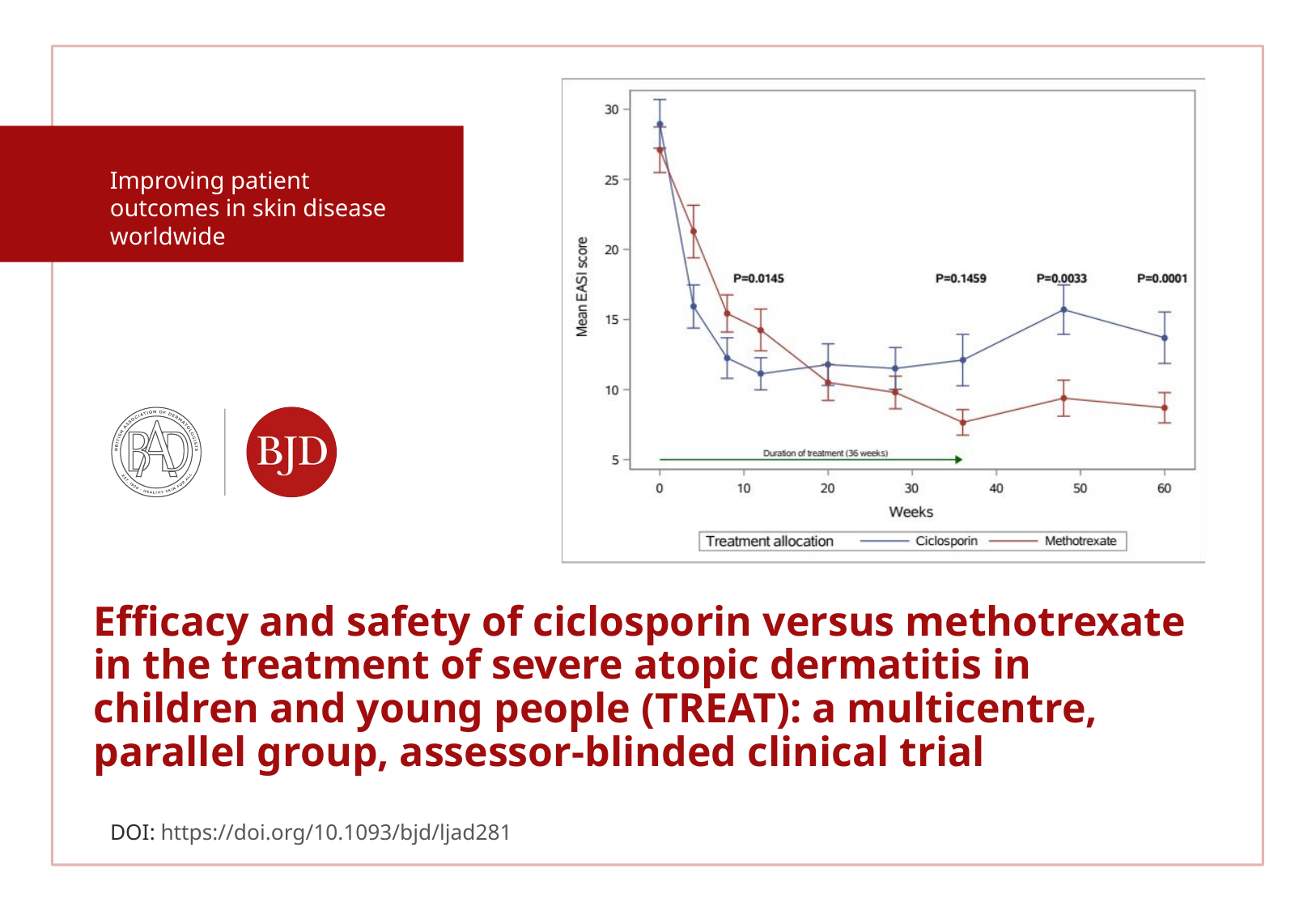

# Efficacy and safety of ciclosporin versus methotrexate in the treatment of severe atopic dermatitis in children and young people (TREAT): a multicentre, parallel group, assessor-blinded clinical trial
DOI: https://doi.org/10.1093/bjd/ljad281

## Slide 2
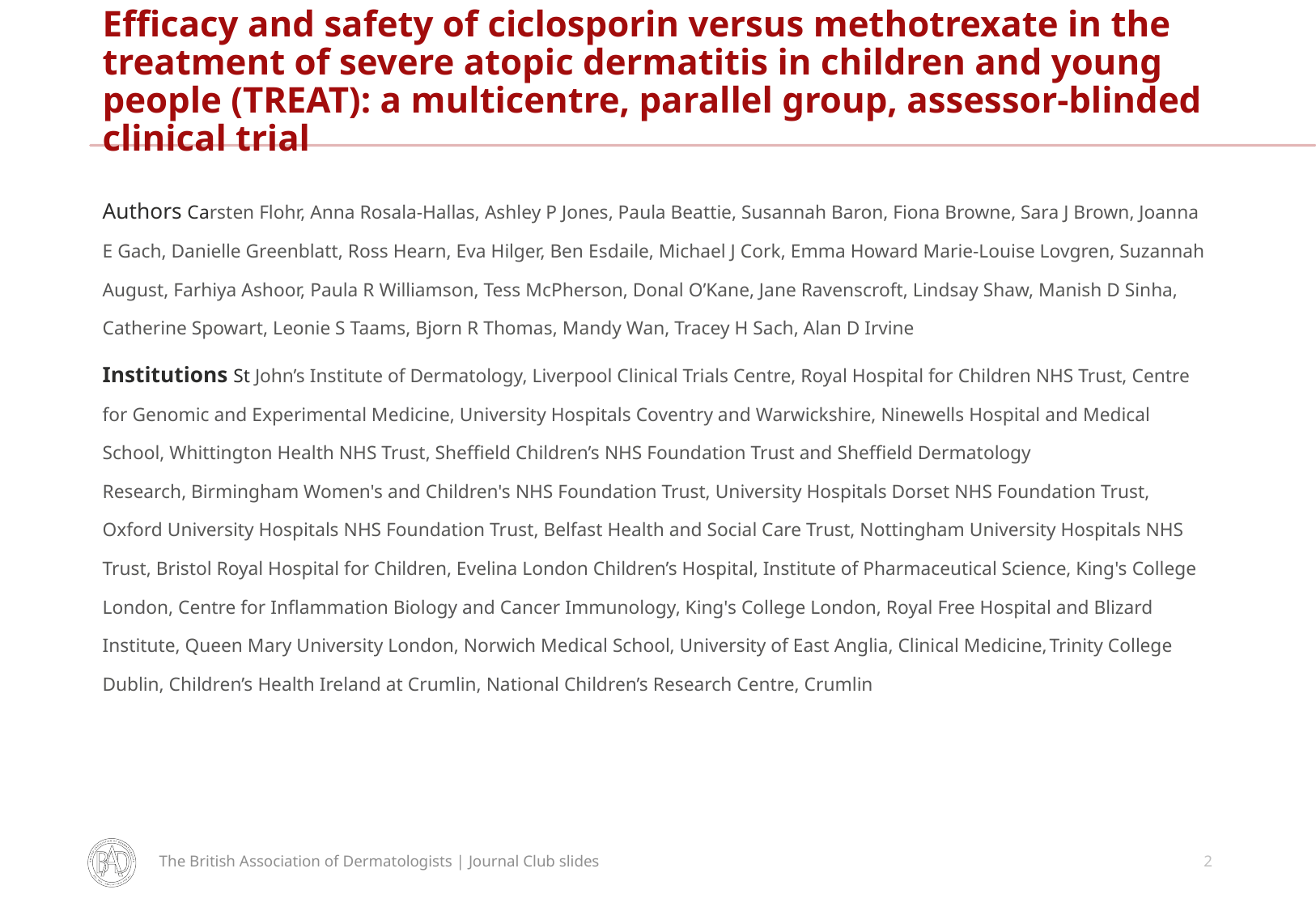

# Efficacy and safety of ciclosporin versus methotrexate in the treatment of severe atopic dermatitis in children and young people (TREAT): a multicentre, parallel group, assessor-blinded clinical trial
Authors Carsten Flohr, Anna Rosala-Hallas, Ashley P Jones, Paula Beattie, Susannah Baron, Fiona Browne, Sara J Brown, Joanna E Gach, Danielle Greenblatt, Ross Hearn, Eva Hilger, Ben Esdaile, Michael J Cork, Emma Howard Marie-Louise Lovgren, Suzannah August, Farhiya Ashoor, Paula R Williamson, Tess McPherson, Donal O’Kane, Jane Ravenscroft, Lindsay Shaw, Manish D Sinha, Catherine Spowart, Leonie S Taams, Bjorn R Thomas, Mandy Wan, Tracey H Sach, Alan D Irvine
Institutions St John’s Institute of Dermatology, Liverpool Clinical Trials Centre, Royal Hospital for Children NHS Trust, Centre for Genomic and Experimental Medicine, University Hospitals Coventry and Warwickshire, Ninewells Hospital and Medical School, Whittington Health NHS Trust, Sheffield Children’s NHS Foundation Trust and Sheffield Dermatology Research, Birmingham Women's and Children's NHS Foundation Trust, University Hospitals Dorset NHS Foundation Trust, Oxford University Hospitals NHS Foundation Trust, Belfast Health and Social Care Trust, Nottingham University Hospitals NHS Trust, Bristol Royal Hospital for Children, Evelina London Children’s Hospital, Institute of Pharmaceutical Science, King's College London, Centre for Inflammation Biology and Cancer Immunology, King's College London, Royal Free Hospital and Blizard Institute, Queen Mary University London, Norwich Medical School, University of East Anglia, Clinical Medicine, Trinity College Dublin, Children’s Health Ireland at Crumlin, National Children’s Research Centre, Crumlin
The British Association of Dermatologists | Journal Club slides
2

## Slide 3
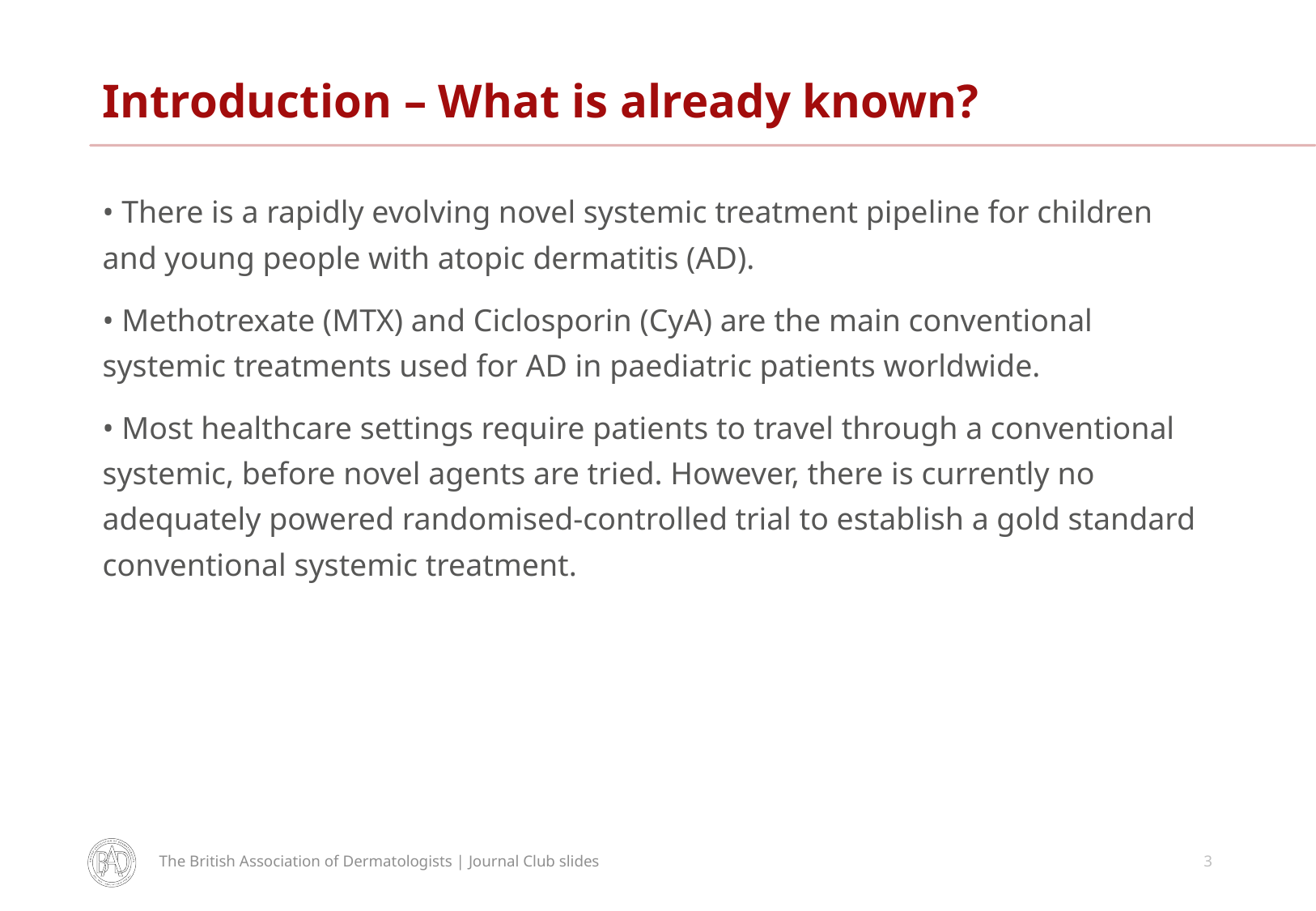

# Introduction – What is already known?
• There is a rapidly evolving novel systemic treatment pipeline for children and young people with atopic dermatitis (AD).
• Methotrexate (MTX) and Ciclosporin (CyA) are the main conventional systemic treatments used for AD in paediatric patients worldwide.
• Most healthcare settings require patients to travel through a conventional systemic, before novel agents are tried. However, there is currently no adequately powered randomised-controlled trial to establish a gold standard conventional systemic treatment.
The British Association of Dermatologists | Journal Club slides
3

## Slide 4
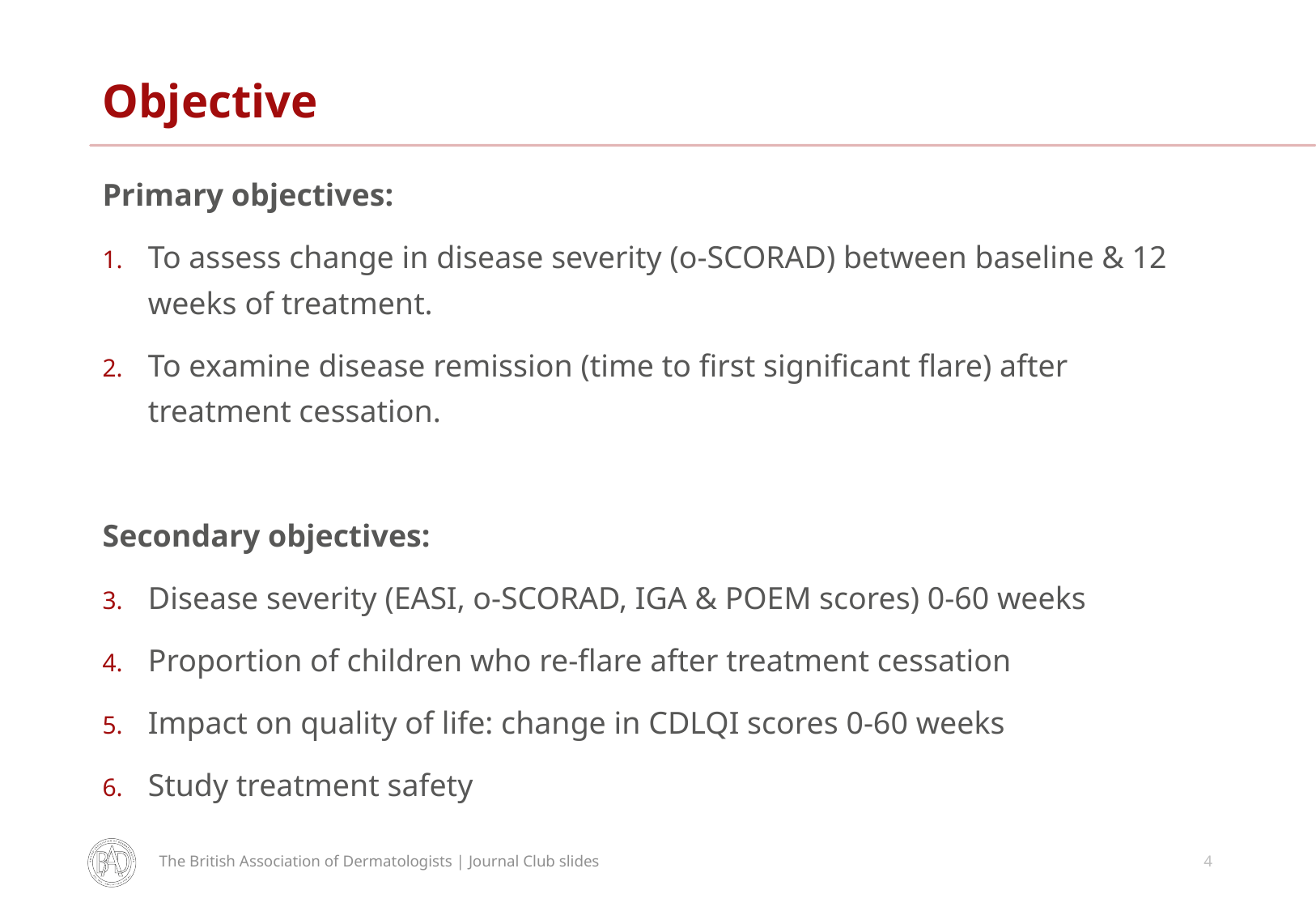

# Objective
Primary objectives:
To assess change in disease severity (o-SCORAD) between baseline & 12 weeks of treatment.
To examine disease remission (time to first significant flare) after treatment cessation.
Secondary objectives:
Disease severity (EASI, o-SCORAD, IGA & POEM scores) 0-60 weeks
Proportion of children who re-flare after treatment cessation
Impact on quality of life: change in CDLQI scores 0-60 weeks
Study treatment safety
The British Association of Dermatologists | Journal Club slides
4

## Slide 5
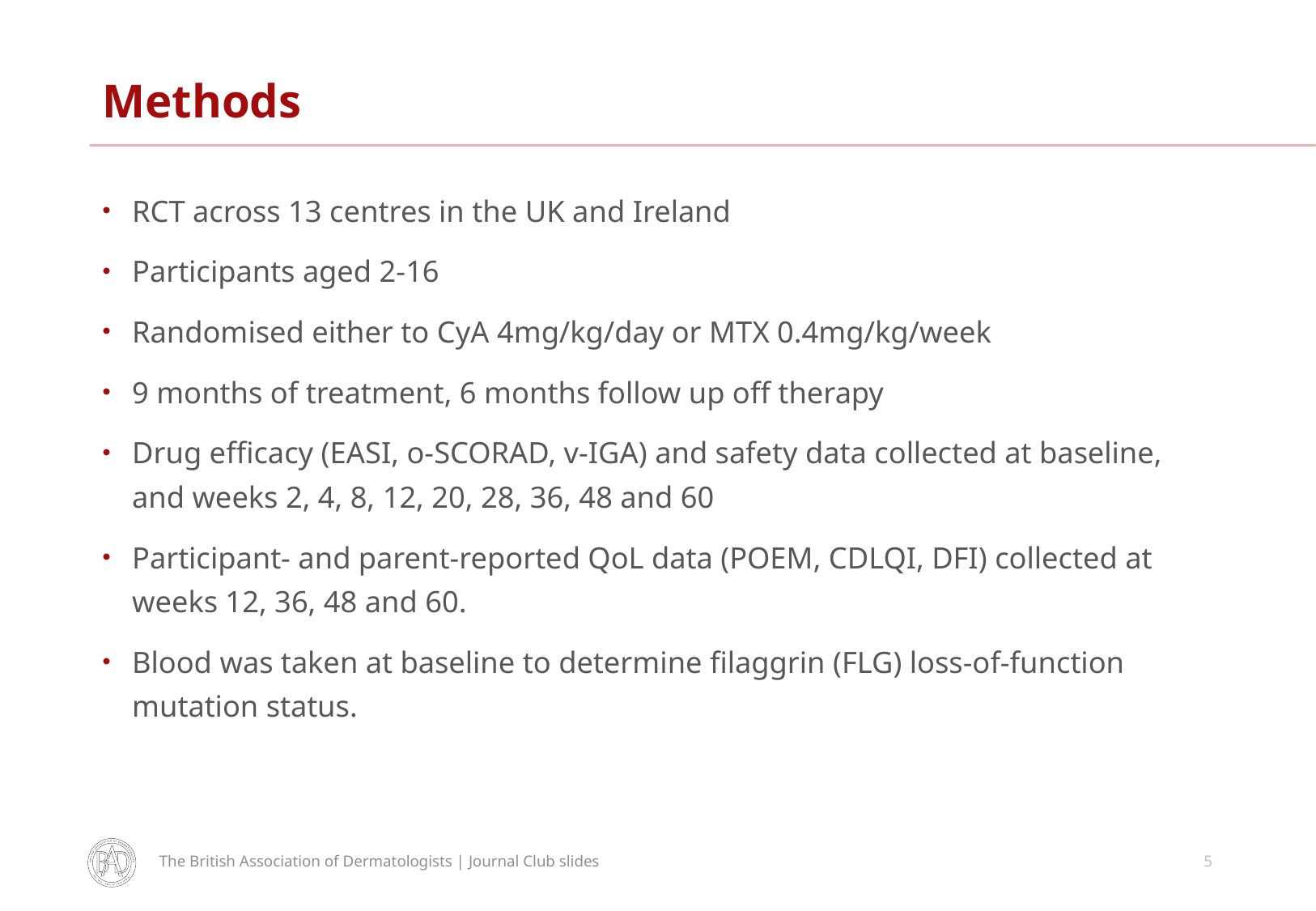

# Methods
RCT across 13 centres in the UK and Ireland
Participants aged 2-16
Randomised either to CyA 4mg/kg/day or MTX 0.4mg/kg/week
9 months of treatment, 6 months follow up off therapy
Drug efficacy (EASI, o-SCORAD, v-IGA) and safety data collected at baseline, and weeks 2, 4, 8, 12, 20, 28, 36, 48 and 60
Participant- and parent-reported QoL data (POEM, CDLQI, DFI) collected at weeks 12, 36, 48 and 60.
Blood was taken at baseline to determine filaggrin (FLG) loss-of-function mutation status.
The British Association of Dermatologists | Journal Club slides
5

## Slide 6
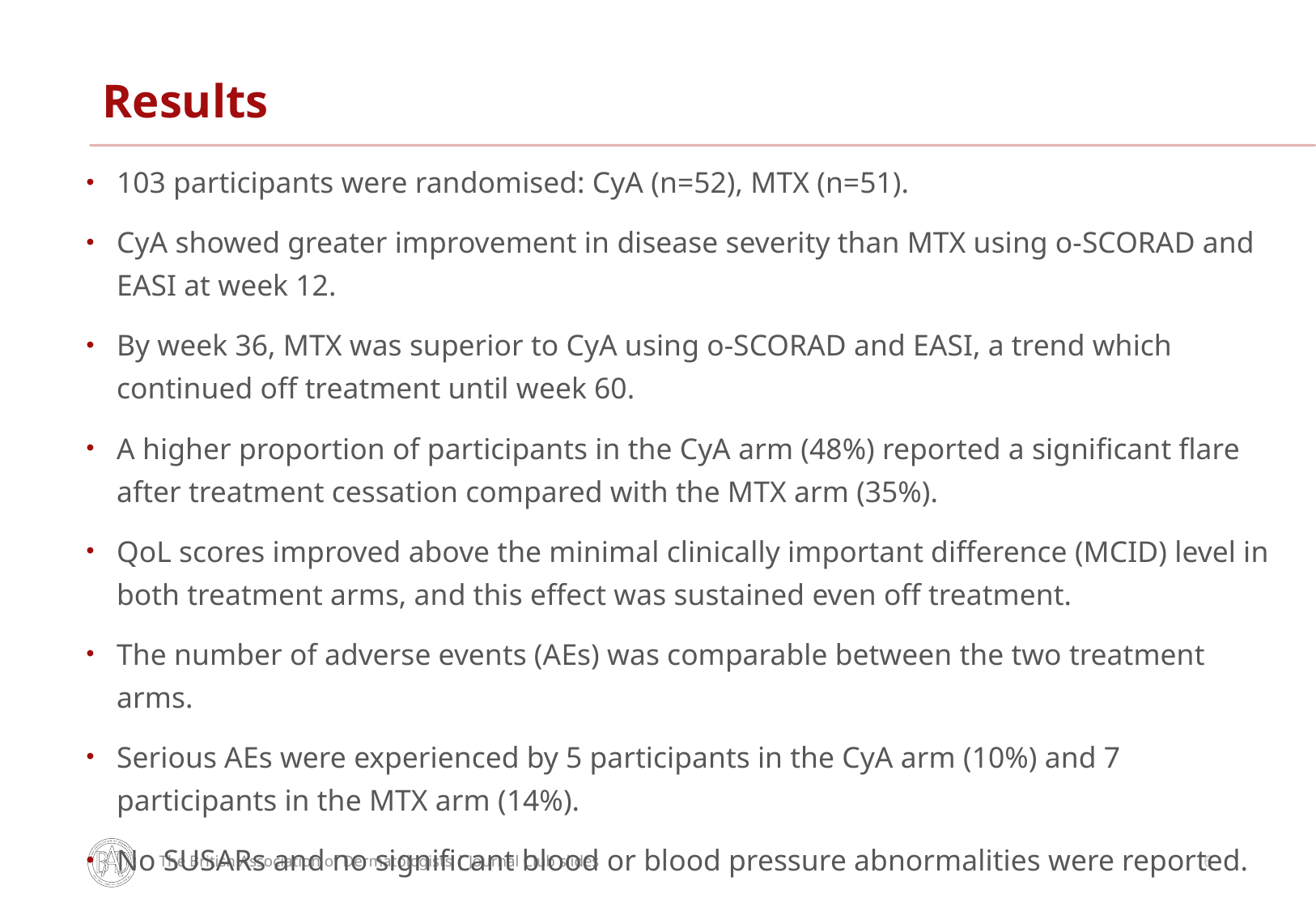

# Results
103 participants were randomised: CyA (n=52), MTX (n=51).
CyA showed greater improvement in disease severity than MTX using o-SCORAD and EASI at week 12.
By week 36, MTX was superior to CyA using o-SCORAD and EASI, a trend which continued off treatment until week 60.
A higher proportion of participants in the CyA arm (48%) reported a significant flare after treatment cessation compared with the MTX arm (35%).
QoL scores improved above the minimal clinically important difference (MCID) level in both treatment arms, and this effect was sustained even off treatment.
The number of adverse events (AEs) was comparable between the two treatment arms.
Serious AEs were experienced by 5 participants in the CyA arm (10%) and 7 participants in the MTX arm (14%).
No SUSARs and no significant blood or blood pressure abnormalities were reported.
The British Association of Dermatologists | Journal Club slides
6

## Slide 7
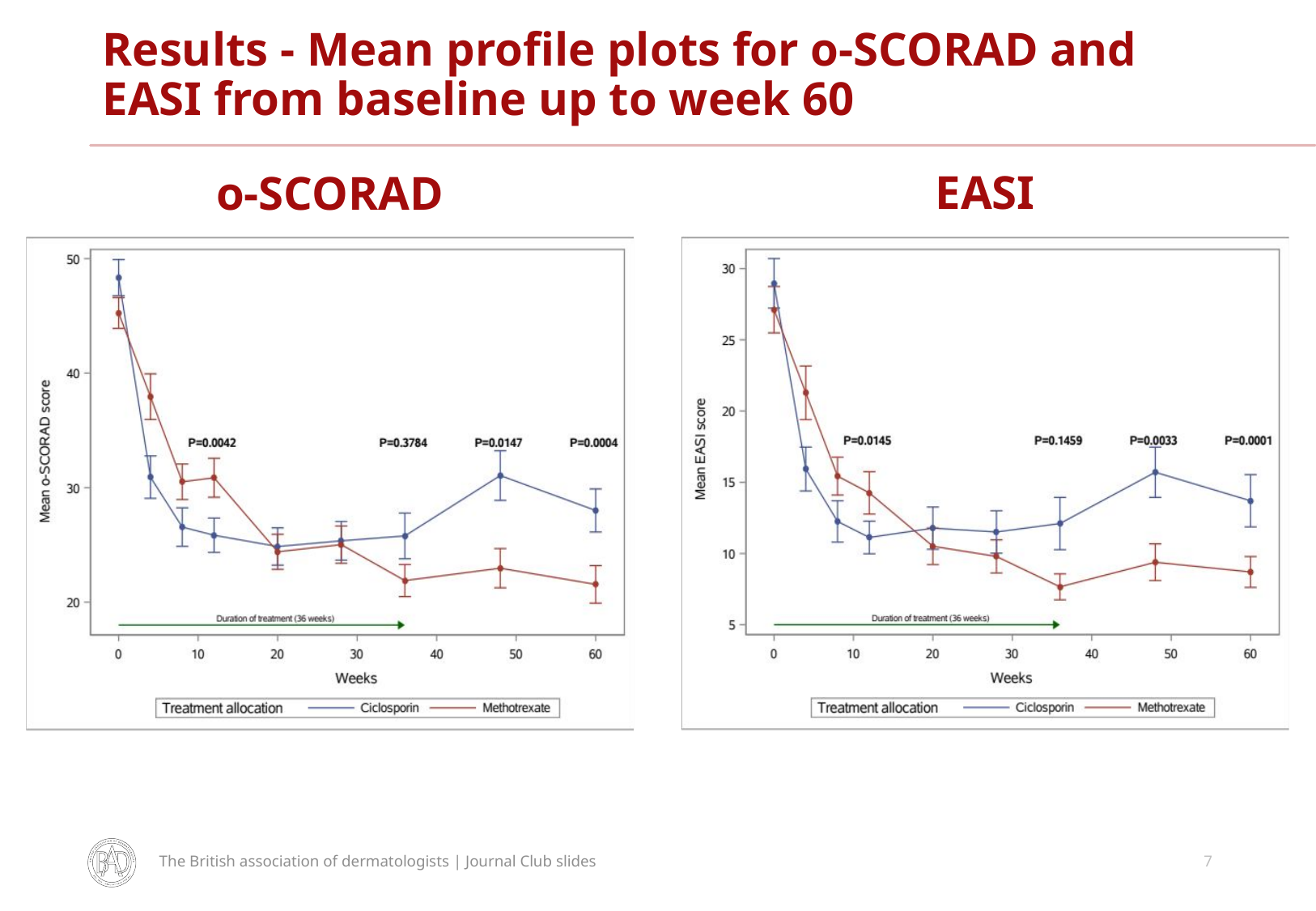

# Results - Mean profile plots for o-SCORAD and EASI from baseline up to week 60
EASI
o-SCORAD
The British association of dermatologists | Journal Club slides
7

## Slide 8
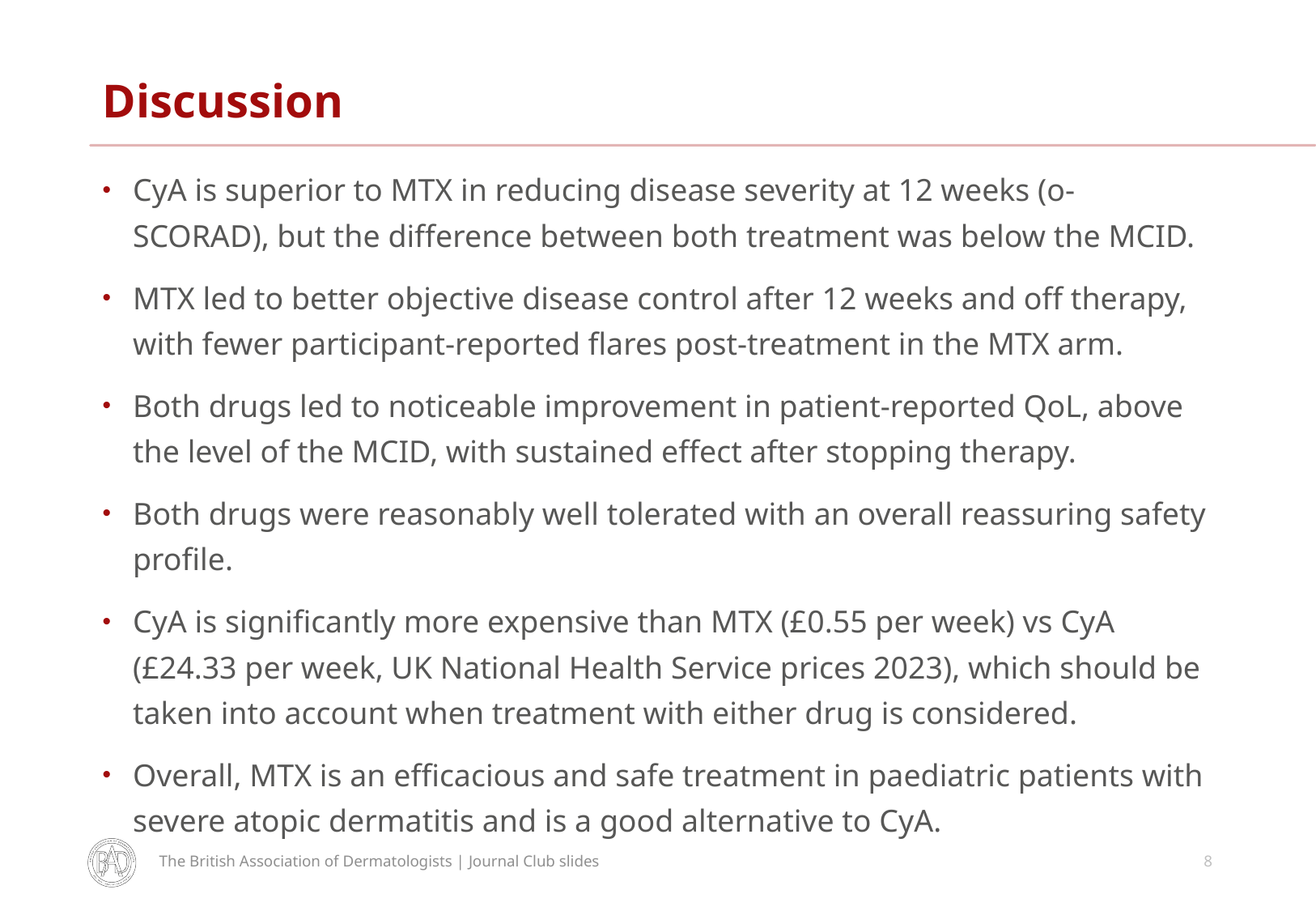

# Discussion
CyA is superior to MTX in reducing disease severity at 12 weeks (o-SCORAD), but the difference between both treatment was below the MCID.
MTX led to better objective disease control after 12 weeks and off therapy, with fewer participant-reported flares post-treatment in the MTX arm.
Both drugs led to noticeable improvement in patient-reported QoL, above the level of the MCID, with sustained effect after stopping therapy.
Both drugs were reasonably well tolerated with an overall reassuring safety profile.
CyA is significantly more expensive than MTX (£0.55 per week) vs CyA (£24.33 per week, UK National Health Service prices 2023), which should be taken into account when treatment with either drug is considered.
Overall, MTX is an efficacious and safe treatment in paediatric patients with severe atopic dermatitis and is a good alternative to CyA.
The British Association of Dermatologists | Journal Club slides
8

## Slide 9
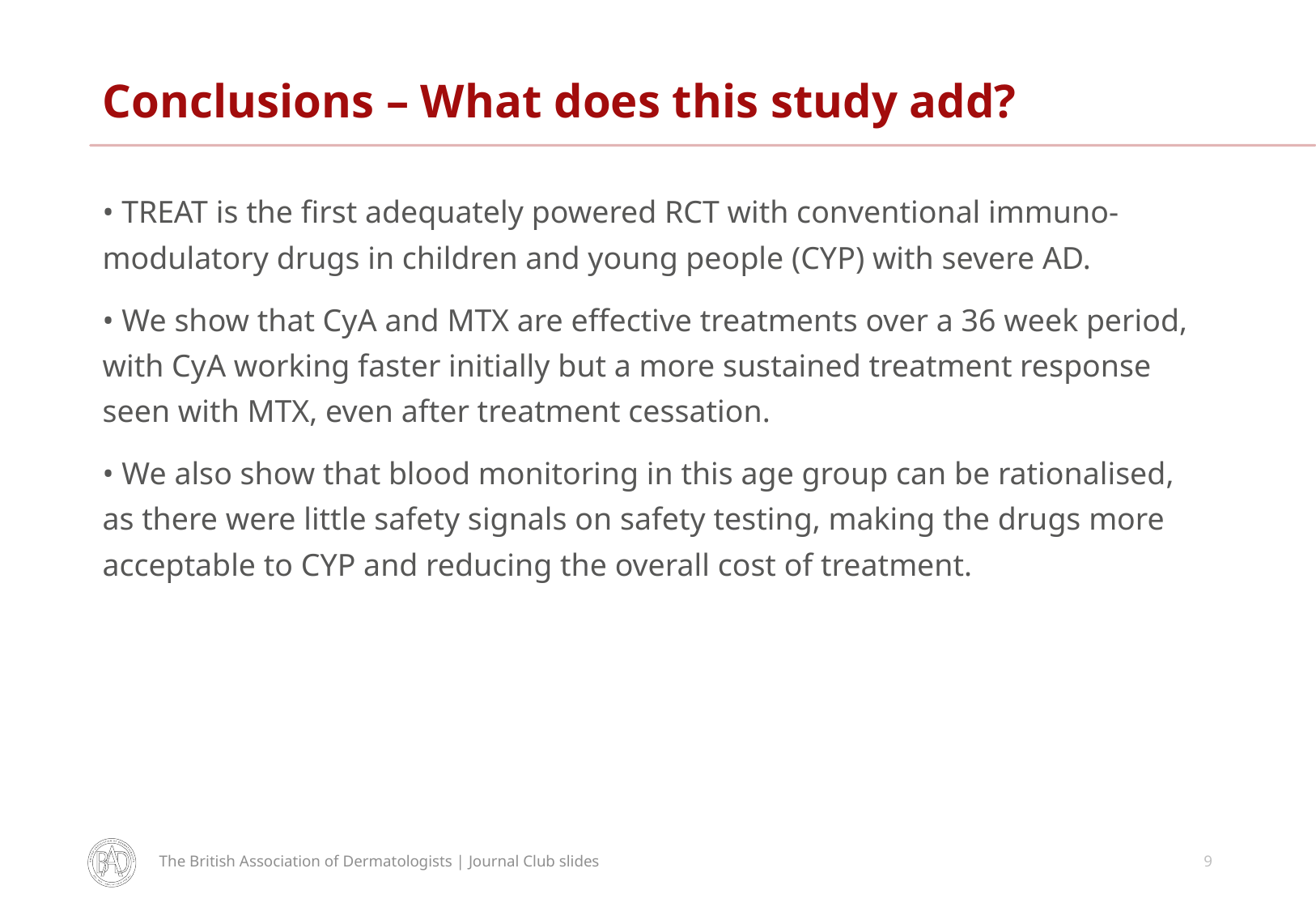

# Conclusions – What does this study add?
• TREAT is the first adequately powered RCT with conventional immuno-modulatory drugs in children and young people (CYP) with severe AD.
• We show that CyA and MTX are effective treatments over a 36 week period, with CyA working faster initially but a more sustained treatment response seen with MTX, even after treatment cessation.
• We also show that blood monitoring in this age group can be rationalised, as there were little safety signals on safety testing, making the drugs more acceptable to CYP and reducing the overall cost of treatment.
The British Association of Dermatologists | Journal Club slides
9

## Slide 10
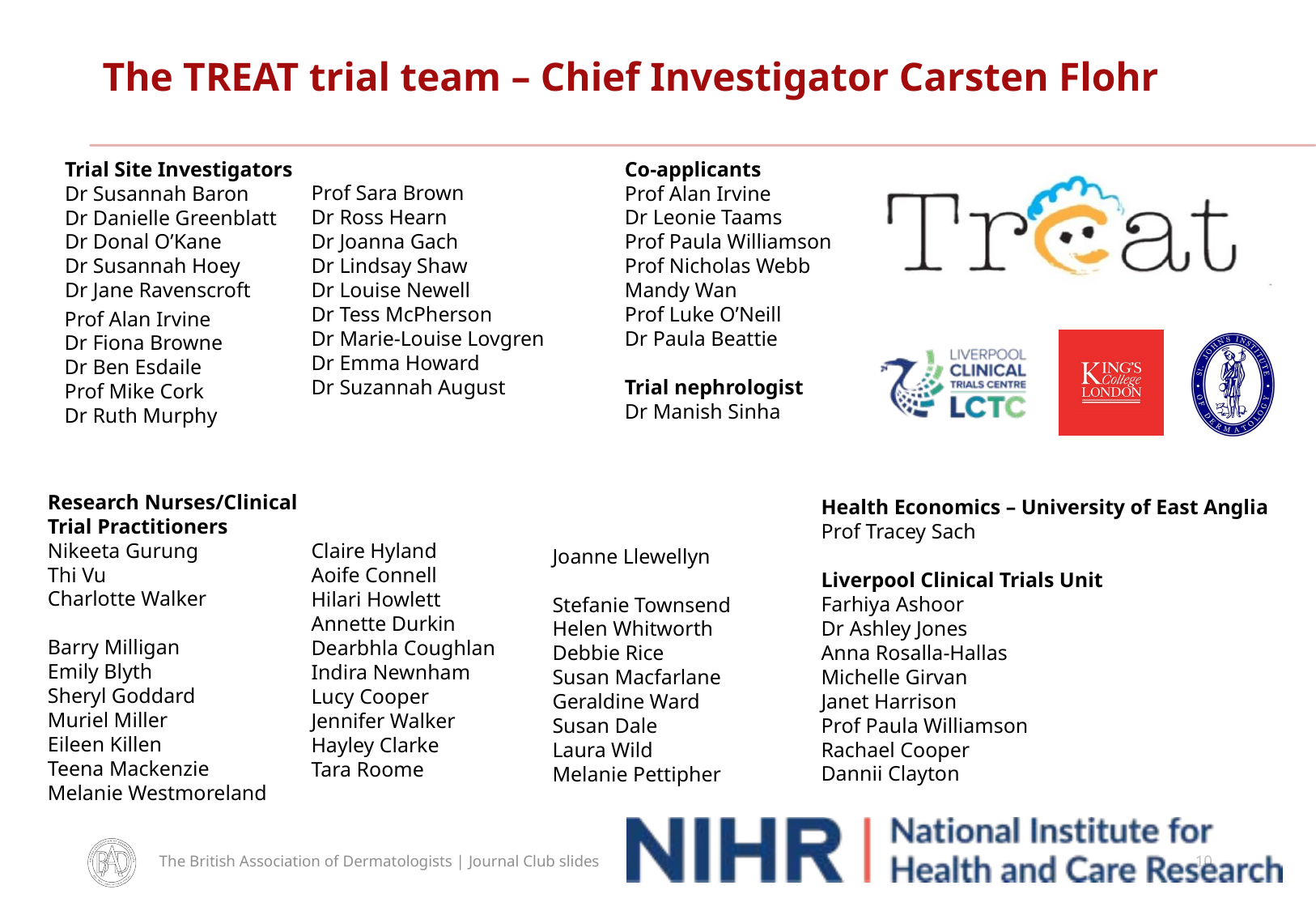

# The TREAT trial team – Chief Investigator Carsten Flohr
Trial Site Investigators
Dr Susannah Baron
Dr Danielle Greenblatt
Dr Donal O’Kane
Dr Susannah Hoey
Dr Jane Ravenscroft
Co-applicants
Prof Alan Irvine
Dr Leonie Taams
Prof Paula Williamson
Prof Nicholas Webb
Mandy Wan
Prof Luke O’Neill
Dr Paula Beattie
Trial nephrologist
Dr Manish Sinha
Prof Sara Brown
Dr Ross Hearn
Dr Joanna Gach
Dr Lindsay Shaw
Dr Louise Newell
Dr Tess McPherson
Dr Marie-Louise Lovgren
Dr Emma Howard
Dr Suzannah August
Prof Alan Irvine
Dr Fiona Browne
Dr Ben Esdaile
Prof Mike Cork
Dr Ruth Murphy
Research Nurses/Clinical Trial Practitioners
Nikeeta Gurung
Thi Vu
Charlotte Walker
Barry Milligan
Emily Blyth
Sheryl Goddard
Muriel Miller
Eileen Killen
Teena Mackenzie
Melanie Westmoreland
Health Economics – University of East Anglia
Prof Tracey Sach
Liverpool Clinical Trials Unit
Farhiya Ashoor
Dr Ashley Jones
Anna Rosalla-Hallas
Michelle Girvan
Janet Harrison
Prof Paula Williamson
Rachael Cooper
Dannii Clayton
Claire Hyland
Aoife Connell
Hilari Howlett
Annette Durkin
Dearbhla Coughlan
Indira Newnham
Lucy Cooper
Jennifer Walker
Hayley Clarke
Tara Roome
Joanne Llewellyn
Stefanie Townsend
Helen Whitworth
Debbie Rice
Susan Macfarlane
Geraldine Ward
Susan Dale
Laura Wild
Melanie Pettipher
The British Association of Dermatologists | Journal Club slides
10

## Slide 11
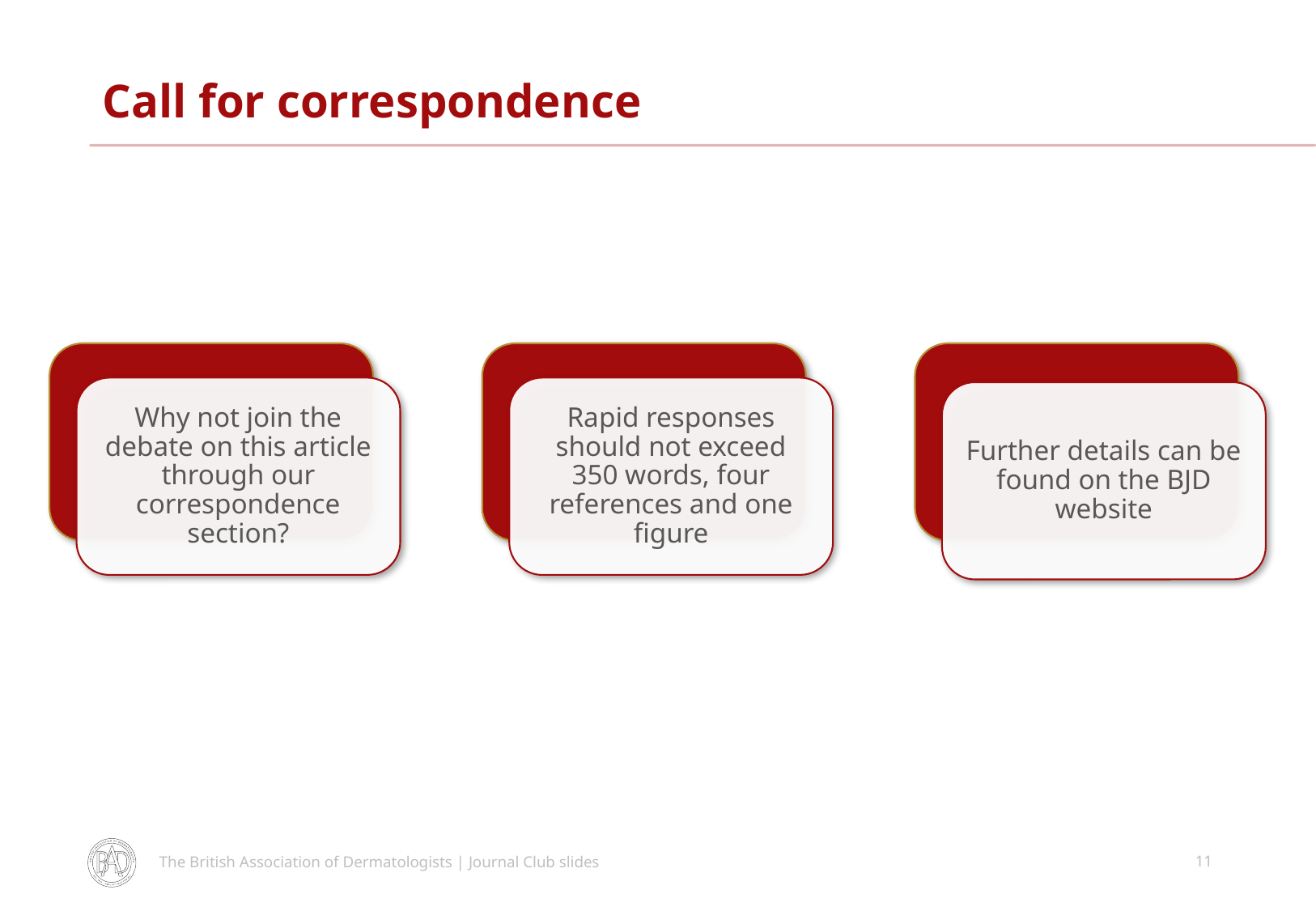

# Call for correspondence
Why not join the debate on this article through our correspondence section?
Rapid responses should not exceed 350 words, four references and one figure
Further details can be found on the BJD website
The British Association of Dermatologists | Journal Club slides
11
