# Supplementary material for: Efficacy and safety of ciclosporin versus methotrexate in the treatment of severe atopic dermatitis in children and young people (TREAT): a multicentre parallel group assessor-blinded clinical trial
Source: Br J Dermatol. 2023 Sep 19;189(6):674–84. doi: 10.1093/bjd/ljad281 (PMC13077216; doi:10.1093/bjd/ljad281)
Supplement: ljad281_Supplementary_Data [file ljad281_supplementary_data.zip › Supporting information.docx]

**Contributors:**

The TREAT trial was led by CF (Chief Investigator). CF chaired the Trial Management Group. FA was the trial manager, supervised by CS. The statistical analyses were conducted by AR-H (statistician), supervised by APJ (lead statistician), with contributions from PRW, who was also the Director of the Liverpool Clinical Trials Centre during most of the trial period. AR-H produced the tables and figures. CF, AI, PRW, PB, FB, and LT contributed to the study design and were co-applicants on the trial grant application. CF and EH produced the first draft, together with AI, AR-H and APJ. All co-authors contributed to the data interpretation and writing. AR-H and APJ directly accessed and verified the underlying data reported in the manuscript.

**Trial Sponsor:**

Guy’s and St Thomas’ (GST) NHS Foundation Trust, Guy’s Hospital, Great Maze Pond, London SE1 9RT, and King’s College London (KCL), Strand, London, WC2R 2 LS

The funder and sponsor both approved protocol amendments prior to submission for ethical/regulatory approval. The funder and sponsor did not have a role in the analyses and interpretation of the data or the decision to submit the results.

**Role of trial sponsor:**

GST and KCL agreed to take on the joint role of Co-sponsor for the study. The Co-Sponsors acted as Sponsor for the trial under the Research Governance Framework for Health and Social Care, and the Medicines for Human Use (Clinical Trial) Regulations 2004 and Amended Regulations 2006. The Co-Sponsors delegated trial management activities to the

King’s Health Partners Clinical Trials Office (KHP-CTO) and the Liverpool Clinical Trials Centre (LCTC), University of Liverpool. The sponsors at all times maintained adequate insurance in relation to the study.

**Role and responsibilities of the Trial Coordinating Centre:**

TREAT was co-ordinated by the LCTC at the University of Liverpool. The Co-Sponsors delegated the trial management activities along with trial data management, statistics and information systems to the LCTC.

**Role and responsibilities of the Trial Oversight Committees:**

The study had an independent Trial Steering Committee (TSC) (comprising independent members as well as the Chief Investigator as voting members) and an Independent Data & Safety Monitoring Committee (IDSMC). Day to day oversight was conducted by the Trial Management Group (TMG), chaired by the Chief Investigator. The roles and responsibilities of the committees were defined in the TSC Terms of Reference and the IDSMC Charter, available upon request.

**Trial Steering Committee members:**

Independent members: Alison Layton (Chair, Consultant Dermatologist & Associate Medical Director for Research); Tim Burton (Patient & Public Representative); Michael Grainge (Statistician); Michael Arden-Jones (Dermatologist); Saskia King (Patient & Public Representative); Michael Perkin (Consultant Paediatric Allergist); Alain Taieb (Paediatric Dermatologist). Non-independent member: Carsten Flohr (Chief Investigator),

**Independent Data and Safety Monitoring Committee members:**

Anthony Ormerod (Chair, Emeritus Professor in Dermatology, University of Aberdeen and Honorary Consultant Dermatologist NHS Grampian); Robert Chalmers (Honorary Consultant Dermatologist, Co-Chair and Managing Editor, Dermatology Topic Advisory Group, WHO ICD Revision Project); Xinxue Liu (Honorary Research Fellow).

**Trial Management Group members:** Amina Ahmed (Patient & Public Representative); Farhiya Ashoor (Trial Manager); Carsten Flohr (Chief Investigator, Chair); Anna Rosala-Hallas (Trial Statistician); Amy Holton and Hannah Mason (Sponsor Representatives); Alan Irvine (Principal Investigator); Ashley Jones (Lead Statistician), Tracey Sach (Health Economist); Catherine Spowart (Supervising Trial Manager); Mandy Wan (Lead Pharmacist); Charlotte Walker (Lead Research Nurse)

**Principal Investigators:** Suzannah August (Poole Hospital); Paula Beattie (Royal Hospital for Children, Glasgow); Sara Brown (Ninewells Hospital, Dundee); Mike Cork (Sheffield Children’s Hospital); Ben Esdaile (Whittington Hospital); Hospital); Carsten Flohr (Guy’s & St Thomas’ Hospital); Joanna Gach (University Hospitals Coventry & Warwickshire); Emma Howard (Birmingham Children’s Hospital); Alan Irvine (Our Lady’s Children’s Hospital, Dublin); Tess McPherson (Oxford University Hospitals); Donal O’Kane (Royal Belfast Hospital for Sick Children, Belfast); Jane Ravenscroft (Nottingham University Hospitals); Lindsay Shaw (Bristol Royal Hospital for Children).

**Co-Investigators:** Caroline Allen (Oxford University Hospitals); Susannah Baron (Guy’s & St Thomas’ Hospital); Danielle Greenblatt (Guy’s & St Thomas’ Hospital); Robert Hearn (Ninewells Hospital, Dundee); Susannah Hoey (Royal Belfast Hospital for Sick Children, Belfast); Rachael Jarret (Oxford University Hospitals); Catherine Jury (Royal Hospital for Children, Glasgow); Charlie Mitchell (Poole Hospital); Ruth Murphy (Sheffield Children’s Hospital); Graham Ogg (Oxford University Hospitals); Alice Plant (Poole Hospital); Louise Newell (Bristol Royal Hospital for Children); Jothsana Srinivasan (Nottingham University Hospitals), Emma Wedgeworth (Guy’s & St Thomas’ Hospital) Fiona Browne (CHI at Crumlin).
