# Supplementary material for: Efficacy and safety of ciclosporin versus methotrexate in the treatment of severe atopic dermatitis in children and young people (TREAT): a multicentre parallel group assessor-blinded clinical trial
Source: Br J Dermatol. 2023 Sep 19;189(6):674–84. doi: 10.1093/bjd/ljad281 (PMC13077216; doi:10.1093/bjd/ljad281)
Supplement: ljad281_Supplementary_Data [file ljad281_supplementary_data.zip › TREAT paper - Supplementary Appendix_FINAL_tc.docx]

**Supplementary Appendix**

**Contents**

Statistical analysis for secondary outcomes

Supplementary Table 1: Co-primary outcome 2 – Sensitivity analysis 1

Supplementary Table 2: Co-primary outcome 2 – Sensitivity analysis 2

Supplementary Table 3: Co-primary outcome 2 – Sensitivity analysis 3

Supplementary Table 4: POEM results from a linear mixed model

Supplementary Table 5: o-SCORAD-50, o-SCORAD-75 and o-SCORAD-90 – Generalised Linear Mixed Model Results

Supplementary Table 6: Linear regression results exploring treatment modification effect of FLG carriage

Supplementary Table 7: EASI-50, EASI-75 and EASI-90 – Generalised Linear Mixed Model Results [Post-hoc]

Supplementary Table 8: Proportion of participants achieving IGA 0 or 1 at each visit

Supplementary Table 9: CDLQI Linear Mixed Model Results
Supplementary Table 10: DFI Linear Mixed Model Results

Supplementary Table 11: Non-serious Adverse Events

Supplementary Table 12: Participants using anti-inflammatory concomitant medications during and after the treatment phase

Supplementary Figure 1. Kaplan-Meier curve comparing the time to significant re-flares of disease in the two treatment arms (CyA (blue) vs MTX (red)).

Supplementary Figure 2: Mean profile plots for IGA over time
Supplementary Figure 3: Mean profile plots for POEM over time
Supplementary Figure 4. Mean profile plot of CDLQI over time

Supplementary Figure 5. Mean profile plot of IDQoL over time

Supplementary Figure 6. Mean profile plot of DFI over time

TREAT Statistical Analysis Plan

TREAT Protocol v7.0 29/07/2020

**Statistical analysis for secondary outcomes**

For the secondary outcomes all results are presented with 95% confidence intervals, Linear Mixed Models (LMM) were used to compare the EASI, IGA, o-SCORAD, o-SCORAD-50, POEM, CDLQI/IDQOL and DFI scores between treatment groups over time. The models were adjusted for each respective baseline score and included an interaction between time and treatment allocation. A t-test was used to compare the mean number of participant-reported flares between treatment groups following trial treatment cessation. A Mann-Whitney test was used to compare the median number of parent-reported flares resulting in change (in frequency or strength) in topical corticosteroid or calcineurin inhibitor treatment between treatment groups. Analyses of parent-reported flare data were restricted to those who completed more than 80% of each respective question within the participant diaries during the 24 weeks following treatment cessation. Fisher’s Exact Test was conducted to compare the number of participants who withdrew from treatment because of adverse events (AEs). Linear regression models, including an interaction between treatment group and *FLG* mutation carriage (Yes/No), were used to explore whether *FLG* inheritance modified the treatment effect at 12, 36 and 60 weeks. A post-hoc analysis was conducted using generalised LMMs to give estimated odds ratios for the proportion of participants achieving EASI-50/-75/-90, o-SCORAD -75/-90 at 12, 36, 48, and 60 weeks.

Supplementary Table 1: Co-primary outcome 2 – Sensitivity analysis 1^1^

| **Treatment** | **N** | **Flare**  **N (%)** | **Censored**  **N (%)** | **Log-rank**  **Chi-square statistic** | **Log-rank**  **p-value** |
| --- | --- | --- | --- | --- | --- |
| Ciclosporin | 45 | 21 (47%) | 24 (53%) | 1.68 | 0.1948 |
| Methotrexate | 38 | 14 (37%) | 24 (63%) |  |  |
| **Total** | **83** | **35** | **48** |  |  |

^1^Sensitivity analysis 1 included only those who completed 36 weeks of treatment.

Supplementary Table 2: Co-primary outcome 2 – Sensitivity analysis 2^1^

| **Treatment** | **N** | **Flare**  **N (%)** | **Censored**  **N (%)** | **Log-rank**  **Chi-square statistic** | **Log-rank**  **p-value** |
| --- | --- | --- | --- | --- | --- |
| Ciclosporin | 52 | 25 (48%) | 27 (52%) | 2.03 | 0.1538 |
| Methotrexate | 51 | 18 (35%) | 33 (65%) |  |  |
| **Total** | **103** | **43** | **60** |  |  |

^1^In sensitivity analysis 2 those who stopped trial treatment early but continued to take MTX or CyA begin the 24 week follow-up period from the end date of the MTX or CyA as recorded on the con-med form

**Supplementary Table 3: Co-primary outcome 2 – Sensitivity analysis 3**

| **Treatment** | **N** | **Flare**  **N (%)** | **Censored**  **N (%)** | **HR (97.5% CI)** | **p-value** |
| --- | --- | --- | --- | --- | --- |
| Ciclosporin | 52 | 25 (48%) | 27 (52%) | 1.54 (0.77, 3.09) | 0.1606 |
| Methotrexate | 51 | 18 (35%) | 33 (65%) |  |  |
| **Total** | **103** | **43** | **60** |  |  |

Supplementary Table 4: POEM results from a linear mixed model^1^

| **Time (weeks)** | **CYA - Estimated mean POEM score (SE)** | **MTX - Estimated mean POEM score (SE)** | **Estimated difference in means (SE)** | **95% CI** | **P-value** |
| --- | --- | --- | --- | --- | --- |
| 12 | 9.28 (0.72) | 12.01 (0.72) | -2.73 (1.02) | -4.75, -0.71 | 0.0085 |
| 36 | 10.10 (0.72) | 9.89 (0.72) | 0.22 (1.01) | -1.79, 2.23 | 0.8308 |
| 48 | 10.52 (0.73) | 8.82 (0.73) | 1.69 (1.03) | -0.35, 3.74 | 0.1040 |
| 60 | 10.93 (0.75) | 7.76 (0.75) | 3.17 (1.06) | 1.06, 5.28 | 0.0035 |

^1^50 CyA and 49 MTX participants were included in the model

Supplementary Table 5: o-SCORAD-50, o-SCORAD-75 and o-SCORAD-90 – Generalised Linear Mixed Model Results

| **Visit** | **CyA**  **(N=52)** | | **MTX**  **(N=51)** | | **Estimated OR (95% CI)** | **P-value** |
| --- | --- | --- | --- | --- | --- | --- |
|  | **N** | **≥50% improvement** | **N** | **≥50% improvement** |  |  |
| Week 12 | 52 | 25 (48.1%) | 51 | 14 (27.5%) | 2.60 (1.23,5.49) | 0.0124 |
| Week 36 | 48 | 27 (56.3%) | 46 | 24 (52.2%) | 0.93 (0.46,1.88) | 0.8455 |
| Week 48 | 47 | 16 (34.0%) | 45 | 23 (51.1%) | 0.56 (0.25,1.23) | 0.1488 |
| Week 60 | 46 | 22 (47.8%) | 44 | 24 (54.5%) | 0.33 (0.13,0.85) | 0.0216 |

| **[Post-hoc]** | **≥75% improvement** | | **≥75% improvement** | |  |  |
| --- | --- | --- | --- | --- | --- | --- |
| Week 12 | 52 | 6 (11.5%) | 51 | 1 (2.0%) | 2.89 (0.77,10.84) | 0.1157 |
| Week 16^1^ | - | - | - | - | 2.31 (0.66,8.11) | 0.1919 |
| Week 36 | 48 | 5 (10.4%) | 46 | 7 (15.2%) | 0.75 (0.24,2.32) | 0.6169 |
| Week 48 | 47 | 3 (6.4%) | 45 | 6 (13.3%) | 0.38 (0.11,1.31) | 0.1262 |
| Week 60 | 46 | 3 (6.5%) | 44 | 7 (15.9%) | 0.19 (0.05,0.83) | 0.0268 |

| **[Post-hoc]** | **≥90% improvement** | | **≥90% improvement** | |  |  |
| --- | --- | --- | --- | --- | --- | --- |
| Week 12 | 52 | 0 (0.0%) | 51 | 0 (0.0%) | 2.60 (1.23,5.49) | 0.0124 |
| Week 16^1^ | - | - | - | - | 0.93 (0.46,1.88) | 0.8455 |
| Week 36 | 48 | 1 (2.1%) | 46 | 2 (4.3%) | 0.56 (0.25,1.23) | 0.1488 |
| Week 48 | 47 | 0 (0.0%) | 45 | 1 (2.2%) | 0.33 (0.13,0.85) | 0.0216 |
| Week 60 | 46 | 0 (0.0%) | 44 | 1 (2.3%) | 2.60 (1.23,5.49) | 0.0124 |

^1^There was no Week 16 visit, ORs for Week 16 are estimated by the GLMM

Supplementary Table 6: Linear regression results exploring treatment modification effect of FLG carriage

| **Variable** | **Parameter Estimate (SE)** | **95% Confidence Interval** | **P-value** |
| --- | --- | --- | --- |
| **EASI 12 weeks** | | | |
| Intercept | 2.417 ( 0.421 ) | 1.58 , 3.253 | <0.001 |
| Baseline EASI score | 0.048 ( 0.012 ) | 0.025 , 0.071 | <0.001 |
| FLG carriage | -0.413 ( 0.373 ) | -1.154 , 0.328 | 0.271 |
| CyA vs MTX | -0.644 ( 0.357 ) | -1.354 , 0.067 | 0.075 |
| FLG carriage in CyA vs FLG carriage in MTX | 0.406 ( 0.514 ) | -0.615 , 1.428 | 0.431 |
| **EASI 36 weeks** | | | |
| Intercept | 0.799 ( 0.416 ) | -0.028 , 1.627 | 0.058 |
| Baseline EASI score | 0.062 ( 0.011 ) | 0.039 , 0.085 | <0.001 |
| FLG carriage | 0.239 ( 0.375 ) | -0.508 , 0.986 | 0.526 |
| CyA vs MTX | 0.205 ( 0.362 ) | -0.515 , 0.925 | 0.572 |
| FLG carriage in CyA vs FLG carriage in MTX | 0.441 ( 0.517 ) | -0.587 , 1.47 | 0.396 |
| **EASI 60 weeks** | | | |
| Intercept | 1.736 ( 0.478 ) | 0.784 , 2.688 | 0.001 |
| Baseline EASI score | 0.035 ( 0.013 ) | 0.009 , 0.062 | 0.01 |
| FLG carriage | -0.27 ( 0.435 ) | -1.137 , 0.597 | 0.536 |
| CyA vs MTX | 0.877 ( 0.422 ) | 0.037 , 1.718 | 0.041 |
| FLG carriage in CyA vs FLG carriage in MTX | -0.024 ( 0.596 ) | -1.212 , 1.163 | 0.968 |
| **o-SCORAD 12 weeks** | | | |
| Intercept | 20.313 ( 6.363 ) | 7.66 , 32.967 | 0.002 |
| Baseline o-SCORAD | 0.238 ( 0.123 ) | -0.006 , 0.483 | 0.056 |
| FLG carriage | -1.871 ( 3.566 ) | -8.962 , 5.219 | 0.601 |
| CyA vs MTX | -6.838 ( 3.416 ) | -13.631 , -0.046 | 0.049 |
| FLG carriage in CyA vs FLG carriage in MTX | 3.915 ( 4.916 ) | -5.86 , 13.69 | 0.428 |
| **o-SCORAD 36 weeks** | | | |
| Intercept | 1.656 ( 6.337 ) | -10.963 , 14.275 | 0.795 |
| Baseline o-SCORAD | 0.403 ( 0.122 ) | 0.161 , 0.645 | 0.001 |
| FLG carriage | 2.244 ( 3.661 ) | -5.046 , 9.534 | 0.542 |
| CyA vs MTX | 1.922 ( 3.51 ) | -5.067 , 8.91 | 0.586 |
| FLG carriage in CyA vs FLG carriage in MTX | 3.297 ( 5.018 ) | -6.696 , 13.289 | 0.513 |
| **o-SCORAD 60 weeks** | | | |
| Intercept | 13.904 ( 6.686 ) | 0.582 , 27.226 | 0.041 |
| Baseline o-SCORAD | 0.14 ( 0.131 ) | -0.121 , 0.401 | 0.288 |
| FLG carriage | -0.435 ( 3.907 ) | -8.22 , 7.35 | 0.912 |
| CyA vs MTX | 8.827 ( 3.784 ) | 1.287 , 16.366 | 0.022 |
| FLG carriage in CyA vs FLG carriage in MTX | -0.322 ( 5.34 ) | -10.962 , 10.318 | 0.952 |

Supplementary Table 7: EASI-50, EASI-75 and EASI-90 – Generalised Linear Mixed Model Results [Post-hoc]

| **Visit** | **CyA**  **(N=52)** | | **MTX**  **(N=51)** | | **Estimated OR (95% CI)** | **P-value** |
| --- | --- | --- | --- | --- | --- | --- |
|  | **N** | **≥50% improvement** | **N** | **≥50% improvement** |  |  |
| Week 12 | 52 | 35 (67.3%) | 51 | 27 (52.9%) | 1.77 (0.92,3.42) | 0.0888 |
| Week 16^1^ | - | - | - | - | 1.47 (0.78,2.76) | 0.2362 |
| Week 36 | 48 | 34 (70.8%) | 46 | 40 (87.0%) | 0.57 (0.29,1.11) | 0.096 |
| Week 48 | 47 | 23 (48.9%) | 45 | 35 (77.8%) | 0.32 (0.15,0.71) | 0.0051 |
| Week 60 | 46 | 32 (69.6%) | 44 | 34 (77.3%) | 0.18 (0.07,0.48) | 0.0006 |

|  | **≥75% improvement** | | **≥75% improvement** | |  |  |
| --- | --- | --- | --- | --- | --- | --- |
| Week 12 | 52 | 23 (44.2%) | 51 | 10 (19.6%) | 2.66 (1.37,5.19) | 0.0040 |
| Week 16^1^ | - | - | - | - | 2.24 (1.19,4.22) | 0.0125 |
| Week 36 | 48 | 20 (41.7%) | 46 | 21 (45.7%) | 0.94 (0.52,1.71) | 0.8487 |
| Week 48 | 47 | 14 (29.8%) | 45 | 21 (46.7%) | 0.56 (0.28,1.11) | 0.0988 |
| Week 60 | 46 | 16 (34.8%) | 44 | 21 (47.7%) | 0.33 (0.15,0.77) | 0.0097 |

|  | **≥90% improvement** | | **≥90% improvement** | |  |  |
| --- | --- | --- | --- | --- | --- | --- |
| Week 12 | 52 | 4 (7.7%) | 51 | 1 (2.0%) | 4.19 (1.39,12.67) | 0.0113 |
| Week 16^1^ | - | - | - | - | 3.19 (1.12,9.05) | 0.0295 |
| Week 36 | 48 | 5 (10.4%) | 46 | 9 (19.6%) | 0.81 (0.32,2.08) | 0.6667 |
| Week 48 | 47 | 3 (6.4%) | 45 | 7 (15.6%) | 0.36 (0.12,1.04) | 0.0587 |
| Week 60 | 46 | 2 (4.3%) | 44 | 7 (15.9%) | 0.16 (0.04,0.57) | 0.0051 |

^1^There was no Week 16 visit, ORs for Week 16 are estimated by the GLMM

Supplementary Table 8: Proportion of participants achieving IGA 0 or 1 at each visit

| **Visit** | **CyA** | | **MTX** | |
| --- | --- | --- | --- | --- |
|  | **N** | **IGA 0/1, N (%)** | **N** | **IGA 0/1, N (%)** |
| Week 12 | 52 | 6 (11.54%) | 51 | 1 (1.96%) |
| Week 36 | 48 | 7 (14.58%) | 46 | 7 (15.22%) |
| Week 48 | 47 | 3 (6.38%) | 45 | 8 (17.78%) |
| Week 60 | 45 | 4 (8.89%) | 44 | 9 (20.45%) |

Supplementary Table 9: CDLQI Linear Mixed Model Results

| **Time (weeks)** | **CyA** | | **MTX** | | **Estimated difference in means (SE)** | **95% CI** | **P-value** |
| --- | --- | --- | --- | --- | --- | --- | --- |
|  | **N** | **Estimated mean CDLQI score (SE)** | **N** | **Estimated mean CDLQI score (SE)** |  |  |  |
| 12 | 49 | 7.09 (0.76) | 49 | 8.45 (0.76) | -1.36 (1.08) | -3.49, 0.77 | 0.2085 |
| 36 | 48 | 7.63 (0.58) | 42 | 7.80 (0.59) | -0.17 (0.83) | -1.82, 1.48 | 0.8345 |
| 48 | 46 | 7.90 (0.61) | 41 | 7.48 (0.62) | 0.42 (0.87) | -1.30, 2.14 | 0.6299 |
| 60 | 47 | 8.17 (0.70) | 43 | 7.15 (0.72) | 1.01 (1.01) | -0.98, 3.01 | 0.3164 |

Supplementary Table 10: DFI Linear Mixed Model Results

| **Time (weeks)** | **CyA** | | **MTX** | | **Estimated difference in means (SE)** | **95% CI** | **P-value** |
| --- | --- | --- | --- | --- | --- | --- | --- |
|  | **N** | **Estimated mean DFI score (SE)** | **N** | **Estimated mean DFI score (SE)** |  |  |  |
| 12 | 51 | 7.59 (0.88) | 51 | 8.56 (0.88) | -0.96 (1.25) | -3.42, 1.49 | 0.4394 |
| 36 | 48 | 8.50 (0.73) | 44 | 7.96 (0.73) | 0.54 (1.04) | -1.51, 2.59 | 0.6035 |
| 48 | 47 | 8.95 (0.75) | 44 | 7.66 (0.76) | 1.29 (1.07) | -0.82, 3.41 | 0.2288 |
| 60 | 47 | 9.40 (0.83) | 44 | 7.36 (0.85) | 2.04 (1.19) | -0.30, 4.39 | 0.0873 |

Supplementary Table 11: Non-serious Adverse Events

| **System Organ Class** | **Preferred Term** | **CyA**  **(N=51)** | | **MTX**  **(N=51)** | |
| --- | --- | --- | --- | --- | --- |
|  |  | **Events** | **Participants (%)** | **Number of events** | **Participants (%)** |
| Blood and lymphatic system disorders | Anaemia | 1 | 1 (2.0%) | 1 | 1 (2.0%) |
|  | Lymphadenopathy | 1 | 1 (2.0%) | 0 | 0 (0%) |
| Ear and labyrinth disorders | Ear pain | 0 | 0 (0%) | 4 | 3 (5.9%) |
|  | Hypoacusis | 0 | 0 (0%) | 1 | 1 (2.0%) |
| Eye disorders | Eye discharge | 2 | 1 (2.0%) | 0 | 0 (0%) |
|  | Vision blurred | 2 | 1 (2.0%) | 0 | 0 (0%) |
|  | Eye irritation | 1 | 1 (2.0%) | 0 | 0 (0%) |
|  | Eye swelling | 1 | 1 (2.0%) | 0 | 0 (0%) |
|  | Eyelid oedema | 1 | 1 (2.0%) | 0 | 0 (0%) |
|  | Eyelid pain | 0 | 0 (0%) | 1 | 1 (2.0%) |
|  | Ocular hyperaemia | 1 | 1 (2.0%) | 0 | 0 (0%) |
| Gastrointestinal disorders | Nausea | 12 | 9 (17.6%) | 35 | 22 (43.1%) |
|  | Abdominal pain upper | 18 | 9 (17.6%) | 11 | 3 (5.9%) |
|  | Abdominal pain | 10 | 7 (13.7%) | 14 | 2 (3.9%) |
|  | Vomiting | 13 | 9 (17.6%) | 11 | 9 (17.6%) |
|  | Diarrhoea | 10 | 8 (15.7%) | 8 | 7 (13.7%) |
|  | Mouth ulceration | 0 | 0 (0%) | 12 | 6 (11.8%) |
|  | Abdominal distension | 2 | 1 (2.0%) | 1 | 1 (2.0%) |
|  | Dyspepsia | 1 | 1 (2.0%) | 1 | 1 (2.0%) |
|  | Faeces discoloured | 1 | 1 (2.0%) | 1 | 1 (2.0%) |
|  | Lip swelling | 2 | 2 (3.9%) | 0 | 0 (0%) |
|  | Abdominal discomfort | 1 | 1 (2.0%) | 0 | 0 (0%) |
|  | Abdominal pain lower | 0 | 0 (0%) | 1 | 1 (2.0%) |
|  | Aphthous ulcer | 1 | 1 (2.0%) | 0 | 0 (0%) |
|  | Constipation | 0 | 0 (0%) | 1 | 1 (2.0%) |
|  | Duodenogastric reflux | 0 | 0 (0%) | 1 | 1 (2.0%) |
|  | Frequent bowel movements | 0 | 0 (0%) | 1 | 1 (2.0%) |
|  | Gingival pain | 1 | 1 (2.0%) | 0 | 0 (0%) |
|  | Lip pain | 1 | 1 (2.0%) | 0 | 0 (0%) |
|  | Retching | 1 | 1 (2.0%) | 0 | 0 (0%) |
| General disorders and administration site conditions | Fatigue | 4 | 3 (5.9%) | 35 | 12 (23.5%) |
|  | Pyrexia | 6 | 3 (5.9%) | 7 | 5 (9.8%) |
|  | Feeling cold | 0 | 0 (0%) | 3 | 1 (2.0%) |
|  | Chest pain | 1 | 1 (2.0%) | 1 | 1 (2.0%) |
|  | Influenza like illness | 2 | 2 (3.9%) | 0 | 0 (0%) |
|  | Malaise | 2 | 1 (2.0%) | 0 | 0 (0%) |
|  | Swelling | 2 | 1 (2.0%) | 0 | 0 (0%) |
|  | Chest discomfort | 0 | 0 (0%) | 1 | 1 (2.0%) |
|  | Feeling hot | 1 | 1 (2.0%) | 0 | 0 (0%) |
|  | Pain | 1 | 1 (2.0%) | 0 | 0 (0%) |
|  | Pallor | 1 | 1 (2.0%) | 0 | 0 (0%) |
|  | Peripheral swelling | 1 | 1 (2.0%) | 0 | 0 (0%) |
| Immune system disorders | Hypersensitivity | 2 | 2 (3.9%) | 0 | 0 (0%) |
|  | Seasonal allergy (hayfever) | 0 | 0 (0%) | 2 | 2 (3.9%) |
|  | Food allergy | 1 | 1 (2.0%) | 0 | 0 (0%) |
| Infections and infestations | Nasopharyngitis | 8 | 7 (13.7%) | 9 | 9 (17.6%) |
|  | Eczema infected | 8 | 6 (11.8%) | 8 | 6 (11.8%) |
|  | Lower respiratory tract infection | 5 | 5 (9.8%) | 3 | 3 (5.9%) |
|  | Upper respiratory tract infection | 4 | 4 (7.8%) | 3 | 3 (5.9%) |
|  | Molluscum contagiosum | 3 | 3 (5.9%) | 3 | 3 (5.9%) |
|  | Viral infection | 0 | 0 (0%) | 6 | 6 (11.8%) |
|  | Folliculitis | 4 | 2 (3.9%) | 1 | 1 (2.0%) |
|  | Skin infection | 2 | 2 (3.9%) | 3 | 3 (5.9%) |
|  | Tonsillitis | 4 | 3 (5.9%) | 1 | 1 (2.0%) |
|  | Herpes simplex | 3 | 2 (3.9%) | 1 | 1 (2.0%) |
|  | Oral herpes | 2 | 1 (2.0%) | 2 | 2 (3.9%) |
|  | Staphylococcal infection | 3 | 3 (5.9%) | 1 | 1 (2.0%) |
|  | Ear infection | 1 | 1 (2.0%) | 2 | 2 (3.9%) |
|  | Gastroenteritis | 2 | 2 (3.9%) | 1 | 1 (2.0%) |
|  | Herpes zoster | 2 | 2 (3.9%) | 1 | 1 (2.0%) |
|  | Rhinitis | 2 | 2 (3.9%) | 1 | 1 (2.0%) |
|  | Urinary tract infection | 1 | 1 (2.0%) | 2 | 1 (2.0%) |
|  | Conjunctivitis | 2 | 1 (2.0%) | 0 | 0 (0%) |
|  | Localised infection | 2 | 2 (3.9%) | 0 | 0 (0%) |
|  | Pharyngitis | 2 | 2 (3.9%) | 0 | 0 (0%) |
|  | Sinusitis | 1 | 1 (2.0%) | 1 | 1 (2.0%) |
|  | Varicella | 0 | 0 (0%) | 2 | 2 (3.9%) |
|  | Viral upper respiratory tract infection | 2 | 1 (2.0%) | 0 | 0 (0%) |
|  | Body tinea | 0 | 0 (0%) | 1 | 1 (2.0%) |
|  | Campylobacter infection | 1 | 1 (2.0%) | 0 | 0 (0%) |
|  | Croup infectious | 1 | 1 (2.0%) | 0 | 0 (0%) |
|  | Eczema herpeticum | 1 | 1 (2.0%) | 0 | 0 (0%) |
|  | Erythema infectiosum | 0 | 0 (0%) | 1 | 1 (2.0%) |
|  | Furuncle | 1 | 1 (2.0%) | 0 | 0 (0%) |
|  | Gingivitis | 0 | 0 (0%) | 1 | 1 (2.0%) |
|  | Helicobacter infection | 1 | 1 (2.0%) | 0 | 0 (0%) |
|  | Infected bite | 1 | 1 (2.0%) | 0 | 0 (0%) |
|  | Lice infestation | 0 | 0 (0%) | 1 | 1 (2.0%) |
|  | Otitis media | 0 | 0 (0%) | 1 | 1 (2.0%) |
|  | Rash pustular | 1 | 1 (2.0%) | 0 | 0 (0%) |
|  | Respiratory tract infection viral | 1 | 1 (2.0%) | 0 | 0 (0%) |
|  | Staphylococcal skin infection | 0 | 0 (0%) | 1 | 1 (2.0%) |
|  | Varicella zoster virus infection | 0 | 0 (0%) | 1 | 1 (2.0%) |
|  | Viral rash | 1 | 1 (2.0%) | 0 | 0 (0%) |
|  | Vulvitis | 1 | 1 (2.0%) | 0 | 0 (0%) |
| Injury, poisoning, and procedural complications | Contusion | 1 | 1 (2.0%) | 0 | 0 (0%) |
|  | Foot fracture | 0 | 0 (0%) | 1 | 1 (2.0%) |
|  | Immunisation anxiety related reaction | 1 | 1 (2.0%) | 0 | 0 (0%) |
|  | Sunburn | 0 | 0 (0%) | 1 | 1 (2.0%) |
| Investigations | Abnormal (decrease of >20% from baseline) estimated glomerular filtration rate | 17 | 14 (27.5%) | 14 | 8 (15.7%) |
|  | Neutrophil count decreased | 3 | 3 (5.9%) | 7 | 2 (3.9%) |
|  | Alanine aminotransferase increased | 1 | 1 (2.0%) | 5 | 3 (5.9%) |
|  | Serum creatinine increased | 2 | 2 (3.9%) | 4 | 2 (3.9%) |
|  | Lymphocyte count decreased | 3 | 3 (5.9%) | 3 | 2 (3.9%) |
|  | Liver function test abnormal | 0 | 0 (0%) | 4 | 1 (2.0%) |
|  | Haemoglobin decreased | 0 | 0 (0%) | 3 | 3 (5.9%) |
|  | Weight decreased | 1 | 1 (2.0%) | 2 | 2 (3.9%) |
|  | Haematocrit decreased | 0 | 0 (0%) | 2 | 1 (2.0%) |
|  | Aspartate aminotransferase abnormal | 1 | 1 (2.0%) | 0 | 0 (0%) |
|  | Aspartate aminotransferase increased | 0 | 0 (0%) | 1 | 1 (2.0%) |
|  | Serum alkaline phosphatase abnormal | 1 | 1 (2.0%) | 0 | 0 (0%) |
|  | Serum alkaline phosphatase decreased | 0 | 0 (0%) | 1 | 1 (2.0%) |
|  | Serum creatinine decreased | 1 | 1 (2.0%) | 0 | 0 (0%) |
|  | Serum potassium increased | 0 | 0 (0%) | 1 | 1 (2.0%) |
|  | Blood pressure increased | 1 | 1 (2.0%) | 0 | 0 (0%) |
|  | Serum sodium increased | 0 | 0 (0%) | 1 | 1 (2.0%) |
|  | Serum urea increased | 1 | 1 (2.0%) | 0 | 0 (0%) |
|  | Cardiac murmur | 0 | 0 (0%) | 1 | 1 (2.0%) |
|  | Lymphocyte count increased | 0 | 0 (0%) | 1 | 1 (2.0%) |
|  | Mean cell volume abnormal | 0 | 0 (0%) | 1 | 1 (2.0%) |
|  | Platelet count increased | 1 | 1 (2.0%) | 0 | 0 (0%) |
|  | Red blood cell count decreased | 0 | 0 (0%) | 1 | 1 (2.0%) |
|  | White blood cell count decreased | 0 | 0 (0%) | 1 | 1 (2.0%) |
| Metabolism and nutrition disorders | Decreased appetite | 4 | 3 (5.9%) | 11 | 8 (15.7%) |
|  | Iron deficiency | 0 | 0 (0%) | 1 | 1 (2.0%) |
| Musculoskeletal and connective tissue disorders | Muscle spasms | 3 | 3 (5.9%) | 0 | 0 (0%) |
|  | Back pain | 1 | 1 (2.0%) | 1 | 1 (2.0%) |
|  | Pain in extremity | 1 | 1 (2.0%) | 1 | 1 (2.0%) |
|  | Arthralgia | 0 | 0 (0%) | 1 | 1 (2.0%) |
|  | Flank pain | 0 | 0 (0%) | 1 | 1 (2.0%) |
|  | Joint swelling | 1 | 1 (2.0%) | 0 | 0 (0%) |
|  | Myalgia | 0 | 0 (0%) | 1 | 1 (2.0%) |
|  | Neck pain | 0 | 0 (0%) | 1 | 1 (2.0%) |
| Nervous system disorders | Headache | 24 | 14 (27.5%) | 27 | 11 (21.6%) |
|  | Dizziness | 5 | 4 (7.8%) | 3 | 3 (5.9%) |
|  | Lethargy | 0 | 0 (0%) | 6 | 3 (5.9%) |
|  | Syncope | 3 | 1 (2.0%) | 0 | 0 (0%) |
|  | Cluster headache | 0 | 0 (0%) | 2 | 1 (2.0%) |
|  | Burning sensation | 1 | 1 (2.0%) | 0 | 0 (0%) |
|  | Hypoaesthesia | 1 | 1 (2.0%) | 0 | 0 (0%) |
|  | Migraine | 1 | 1 (2.0%) | 0 | 0 (0%) |
|  | Paraesthesia | 0 | 0 (0%) | 1 | 1 (2.0%) |
| Psychiatric disorders | Mood swings | 1 | 1 (2.0%) | 13 | 2 (3.9%) |
|  | Mood altered | 3 | 2 (3.9%) | 2 | 1 (2.0%) |
|  | Enuresis | 0 | 0 (0%) | 4 | 1 (2.0%) |
|  | Insomnia | 0 | 0 (0%) | 3 | 2 (3.9%) |
|  | Irritability | 0 | 0 (0%) | 1 | 1 (2.0%) |
|  | Sleep disorder | 1 | 1 (2.0%) | 0 | 0 (0%) |
|  | Stress | 1 | 1 (2.0%) | 0 | 0 (0%) |
| Renal and urinary disorders | Micturition urgency | 1 | 1 (2.0%) | 0 | 0 (0%) |
|  | Polyuria | 1 | 1 (2.0%) | 0 | 0 (0%) |
| Reproductive system and breast disorders | Menorrhagia | 3 | 2 (3.9%) | 0 | 0 (0%) |
|  | Menstruation irregular | 2 | 1 (2.0%) | 0 | 0 (0%) |
|  | Balanoposthitis | 0 | 0 (0%) | 1 | 1 (2.0%) |
|  | Dysmenorrhoea | 1 | 1 (2.0%) | 0 | 0 (0%) |
|  | Menstruation delayed | 0 | 0 (0%) | 1 | 1 (2.0%) |
|  | Testicular swelling | 1 | 1 (2.0%) | 0 | 0 (0%) |
|  | Vulvovaginal pruritus | 0 | 0 (0%) | 1 | 1 (2.0%) |
| Respiratory, thoracic, and mediastinal disorders | Cough | 6 | 5 (9.8%) | 7 | 6 (11.8%) |
|  | Oropharyngeal pain | 6 | 4 (7.8%) | 6 | 4 (7.8%) |
|  | Rhinorrhoea | 3 | 2 (3.9%) | 2 | 2 (3.9%) |
|  | Wheezing | 0 | 0 (0%) | 5 | 4 (7.8%) |
|  | Asthma | 1 | 1 (2.0%) | 3 | 3 (5.9%) |
|  | Epistaxis | 2 | 2 (3.9%) | 1 | 1 (2.0%) |
|  | Productive cough | 0 | 0 (0%) | 2 | 2 (3.9%) |
|  | Dyspnoea | 0 | 0 (0%) | 1 | 1 (2.0%) |
|  | Nasal congestion | 1 | 1 (2.0%) | 0 | 0 (0%) |
| Skin and subcutaneous tissue disorders | Eczema flare | 45 | 22 (43.1%) | 19 | 15 (29.4%) |
|  | Pruritus | 4 | 3 (5.9%) | 4 | 2 (3.9%) |
|  | Rash | 4 | 4 (7.8%) | 2 | 2 (3.9%) |
|  | Alopecia | 4 | 4 (7.8%) | 1 | 1 (2.0%) |
|  | Acne | 3 | 3 (5.9%) | 0 | 0 (0%) |
|  | Urticaria | 3 | 3 (5.9%) | 0 | 0 (0%) |
|  | Blister | 2 | 2 (3.9%) | 0 | 0 (0%) |
|  | Dermatitis atopic | 2 | 2 (3.9%) | 0 | 0 (0%) |
|  | Dry skin | 0 | 0 (0%) | 2 | 2 (3.9%) |
|  | Dyshidrotic eczema | 0 | 0 (0%) | 1 | 1 (2.0%) |
|  | Erythema | 0 | 0 (0%) | 1 | 1 (2.0%) |
|  | Hirsutism | 1 | 1 (2.0%) | 0 | 0 (0%) |
|  | Hypertrichosis | 1 | 1 (2.0%) | 0 | 0 (0%) |
|  | Impetigo | 1 | 1 (2.0%) | 0 | 0 (0%) |
|  | Ingrowing nail | 1 | 1 (2.0%) | 0 | 0 (0%) |
|  | Miliaria | 1 | 1 (2.0%) | 0 | 0 (0%) |
|  | Night sweats | 1 | 1 (2.0%) | 0 | 0 (0%) |
|  | Skin reaction | 1 | 1 (2.0%) | 0 | 0 (0%) |
| **Total** | | **369** | **48 (94.1%)** | **407** | **47 (92.2%)** |

Supplementary Table 12: Participants using anti-inflammatory concomitant medications during and after the treatment phase

| **Time point** | **Topical corticosteroids [TC]** | | **Calcineurin inhibitors [C]** | | **Topical anti-inflammatory** | |
| --- | --- | --- | --- | --- | --- | --- |
|  | **CyA**  **N (% of 52)** | **MTX**  **N (% of 51)** | **CyA**  **N (% of 52)** | **MTX**  **N (% of 51)** | **CyA**  **N (% of 52)** | **MTX**  **N (% of 51)** |
| Whilst on randomised systemic therapy | 43 (82.7%) | 36 (70.6%) | 17 (32.7%) | 16 (30.8%) | 44 (84.6%) | 36 (70.6%) |
| Off randomised systemic therapy | 40 (76.9%) | 31 (60.8%) | 23 (44.2%) | 13 (25.5%) | 43 (82.7%) | 33 (64.7%) |

Supplementary Figure 1. Kaplan-Meier curve comparing the time to significant re-flares of disease in the two treatment arms (CyA (blue) vs MTX (red)).

*
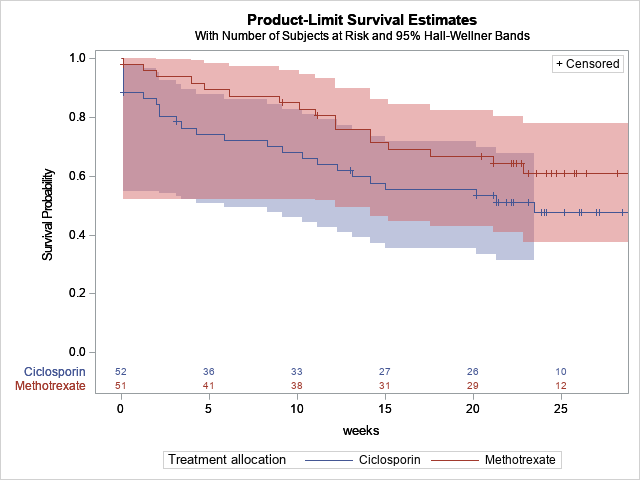
*

Supplementary Figure 2: Mean profile plots for IGA over time^*^


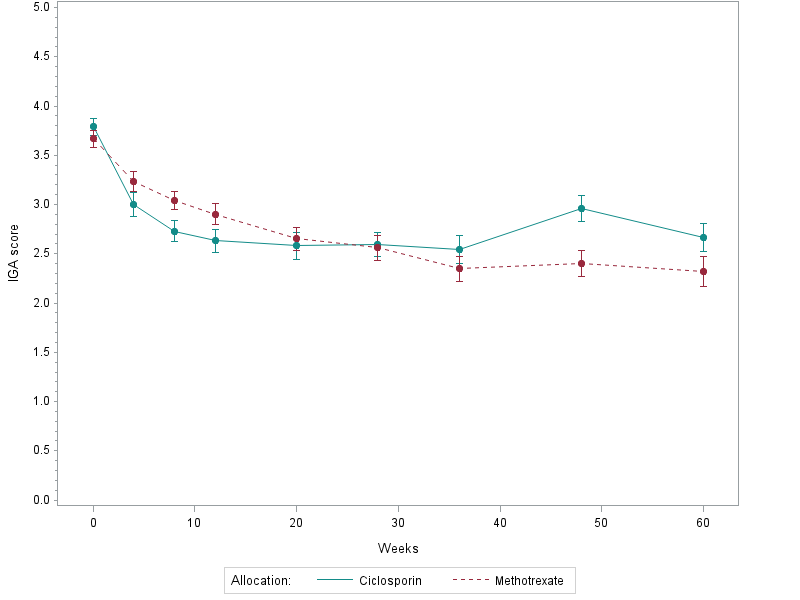


* Raw mean estimates with standard error bars

Supplementary Figure 3: Mean profile plots for POEM over time^*^


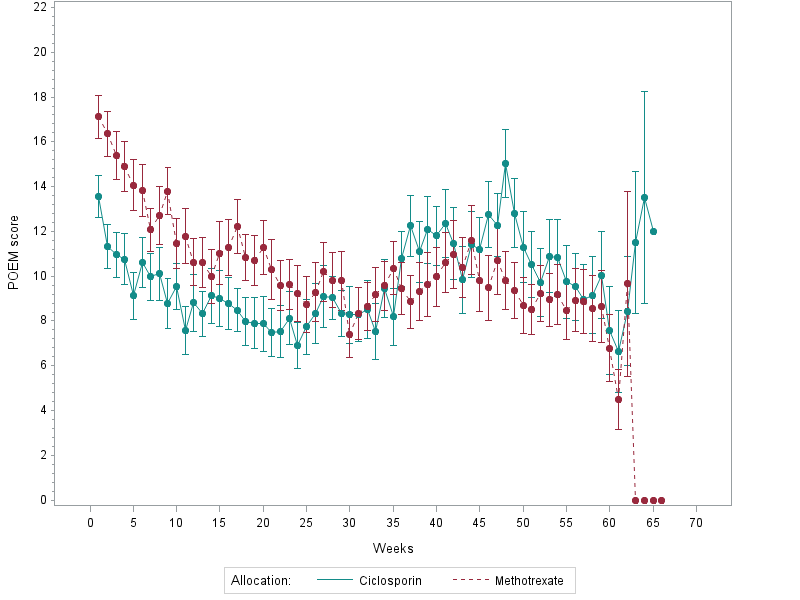


* Raw mean estimates with standard error bars (data are presented after 60 weeks due to participant completion of diaries)

Figure 4. Mean profile plot of CDLQI over time^*^

*
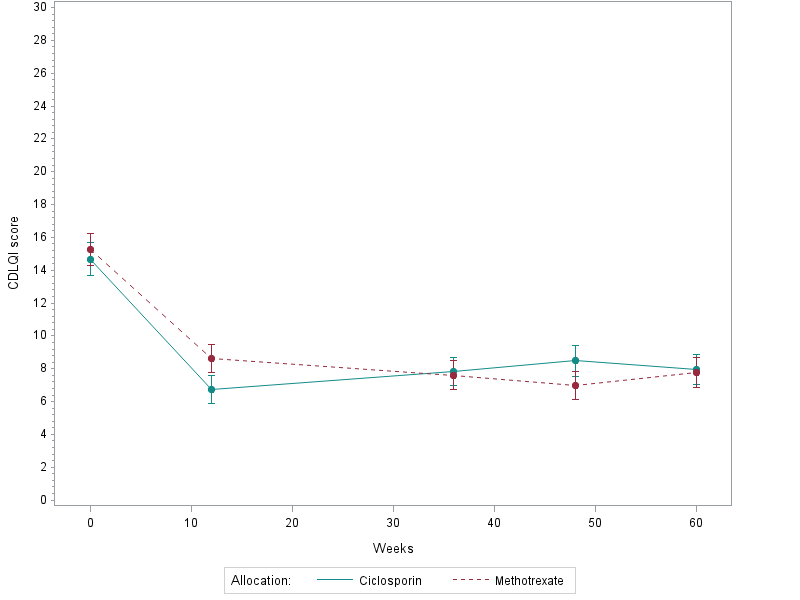
*

* Raw mean estimates with standard error bars

Figure 5. Mean profile plot of IDQoL over time^*^

*
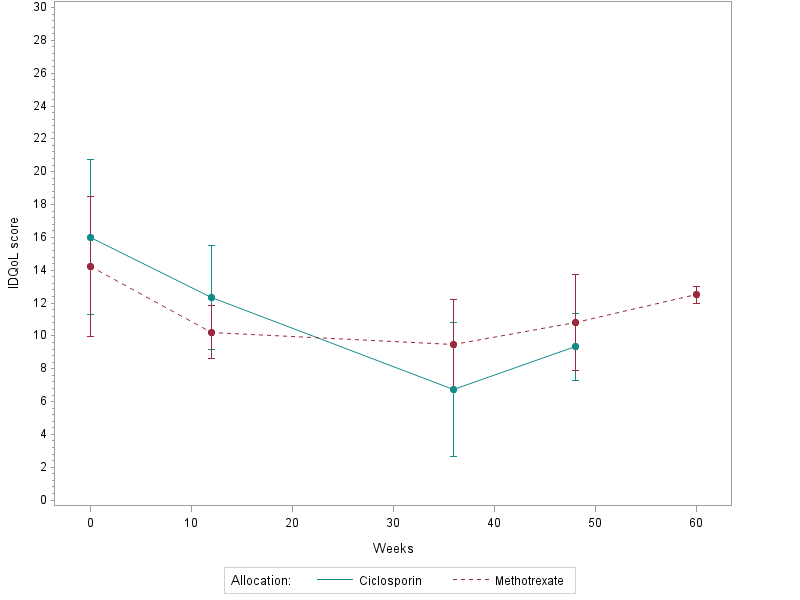
*

* Raw mean estimates with standard error bars

Figure 6. Mean profile plot of DFI over time^*^

*
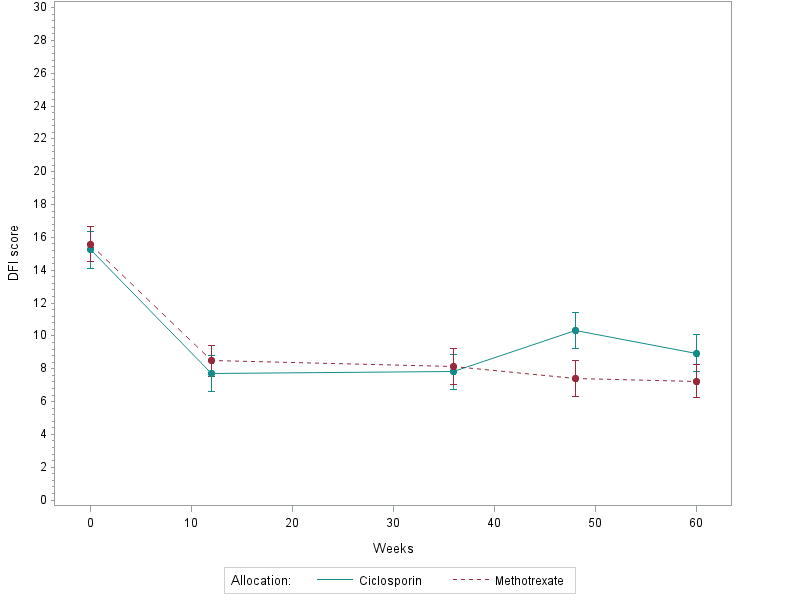
*

* Raw mean estimates with standard error bars


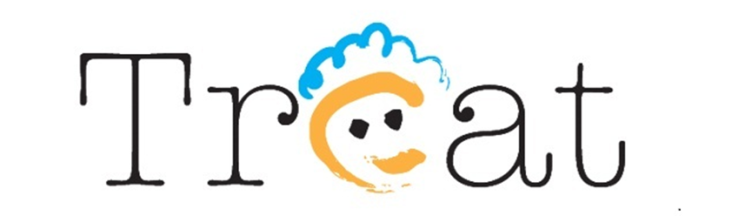


**TREAT**

**Eudract No.: 2015-002013-29**

**ISRCTN number: 15837754**

**Statistical Analysis Plan v2.0**

|  | **ORIGINATED BY** | **QC PERFORMED BY** | **APPROVED BY** |
| --- | --- | --- | --- |
| **Name** | Anna Rosala-Hallas | Primary Outcome: Dannii Clayton  Safety: Ashley Best | Ashley Jones |
| **Title** | Trial Statistician | QC Statistician | Lead Statistician |
| **Date** | 14/04/2021 | | |
| **Protocol Version and Date** | Version 4.0 17/10/2016 | | |

# Change Control

| **Protocol version** | **Updated SAP version no.** | **Section number changed** | **Description of change** | **Date changed** |
| --- | --- | --- | --- | --- |
| 7.0 | 2.0 | 3 | Changes made to affiliations (Department of Biostatistics changed to LCTC). Addition of R. Jackson as blind reviewer. | 26/03/21 |
| 7.0 | 2.0 | 5 | Protocol version updated to Version 7.0 29/07/2020.  ‘Clinical Trials Research Centre’ changed to ‘Liverpool Clinical Trials Centre’. | 26/03/21 |
| 7.0 | 2.0 | 14.1, 17.2, 17.3 | References to ‘closed section of the report’ removed. | 26/03/21 |
| 7.0 | 2.0 | 17.4.2.1 | Time to first significant flare definition changed to include re-starting a systemic treatment as per protocol change. | 26/03/21 |
| 7.0 | 2.0 | 17.4.2.1 | **Change suggested by blind reviewer:**  *Time to first flare will be calculated from date of end of treatment to 24 weeks (+2 week window) following treatment cessation.*  Changed to:  *Time to first flare will be calculated from date of end of treatment to last trial visit following treatment cessation.* | 26/03/21 |
| 7.0 | 2.0 | 17.4.2.2 | **Change suggested by blind reviewer:**  *Time to first flare will be calculated from date of end of treatment to 24 weeks (+2 week window) following treatment cessation.*  Changed to:  *Time to first flare will be calculated from date of end of treatment to last trial visit following treatment cessation.*  A continuity correction of 1 day will be added to all censor/event times in order to include participants who failed or were censored at time=0.  Correction to wording which suggests survival times are incorrectly calculated from randomisation rather than end of treatment as defined earlier in section. | 26/03/21 |
| 7.0 | 2.0 | 17.4.4.1 | Re-flare definition changed to include re-starting a systemic treatment as per protocol change. | 26/03/21 |
| 7.0 | 2.0 | 17.4.3.2 | **Change suggested by blind reviewer:**  IGA will be analysed using ordinal longitudinal regression rather than a linear mixed model. | 26/03/21 |
| 7.0 | 2.0 | 17.4.4.2 | Aspects of outcome related to Health Economics analysis removed. | 26/03/21 |
| 7.0 | 2.0 | 17.4.4.2 | **Change suggested by blind reviewer:**  Analyses will be restricted to those who completed more than 80% of the diaries. Patterns of missingness will be explored. | 26/03/21 |
| 7.0 | 2.0 | 17.4.7.2 | **Change suggested by blind reviewer:**  IDQoL will be summarised descriptively. | 26/03/21 |
| 7.0 | 2.0 | 17.4.9 | Addition of analysis and derivation of samples outcomes. These analyses were not specified in SAP Version 1.0 but have been added to this version of the SAP prior to receipt of the data. | 26/03/21 |

# Approval and agreement

**SAP Version Number being approved: 2.0**

**Trial Statistician**

Name Anna Rosala-Hallas

Signed Date

**Senior Statistician**

Name Ashley Jones

Signed Date

**Chief Investigator/clinical lead**

Name Carsten Flohr

Signed Date

|  |
| --- |

# Roles and responsibilities

A. Rosala-Hallas (LCTC, University of Liverpool), A. Jones (LCTC, University of Liverpool), C. Flohr (Department of Paediatric Dermatology, King’s College London). R. Jackson (LCTC, University of Liverpool).

**Author’s contributions**

A. Rosala-Hallas and A. Jones proposed the statistical analysis plan. A. Rosala-Hallas drafted the manuscript. A. Jones, C. Flohr read, amended and approved the statistical analysis plan. R. Jackson reviewed the Blind Review document and suggested changes to the original plan, documented within Section 1. Change Control.

# List of abbreviations and definitions of terms

| AE | Adverse Event |
| --- | --- |
| CI | Chief Investigator |
| CONSORT | Consolidated Standards of Reporting Trials |
| CRF | Case Report Form |
| CTRC | Clinical Trials Research Centre |
| CTU | Clinical Trials Unit |
| CyA | Ciclosporin |
| EASI | Eczema Area and Severity Index |
| EME | Efficacy and Mechanism Evaluation programme |
| ICH | International Conference of Harmonisation |
| IDSMC | Independent Data and Safety and Monitoring Committee |
| IGA | Investigator Global Assessment |
| IMP | Investigational Medicinal Product |
| IQR | Inter-quartile range |
| MedDRA | Medical Dictionary for Regulatory Activities |
| MTX | Methotrexate |
| o-SCORAD | Objective Scoring Atopic Dermatitis |
| SAP | Statistical Analysis Plan |
| SAE | Serious adverse event |
| SD | Standard deviation |
| SOP | Standard operating procedure |
| TSC | Trial Steering Committee |

Contents

[1. Change Control 2](#_Toc69297574)

[2. Approval and agreement 3](#_Toc69297575)

[3. Roles and responsibilities 4](#_Toc69297576)

[4. List of abbreviations and definitions of terms 5](#_Toc69297577)

[5. Statement of Compliance 9](#_Toc69297578)

[6. Background and Rationale 9](#_Toc69297579)

[7. TREAT Study Objectives 9](#_Toc69297580)

[8. Investigational Plan and Study Design 10](#_Toc69297581)

[8.1. Overall study design and plan- description 10](#_Toc69297582)

[8.2. Treatments studied 10](#_Toc69297583)

[8.3. Treatment compliance 10](#_Toc69297584)

[8.4. Patient population studied 10](#_Toc69297585)

[8.5. Inclusion criteria 10](#_Toc69297586)

[8.6. Exclusion criteria 10](#_Toc69297587)

[8.7. Removal of patients from therapy or assessment 11](#_Toc69297588)

[8.8. Consent process 11](#_Toc69297589)

[8.9. Blinding 11](#_Toc69297590)

[8.10. Method of assignment to treatment 11](#_Toc69297591)

[8.11. Sequence and duration of all study periods 11](#_Toc69297592)

[8.12. Schedule of assessments 11](#_Toc69297593)

[9. Listing of Outcomes 13](#_Toc69297594)

[10. Determination of Sample Size 13](#_Toc69297595)

[11. Study Framework 13](#_Toc69297596)

[12. Confidence Intervals, p-values and Multiplicity 13](#_Toc69297597)

[13. Timing and Objectives of Interim and Final Analyses 14](#_Toc69297598)

[13.1. Interim monitoring and analyses 14](#_Toc69297599)

[13.2. Final analysis 14](#_Toc69297600)

[14. Disposition of Participants 14](#_Toc69297601)

[14.1. Screening, eligibility and recruitment 14](#_Toc69297602)

[14.2. Post randomisation discontinuations 16](#_Toc69297603)

[15. Protocol Deviations 16](#_Toc69297604)

[16. Unblinding 16](#_Toc69297605)

[17. Efficacy Evaluations 17](#_Toc69297606)

[17.1. Data Sets Analysed 17](#_Toc69297607)

[17.2. Demographic and Other Baseline Characteristics 17](#_Toc69297608)

[17.3. Compliance with treatment 18](#_Toc69297609)

[17.4. Analysis of outcomes 18](#_Toc69297610)

[17.4.1. Co-primary outcome i) Change in atopic eczema severity between baseline and 12 weeks of treatment, using the o-SCORAD index 18](#_Toc69297611)

[17.4.1.1. Derivation 18](#_Toc69297612)

[17.4.1.2. Analysis 18](#_Toc69297613)

[17.4.2. Co-primary outcome ii) Time to first significant flare (returning to baseline o-SCORAD) during the 24 weeks after treatment cessation in the MTX vs CyA groups 19](#_Toc69297614)

[17.4.2.1. Derivation 19](#_Toc69297615)

[17.4.2.2. Analysis 20](#_Toc69297616)

[17.4.3. Change in atopic eczema severity using the EASI, IGA, o-SCORAD and POEM between baseline and 12, 36, 48, and 60 weeks 21](#_Toc69297617)

[17.4.3.1. Derivation 21](#_Toc69297618)

[17.4.3.2. Analysis 22](#_Toc69297619)

[17.4.4. Number of parent-reported flares in each study arm as well as the proportion of children who re-flared during the 24 weeks after treatment cessation 22](#_Toc69297620)

[17.4.4.1. Derivation 22](#_Toc69297621)

[17.4.4.2. Analysis 22](#_Toc69297622)

[17.4.5. Proportion of participants achieving 50% improvement in the o-SCORAD index at 12, 36, 48, and 60 weeks 23](#_Toc69297623)

[17.4.5.1. Derivation 23](#_Toc69297624)

[17.4.5.2. Analysis 23](#_Toc69297625)

[17.4.6. Proportion of participants who withdraw from treatment because of AEs 24](#_Toc69297626)

[17.4.6.1. Derivation 24](#_Toc69297627)

[17.4.6.2. Analysis 24](#_Toc69297628)

[17.4.7. Disease-specific patient and parental quality of life (QoL) measured with the CDLQI/IDQOL and DFI scores between baseline and 12, 36, 48 & 60 weeks 24](#_Toc69297629)

[17.4.7.1. Derivation 24](#_Toc69297630)

[17.4.7.2. Analysis 27](#_Toc69297631)

[17.4.8. Assess the cost-effectiveness of CyA vs MTX, using a cost/QALY analysis 27](#_Toc69297632)

[17.4.9. Immuno-metabolic effects of MTX and CyA, especially in relation to markers of glycolytic activation and T cell cytokine signature, at baseline, during treatment and up to 24 weeks after completion of treatment. 27](#_Toc69297633)

[17.4.10. Drug-related side effects of both MTX and CyA and their association with MTX polyglutamate and CyA trough levels. 28](#_Toc69297634)

[17.4.10.1. Derivation 28](#_Toc69297635)

[17.4.10.2. Analysis 28](#_Toc69297636)

[17.4.11. The association between MTX polyglutamate and CyA trough levels and treatment response (reduction in disease severity) 28](#_Toc69297637)

[17.4.11.1. Derivation 28](#_Toc69297638)

[17.4.11.2. Analysis 28](#_Toc69297639)

[17.4.12. The association between FLG carriage (yes/no) and treatment response 28](#_Toc69297640)

[17.4.12.1. Derivation 28](#_Toc69297641)

[17.4.12.2. Analysis 29](#_Toc69297642)

[18. Missing data and withdrawals 29](#_Toc69297643)

[19. Additional analyses 29](#_Toc69297644)

[20. Safety Evaluations 29](#_Toc69297645)

[20.1. Data sets analysed 29](#_Toc69297646)

[20.2. Presentation of the data 29](#_Toc69297647)

[21. Quality Control 30](#_Toc69297648)

[22. References 31](#_Toc69297649)

# Statement of Compliance

This Statistical Analysis Plan (SAP) provides a detailed and comprehensive description of the pre-planned final analyses for the study “TREAT” to be performed by the trial statistician. The planned statistical analyses described within this document are compliant with those specified in brief within the TREAT protocol version 7.0 29/07/2020.

This study is carried out in accordance with the World Medical Association Declaration of Helsinki (1964) and the Tokyo (1975), Venice (1983), Hong Kong (1989) and South Africa (1996) amendments and will be conducted in compliance with the protocol, Clinical Trials Research Centre (CTRC) Clinical Trials Unit (CTU) Standard Operating Procedures (SOPs) and EU Directive 2001/20/EC, transposed into UK law as the UK Statutory Instrument 2004 No 1031: Medicines for Human Use (Clinical Trials) Regulations 2004.

This study is a clinical trial of a medicinal product and is registered on the EudraCT database. The statistical analysis plan has been developed to support the posting of results on the EudraCT system. This is a regulatory requirement which should be fulfilled within 6 months after the end of the study as defined within the clinical trial protocol.

The results of the final analysis described within this statistical analysis plan will be contained within a statistical analysis report. This report will be used as the basis of the primary research publications according to the study publication plan.

All analyses are performed with standard statistical software (SAS version 9.3 or later). The finalised analysis datasets, programs and outputs will be archived following Good Clinical Practice guidelines and SOP TM021 Archiving procedure in CTRC. The testing and validation of the statistical analysis programs will be performed following SOP ST001.

# Background and Rationale

A full background can be found in section 2 of the protocol. To summarise, atopic eczema is a chronic, pruritic inflammatory skin disease affecting around 20% of UK children. Current treatment options are limited with most physicians in Europe choosing Ciclosporin (CyA) to treat their patients, however there is no clear consensus on which treatment is the most appropriate. Recently there has been interest in the use of methotrexate (MTX) to treat this condition which prompts the need for a randomised controlled trial.

# TREAT Study Objectives

The primary and secondary objectives of the trial can be found listed in section 1 of the protocol.

The first null hypothesis is that there is no difference in change in atopic eczema severity between baseline and 12 weeks of treatment between the MTX and CyA treated arms.

The second null hypothesis is that there is no difference in time to first flare during the 24 weeks after treatment cessation.

The alternate hypotheses are that there is a difference between the two groups.

# Investigational Plan and Study Design

### Overall study design and plan- description

TREAT is a Phase III, multi-centre, randomised, superiority, observer-blind trial assessing the safety and cost-effectiveness of MTX versus CyA in the treatment of severe atopic eczema in children.

### Treatments studied

Section 7 of the protocol details the treatments studied. To summarise, those assigned to MTX will receive an initial test dose of 0.1mg/kg/week then 0.4mg/kg/week. Those assigned to CyA will receive 4mg/kg/day in two divided doses for 36 weeks. Participants should remain on full treatment dose for the 8 weeks. After that, dose increases to a maximum of 5mg/kg/day or dose decreases are allowed according to treatment response.

### Treatment compliance

Participants are advised to return any unused IMPs. Accountability logs will be used to record IMP administered and returned unused. Participant diaries also record the number of missed doses with reasons.

### Patient population studied

The participant population to be studied will include 102 children aged 2-16 years with severe atopic eczema and inadequately responding to moderate (face) and potent (body) topical treatment.

### Inclusion criteria

The inclusion criteria can be found in section 5.1 of the protocol.

### Exclusion criteria

The exclusion criteria can be found in section 5.2 of the protocol.

### Removal of patients from therapy or assessment

If a participant’s safety data are not obtained for at least one of the treatment visits they may be removed from trial treatment, but continue with follow-up in the trial. Any medication they take from point of discontinuation will be recorded in the CRF *Form 13: Concomitant Medication*.

### Consent process

Full details on the consent procedure can be found in section 11.3 of the protocol. Consent is prospective and will take place before any trial specific tests or visits.

### Blinding

Due to the nature of the treatments it is not possible to blind the participants, research staff or local investigator. However, those who will perform the severity assessments will be blinded to treatment allocation. Section 6.4.2 of the protocol gives full details on the blinding procedure.

### Method of assignment to treatment

Participants will be randomised in a 1:1 ratio using a web-based randomisation system. Information on stratification factors can be found in ST002TEM01 Randomisation Specification, filed in section 2 of the Statistics Trial File.

### Sequence and duration of all study periods

A study flow chart can be found in section 1 of the protocol. To summarise, patients with severe atopic eczema are screened for eligibility. After consent for the trial is sought from eligible participants they move onto the screening visit and are screened for eligibility to continue to the baseline visit. Participants eligible at the screening visit then move onto the baseline visit and are screened again for eligibility to be randomised. Those eligible are subsequently randomised 1:1 to either MTX or CyA.

### Schedule of assessments

A full schedule of assessments can be found in section 8 of the protocol. Table 8.12.1 is a condensed version detailing the times of assessments for the primary and secondary outcomes.

Table 8.12‑1: schedule of assessments

|  |  | **Week 0** | **Week 1 *** | **Week 2** | **Week 4** | **Week 8** | **Week 12** | **Week 20** | **Week 28** | **Week 36** | **Week 48** | **Week 60** |
| --- | --- | --- | --- | --- | --- | --- | --- | --- | --- | --- | --- | --- |
| **Procedures** | Screening Visit | Baseline/  Rand | Visit 1 | Visit 2 | Visit 3 | Visit 4 | Visit 5 | Visit 6 | Visit 7 | Visit 8 | Visit 9 | Visit 10 |
| o-SCORAD | **X** | **X** |  |  | **X** | **X** | **X** | **X** | **X** | **X** | **X** | **X** |
| EASI, IGA |  | **X** |  |  | **X** | **X** | **X** | **X** | **X** | **X** | **X** | **X** |
| POEM |  | **X** |  |  | **X** | **X** | **X** | **X** | **X** | **X** | **X** |  |
| Parent and child QoL |  | **X** |  |  | **X** | **X** | **X** |  |  | **X** | **X** | **X** |
| Adverse events |  |  | **X** | **X** | **X** | **X** | **X** | **X** | **X** | **X** |  |  |
| Urine sample collection (NAG) |  | **X** |  | **X** |  |  | **X** |  |  | **X** |  | **X** |
| MTX metabolite level |  |  |  | **X** |  | **X** | **X** |  |  | **X** |  |  |
| CyA trough level* |  |  |  | **X** |  | **X** | **X** |  |  | **X** |  |  |
| Cystatin C level |  | **X** |  | **X** |  | **X** | **X** |  |  | **X** |  | **X** |
| Creatinine level |  | **X** |  | **X** |  | **X** | **X** |  |  | **X** |  | **X** |
| Blood/saliva for FLG genotyping |  | **X** |  |  |  |  |  |  |  |  |  |  |
| Tape stripping for cutaneous metabolic work |  | **X** |  |  |  |  | **X** |  |  | **X** |  | **X** |
| Collection of blood for mechanistic studies |  | **X** |  |  |  |  | **X** |  |  | **X** |  | **X** |

*MTX arm only.

# Listing of Outcomes

- 1. **Primary outcome(s)**

1. Change in atopic eczema severity between baseline and 12 weeks of treatment, using the o-SCORAD index.
2. Time to first flare (return to baseline o-SCORAD) during the 24 weeks after treatment cessation in the MTX vs CyA groups.
   1. **Secondary outcomes**
3. Change in atopic eczema severity using the EASI, IGA, o-SCORAD and POEM between baseline and 12, 36, 48, and 60 weeks.
4. Number of flares in each study arm as well as the proportion of children who re-flared during the 24 weeks after treatment cessation.
5. Proportion of participants achieving 50% improvement in the o-SCORAD index at 12, 36, 48, and 60 weeks.
6. Proportion of participants who withdraw from treatment because of AEs.
7. Disease-specific patient and parental quality of life (QoL) measured with the CDLQI/IDQOL and DFI scores between baseline and 12, 36, 48 & 60 weeks.
8. Assess the cost-effectiveness of CyA vs MTX, using a cost/QALY analysis.
9. Immuno-metabolic effects of MTX and CyA, especially in relation to markers of glycolytic activation and T cell cytokine signature, at baseline, during treatment and up to 24 weeks after completion of treatment.
10. Drug-related side effects/toxicity profiles of both MTX and CyA and their association with MTX polyglutamate and CyA trough levels.
11. The association between MTX polyglutamate and CyA trough levels and reduction in in AE severity as well as drug-related side effects.
12. The association between FLG carriage (yes/no) and treatment response.

# Determination of Sample Size

TREAT aims to recruit 102 patients. The sample size calculation can be found in section 9.6 of the protocol.

# Study Framework

The overall objectives for each of the study outcomes (primary and secondary) are to test the superiority of MTX compared to CyA.

# Confidence Intervals, p-values and Multiplicity

No formal statistical testing will be undertaken on any of the data summaries presented to the IDSMC unless formal interim analyses of the primary efficacy outcomes are requested. If an interim analysis is requested then the Haybittle-Peto method will be used, which employs 99.9% confidence intervals for the analysis effect estimated, allowing the final analysis to use 95% confidence intervals. This equates to a 0.1% significance level for the interim analysis and 5% for the final analysis.

Due to the fact that there are two primary outcomes (o-SCORAD and flare) the level of significance at the final analysis will be adjusted to account for this multiplicity. The level of significance will be 0.025, accounting for the two primary outcomes. All secondary outcomes will use the usual value of 0.05 to state statistical significance and no adjustments for multiplicity will be made.

# Timing and Objectives of Interim and Final Analyses

### Interim monitoring and analyses

Descriptive analyses of the accumulating data will be performed at regular intervals (at least annually) for review by an IDSMC as stated within section 5.3 of the IDSMC Charter.

After the primary outcome data are available from 25 patients (o-SCORAD index at 12 weeks) the standard deviation of the 25 scores, and the 95% confidence limits for this estimate, will be calculated without unblinding allocation and presented in the open section of the report. If the 95% confidence limits of the estimate of the standard deviation (SD) of the o-SCORAD index at 12 weeks overlap 10 the trial will continue unchanged. If the upper 95% confidence limit of the estimate of the SD of the o-SCORAD index at 12 weeks is less than 10 the trial will continue unchanged but the TSC will be informed that the trial power is greater than planned. If the lower 95% confidence limit of the estimate of the SD is greater than 10 the study is underpowered. The Efficacy and Mechanism Evaluation programme (EME) will decide whether to invite an extension or close the study.

### Final analysis

The final analysis will take place at the end of the trial which is defined in Section 8.6 of the protocol to be the date on which data for all participants has been finalised and entered onto the database with data entry privileges withdrawn (data lock).

# Disposition of Participants

### Screening, eligibility and recruitment

Screening logs will be summarised by site in a table detailing:

1. the number of patients who were approached at screening;
2. those who were eligible at screening (expressed as a frequency and a % with the denominator being i);
3. those who were eligible at screening and provided consent (expressed as a frequency and a % with the denominator being ii);
4. those who proceeded to the baseline visit (expressed as a frequency and a % with the denominator being iii);
5. those who were eligible at baseline for randomisation (expressed as a frequency and a % with the denominator being vi);
6. those who were randomised (expressed as a frequency and a % with the denominator being v);

Reasons for ineligibility at the screening visit and baseline visit will be summarised by site and overall in a table with the following categories for reasons being the inclusion/exclusion criteria. Frequencies will be presented along with percentages using the denominator as i and iv respectively.

Reasons for consent declined will be summarised by site and overall in a table with the following categories:

1. Does not want to consent (unwilling to provide reason).
2. Unwilling/unable to comply with study requirements.
3. Does not wish to consent for another reason (specify).

Frequencies will be presented along with percentages using the denominator as ii).

A recruitment summary table will be presented showing the following for each centre: centre code, hospital name, dates site opened/closed to recruitment, dates of first/last randomisation and total number randomised. Planned total recruitment and numbers recruited to date will also be presented by site. The numbers randomised to each treatment arm by site will be presented.

A recruitment graph showing the expected and actual number of centres to be opened and the expected and actual number of participants randomised per month will be displayed.

A CONSORT flow diagram [9] will be used to summarise the number of patients who were:

- assessed for eligibility at screening
- eligible at screening
- ineligible at screening*
- eligible at screening and consented
- eligible at screening but not consented
- proceeded to baseline visit
- eligible at baseline
- ineligible at baseline*
- randomised
- not randomised*
- received the randomised allocation
- did not receive the randomised allocation*
- lost to follow-up*
- discontinued the intervention*
- randomised and included in the primary analysis
- randomised and excluded from the primary analysis*

*reasons will be provided

### Post randomisation discontinuations

Withdrawals from follow-up will be recorded and presented (see Section 18). The number of participants who prematurely discontinued treatment will be summarised with date of randomisation, date of discontinuation and reason for discontinuation.

# Protocol Deviations

Possible protocol deviations will be defined in GE010TEM01 Monitoring Plan and specified as minor or major.

Protocol deviations will be classified and signed-off using ST001TEM03 Protocol Deviations and Population Exclusions template prior to requesting the treatment allocations and any analysis being performed. The number (and percentage) of patients with at least one major/minor/all protocol deviation will be summarised by site and in total by treatment arm. The patients that are included in the intention to treat (ITT) analysis data set will be used as the denominator to calculate the percentages. No formal statistical testing will be undertaken.

# Unblinding

Participants are not blinded for this study. It will be recorded whether the blinded assessor remained blinded during the assessment and will be presented as a line listing.

# Efficacy Evaluations

### Data Sets Analysed

The principle of intention-to-treat, as far as practically possible, will be the main strategy of the analysis adopted for the primary outcomes and all the secondary outcomes. These analyses will be conducted on all randomised participants, in the group to which they were allocated, and for whom the outcome(s) of interest have been observed/measured. No imputations will be made.

The membership of the analysis set for each outcome will be determined and documented and reasons for participant exclusion will be given prior to the blind being broken and the randomisation lists being requested. Reasons may include missing data, loss to follow up.

Please note: Participants to be excluded from analysis populations need to be defined in template *ST001TEM04: Protocol deviations and data set definitions* and agreed and approved prior to any release of randomisation code.

### Demographic and Other Baseline Characteristics

Descriptive statistics will be reported split by treatment arm. Categorical data will be summarised by numbers and percentages. Continuous data will be summarised by mean, standard deviation (SD), median, inter-quartile range (IQR), minimum and maximum. Variables to be included in the table are as follows:

| **Variable** | **CRF** |
| --- | --- |
| Number randomised | CRF Tracker |
| **Demographic details** | |
| Gender (Male/Female) | Form 1: Screening |
| Age (years) |  |
| Weight (kg) |  |
| BMI |  |
| Ethnicity  (White British; Other White; Black British; Chinese; Mixed; Other (specify) |  |
| **Severity assessments** | |
| IGA | Form 5: IGA |
| EASI | Form 6: EASI |
| o-SCORAD | Form 7: oSCORAD |
| POEM | Form X: POEM |
| **Quality of Life** | |
| CDLQI | Children’s Dermatology Life Quality Index |
| IDQOL | Infant Dermatitis Quality of Life Index (IDQOL) |
| DFI | Dermatitis Family Impact Questionnaire |

### Compliance with treatment

A line listing of the number of expected doses and missed doses per participant will be presented. Reasons for missed doses will be summarised.

### Analysis of outcomes

### Co-primary outcome i) Change in atopic eczema severity between baseline and 12 weeks of treatment, using the o-SCORAD index

### Derivation

The o-SCORAD score can be found on the CRF *Form 7: o-SCORAD.*  The score will be automatically checked against the individual components via a database validation and recorded in ST007TEM01 Data cleaning and complex checks record.

### Analysis

The method of analysis of covariance (ANCOVA) will be used for this outcome. The outcome measure will be o-SCORAD score at 12 weeks post randomisation (+/- 2 weeks) and the explanatory variables will be treatment group and baseline o-SCORAD score. The mean o-SCORAD at baseline (Week 0) and at Week 12 and the mean change from baseline to week 12 (week 12-week 0) will be presented by treatment group. The mean difference in change from baseline between the two groups will be reported with 97.5% confidence intervals. A two sided p-value of 0.025 (2.5% level) will be used to declare statistical significance and will be reported alongside the 97.5% confidence interval.

Although randomisation is stratified by centre, due to the small numbers of expected recruitment in each centre, it will not be included as a covariate in the analyses as this could lead to unreliable estimates and p-values which are either too large or too small.

ICH E9 states that “In some trials there may be no reason to expect the centres to have any influence on the primary or secondary variables because they are unlikely to represent influences of clinical importance. In other trials the limited number of subjects per centre will make it impracticable to include centre effects in the statistical model” (2). Heterogeneity of treatment effects by centre will be considered in a graphical display by means of a Forest plot split by site. A sensitivity analysis including a random effect for site will be undertaken for the co-primary outcomes.

The assumptions underlying the ANCOVA model will be explored. Normality of residuals will be checked by inspection of histograms and Q-Q plots. If residuals do not appear normally distributed the non-parametric Mann-Whitney U-test will be used. The assumption of homoscedasticity will be assessed by inspection of scatter plots of the residuals against the fitted values. Where scatter plots suggest non-constant residual variance, sandwich estimators will be used to estimate standard errors and 95% confidence intervals.

The primary analysis will include only participants who have both a baseline and 12 week o-SCORAD score.

The following sensitivity analyses will be carried out for this outcome:

1. Inclusion of participants with a missing baseline score or 12 weeks score, using last observation carried forward (screening score for baseline).
2. Participants with missing score at 12 weeks imputed using the average score of the remaining participants in the corresponding treatment arm.
3. Inclusion of centre as a random effect.

### Co-primary outcome ii) Time to first significant flare (returning to baseline o-SCORAD) during the 24 weeks after treatment cessation in the MTX vs CyA groups

### Derivation

This outcome refers to participants who re-started a systemic treatment or obtained an o-SCORAD score which is either:

The same as their score at baseline or

Worse (higher) than their score at baseline.

Time to first flare will be calculated from date of end of treatment to last trial visit following treatment cessation.

### Analysis

This is a time-to-event outcome. The event time will be calculated by subtracting the end of treatment date from the date of event as defined above. Not all participants will have an event; participants that do not will be classed as censored.

Censoring date will be calculated by subtracting the end of treatment date from the earliest date of the following:

- Date of final visit for participants that completed the 24 weeks of the trial following treatment cessation without observing flare.
- Date of last trial visit for participants that are lost to follow-up and did not have an event.
- Date of last trial visit for participants that withdrew during the 24 week period following treatment cessation.

A censoring indicator will be created for each trial participant as below:

$$censor=\left\{ \begin{aligned} 1 if flared \\ 0 if censored \end{aligned} \right.$$

A continuity correction of 1 day will be added to all censor/event times in order to include participants who failed or were censored at time=0.

Line listings for each participant that flared following treatment cessation or withdrew from follow-up will be presented detailing: randomisation number, site, date of randomisation, o-SCORAD result at baseline, date of discontinuation/event, o-SCORAD result (at date of event if applicable) and further details.

A Kaplan-Meier plot will be constructed with separate curves for each treatment group and will be presented in the trial report. The units of time for the *x*-axis will be weeks. From inspection of the Kaplan-Meier plot the separation between the curves should remain proportional across analysis time. If the curves cross again this shows that the proportional hazards assumption has been violated. To put this formally, the proportional hazards assumption is that the ratio of hazards is a constant that does not depend on time:

$$\frac{h_{A}(t)}{h_{B}(t)}=r$$

When this assumption fails, it is because the hazard ratio changes over time. To test this, we will add predictor for group*time interaction i.e. a time-dependent covariate. Evidence that group*time interaction is not zero is evidence against proportional hazards. In SAS, PROC PHREG provides the p-value of this test. p<0.05 will indicate non-proportional hazards and thus a time-dependent covariate will be included in the Cox model. If p≥0.05 survival estimates will be calculated using the method of Kaplan-Meier.

In both situations, survival estimates will be calculated using the method of Kaplan-Meier with curves for each treatment group presented graphically with numbers at risk. Survival times (presented in weeks) will be measured from the date of end of treatment to the date of flare as identified above. The p-value obtained from the log-rank test and also the hazard ratio with 97.5% confidence interval will be used to assess differences in failure estimates across treatment groups. The null hypothesis is that there is no difference in outcome between the two treatment groups. The alternative hypothesis is that there is a difference between the two treatment groups. The 2-sided significance level of 0.025 will be used to indicate statistical significance for the comparison between the two treatment groups.

The following sensitivity analyses will be carried out for this outcome:

1. The analysis will be carried out in the subset of those who completed 36 weeks treatment.
2. Those who stopped trial treatment early but continued to take one MTX or CyA would begin the 24 week follow-up period from the end date of the con-med as recorded on the con-med form.

### Change in atopic eczema severity using the EASI, IGA, o-SCORAD and POEM between baseline and 12, 36, 48, and 60 weeks

### Derivation

The EASI score can be found on *Form 6: EASI*. The IGA score can be found on *Form 5: IGA.* The o-SCORAD can be found on *Form 7: o-SCORAD*.

The POEM is participant-assessed and recorded on the *Baseline POEM* form at baseline and in the participant diaries thereafter. The POEM consists of 7 questions and each answer is scored from 0 to 4 as follows:

No days = 0
1-2 days = 1
3-4 days = 2
5-6 days = 3
Every day = 4.

If a question is left unanswered then it will be assigned a score of 0. The scores will be summed and expressed out of a maximum of 28. If two or more questions are left unanswered then the questionnaire is not scored. If two or more response options are selected the response option with the highest score will be used [5].

### Analysis

Mean profile plots will be presented for each of the outcome measures, split by treatment. The difference in score (for the EASI, o-SCORAD and POEM) at each time point will be assessed using linear mixed models including a random intercept to allow for within-participant correlations at different visits. The covariates in the models will be the baseline score and an interaction between treatment group and visit in order to estimate the treatment effect at each time point. Unstructured covariance matrices will be used.

Area under the curve (AUC) analyses will also be carried out for these outcomes.

IGA score will be analysed using ordinal longitudinal regression.

### Number of parent-reported flares in each study arm as well as the proportion of children who re-flared during the 24 weeks after treatment cessation

### Derivation

The number of parent-reported flares in each study arm refers to the number of patient/parent reported flares as recorded within the participant diaries during the 24 weeks post treatment cessation.

N.B. The proportion of children who re-flare refers to the proportion of participants who returned to baseline o-SCORAD or worse within the 24 weeks post treatment cessation or restarted a systemic treatment.

### Analysis

Analyses will be restricted to those who completed more than 80% of the diaries. Patterns of missingness will be explored. The number of flares in each study arm will be compared between treatment arms using a T-test. The number of flares which resulted in the participant’s topical steroid or calcineurin inhibitor treatments being used more frequently or increased in strength will also be reported and compared between treatment groups using a T-test. The mean number of days the participant was on topical steroids; emollients and topical calcineurin inhibitors will be reported for each treatment group.

The mean number of times the participant visited a health care professional because of their eczema will be compared between the two groups using a T-test. The mean number of times the doctor/nurse diagnosed a skin infection and whether antibiotic creams or tablets were prescribed for the skin infection will be summarised by treatment group.

Normality of residuals will be checked by inspection of histograms and Q-Q plots. If residuals do not appear normally distributed the non-parametric Mann-Whitney U-test will be used. The assumption of homoscedasticity will be assessed by inspection of scatter plots of the residuals against the fitted values. Where scatter plots suggest non-constant residual variance, sandwich estimators will be used to estimate standard errors and 95% confidence intervals. To test whether there is an association between proportion of participants returning to baseline o-SCORAD score or worse or restarted a systemic treatment and treatment arm, Chi-squared tests will be used and the p-value presented. If the number of participants who return to baseline o-SCORAD score or worse is:

- Less than 5 in either arm then Fisher’s Exact test will be used instead of the Chi-squared test.
- Zero in one arm then no formal statistical analysis will be carried out but proportions will be reported.

To test the extent of association, logistic regression models will be used to produce odds ratio (OR) estimates with corresponding 95% confidence intervals and p-values.

### Proportion of participants achieving 50% improvement in the o-SCORAD index at 12, 36, 48, and 60 weeks

### Derivation

Participants achieve 50% improvement in o-SCORAD score if they experience a decrease in score of at least 50% from baseline to the specified time points.

### Analysis

Mean profile plots will be presented for this outcome, split by treatment. The difference in proportion at each time point will be assessed using linear mixed models including a random intercept to allow for within-participant correlations at different visits. The covariates in the models will be the baseline score and an interaction between treatment group and visit in order to estimate the treatment effect at each time point. Unstructured covariance matrices will be used.

### Proportion of participants who withdraw from treatment because of AEs

### Derivation

Participants who discontinue early from treatment, with reasons for discontinuation, will be recorded on *Form 10: End of Treatment form*. If the participant discontinues early from the trial due to an Adverse Event (AE), they will be included in the numerator. The denominator will be the total number of participants who were randomised to each arm and received at least one dose of trial treatment.

### Analysis

The proportions will be compared between treatment arms using the analysis specified in section 17.4.4.2.

### Disease-specific patient and parental quality of life (QoL) measured with the CDLQI/IDQOL and DFI scores between baseline and 12, 36, 48 & 60 weeks

### Derivation

**CDLQI**

The CDLQI is a participant completed questionnaire, calculated by summing the score for each of the questions in the following way [6]:

| Very much | 3 |
| --- | --- |
| Quite a lot | 2 |
| Only a little | 1 |
| Not at all | 0 |
| Question unanswered | 0 |
| Question 7: “Prevented School” | 3 |

The maximum score is 30 and the minimum is 0. A high score indicates a more impaired quality of life.

The following six sections will also be considered separately:

| **Section** | **Questions** | **Maximum score** |
| --- | --- | --- |
| Symptoms and feelings | 1, 2 | 6 |
| Leisure | 4, 5, 6 | 9 |
| School or Holidays | 7 | 3 |
| Personal relationships | 3, 8 | 6 |
| Sleep | 9 | 3 |
| Treatment | 10 | 3 |

The severity bandings are as follows:

| 0-1 | no effect on child’s life |
| --- | --- |
| 2-6 | small effect |
| 7-12 | moderate effect |
| 13-18 | very large effect |
| 19-30 | extremely large effect |

The following rules will be applied to incorrectly completed questionnaires.

- Unanswered questions will be scored 0.
- Questionnaires with 2 or more questions unanswered will not be scored.
- Questionnaires with both parts of question 7 completed should take into account the higher of the two scores and discount the lower.

**IDQoL**

The Infant’s Dermatitis Quality of Life Index Questionnaire (IDQoL) is a questionnaire completed by the participant’s parent(s) or regular carer [7].

The IDQoL consists of two sections: Dermatitis Severity and Life Quality Index.

Dermatitis Severity is scored in the following way:

| Extremely Severe | 4 |
| --- | --- |
| Severe | 3 |
| Average | 2 |
| Fairly good | 1 |
| None | 0 |

Life Quality Index is scored in the following way:

**Question 1**

| All the time | 3 |
| --- | --- |
| A lot | 2 |
| A little | 1 |
| None | 0 |

**Question 2**

| Always crying etc. | 3 |
| --- | --- |
| Very fretful | 2 |
| Slightly fretful | 1 |
| Happy | 0 |

**Question 3**

| More than 2 hours | 3 |
| --- | --- |
| 1-2 hours | 2 |
| 15 mins-1 hour | 1 |
| 0-15 mins | 0 |

**Question 4**

| 5 hrs or more | 3 |
| --- | --- |
| 3-4 hours | 2 |
| 1-2 hours | 1 |
| Less than 1 hour | 0 |

**Questions 5-10**

| Very much | 3 |
| --- | --- |
| A lot | 2 |
| A little | 1 |
| Not at all/none | 0 |

The IDQoL score is calculated by summing the individual scores, giving a maximum score of 30 and a minimum of 0. A higher score indicates a higher impairment of quality of life. A similar approach described for the CDQLI will be used for incorrectly completed questionnaires:

- Unanswered questions will be scored 0.
- Questionnaires with 2 or more questions unanswered will not be scored.
- Questions with more than one option completed should take into account the highest of the scores and discount the lower scores.

**DFI**

The DFI is a questionnaire completed by the mother/father or carer of the participant, consisting of 10 questions scored as follows [8]:

| Very much | 3 |
| --- | --- |
| A lot | 2 |
| A little | 1 |
| Not at all | 0 |

The maximum score is 30 indicating a higher effect on life of family compared to the minimum score of 0 indicating no impact on life of family. The same approach described for the IDQoL will be used for incorrectly completed questionnaires.

### Analysis

Mean profile plots will be presented for each of the outcome measures, split by treatment group. The difference in score (for the CDLQI and DFI) at each time point will be assessed using linear mixed models including a random intercept to allow for within-participant correlations at different visits. The covariates in the models will be the baseline score and an interaction between treatment group and visit in order to estimate the treatment effect at each time point. Unstructured covariance matrices will be used.

IDQoL will be summarised descriptively.

### Assess the cost-effectiveness of CyA vs MTX, using a cost/QALY analysis

This analysis will be carried out by the Health Economist and is therefore not detailed in this SAP.

### Immuno-metabolic effects of MTX and CyA, especially in relation to markers of glycolytic activation and T cell cytokine signature, at baseline, during treatment and up to 24 weeks after completion of treatment.

- - - 1. **Derivation**

Blood and tape stripping samples were taken at baseline and weeks 12, 36 and 60 for mechanistic work.

The percentage of cells for the following receptors^[[1]](#footnote-1)^ will be the outcome measures from the blood samples:

- Intracellular cytokines
- Extracellular Type 17
- Extracellular Type 2
- Extracellular Skin homing
- Extracellular Treg

The concentration of cytokines, lipids and filaggrin degradation products will be the outcome measures from the tape stripping samples.

- - - 1. **Analysis**

Summary statistics, individual profile plots and box plots, of percentages of receptor cells over time, will be presented, split by treatment group.

### Drug-related side effects of both MTX and CyA and their association with MTX polyglutamate and CyA trough levels.

### Derivation

Drug-related side effects are adverse events reported as possibly, probably or almost certainly related to treatment.

MTX polyglutamate and CyA trough levels are collected at weeks 2, 8, 12 and 36.

### Analysis

Mean profile plots (or box plots if data are skewed) will be presented for MTX polyglutamate and CyA trough levels over time. The MTX and CyA groups will be analysed separately.

A linear mixed model will be used where the outcome variable is trough level measurement and the explanatory variables are visit and whether there was at least one drug related side effect since the previous visit.

### The association between MTX polyglutamate and CyA trough levels and treatment response (reduction in disease severity)

### Derivation

MTX polyglutamate and CyA trough levels are collected at week 8, week 12 and week 36. Treatment response will be defined as:

1. o-SCORAD score
2. EASI score.

### Analysis

The two treatment groups will be analysed separately. A linear mixed model will be used where the outcome variable is treatment response and the explanatory variables are MTX polyglutamate/CyA trough level over time and baseline o-SCORAD (or EASI) score; an interaction between time and MTX polyglutamate/CyA trough level will be included.

### The association between FLG carriage (yes/no) and treatment response

### Derivation

Filaggrin is measured via a blood/saliva sample at one point during the study. Treatment response will be defined as:

1. o-SCORAD score
2. EASI score

### Analysis

Linear regression models will be used where the outcome variables will be treatment response at 12, 36 and 60 weeks.

The explanatory variables will be FLG carriage (Yes/No), treatment allocation, baseline o-SCORAD (or EASI) score. An interaction between treatment allocation and FLG carriage will be included to see whether the effect of treatment differs depending on FLG carriage.

# Missing data and withdrawals

Line listings of patient withdrawals of consent for follow-up and/or previously collected data with reasons and date of withdrawal will be presented split by treatment group.

For each time point the expected and actual number of assessments corresponding to the primary or secondary outcomes at each time-point will be presented. Reasons for missing primary outcome data will be summarised in a table.

# Additional analyses

At this stage there are no planned additional analyses.

# Safety Evaluations

### Data sets analysed

The safety analysis data set will contain all participants that are randomised and commenced treatment. Participants’ safety events will be included in the treatment arm they were actually receiving at the time of event onset to take into account any patients that crossed over from one group to another. Adverse events will be reported from randomisation until week 40.

### Presentation of the data

Adverse Events (AEs) are captured on the CRFs as free-text. These events are categorised by the Data Manager using MedDRA coding with Chief Investigator input and subsequently signed off by Chief Investigator once complete.

All adverse events (AEs) or adverse reactions (ARs) and serious adverse events (SAEs) reported by the clinical investigator will be presented in a table. The number (and percentage) of patients experiencing each AE/SAE will be presented for each treatment arm categorised by severity (mild, moderate, severe). For each patient, only the maximum severity experienced of each type of AE will be displayed. The number (and percentage) of occurrences of each AE/SAE will also be presented for each treatment arm. No formal statistical testing will be undertaken.

# Quality Control

To ensure quality control (QC), an independent statistician will follow this SAP to independently program the primary outcome and safety analyses from the raw data. Any discrepancies found will be discussed with the trial statistician to resolve. No programming will be shared or shown between the statisticians. The independent statistician will also check the report against their output obtained from the statistical software.

# References

1. International Conference on Harmonisation. Topic E3 Structure and content of clinical study reports.(CPMP/ICH/137/95). 1995
2. International Conference on Harmonisation. Topic E9 Statistical Principles for Clinical Trials (CPMP/ICH/363/96). 1998.
3. Guidance on Statistical Analysis Plans.
4. Schulz KF, Altman DG, Moher D, for the CONSORT Group. CONSORT 2010 Statement: updated guidelines for reporting parallel group randomised trials. [BMJ 2010;340:c332](http://www.bmj.com/content/340/bmj.c332)..
5. <https://www.nottingham.ac.uk/research/groups/cebd/resources/poem.aspx>. Accessed on 09/01/2017.
6. <http://sites.cardiff.ac.uk/dermatology/quality-of-life/childrens-dermatology-life-quality-index-cdlqi/cdlqi-information-and-instructions/>. Accessed on 11/01/2017.
7. <http://sites.cardiff.ac.uk/dermatology/quality-of-life/the-infants-dermatitis-quality-of-life-index-idqol/idqol-information-and-instructions/>. Accessed on 11/01/2017.
8. <http://sites.cardiff.ac.uk/dermatology/quality-of-life/dermatitis-family-impact-questionnaire-dfi/dfi-information-and-instructions/>. Accessed on 11/01/2017.

TREAT Protocol v7.0 29/07/2020

**EudraCT Number:** 2015-002013-29

A Randomised Controlled Trial Assessing the Effectiveness, Safety and Cost-effectiveness of Methotrexate versus Ciclosporin in the Treatment of Severe Atopic Eczema in Children:

The **TRE**atment of Severe **A**topic Eczema **T**rial (TREAT)


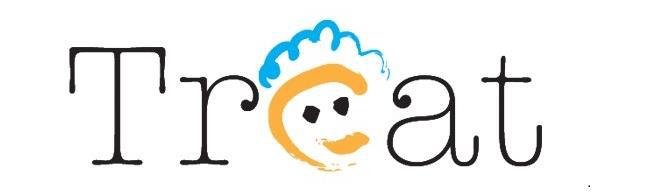


**Version 7.0 29^th^ July 2020**

| **Study Co-Sponsor(s):** | **EudraCT number:** | 2015-002013-29 |
| --- | --- | --- |
| King’s College London and Guy’s and St. | **ISRCTN number:** | 15837754 |
| Thomas’ NHS Foundation Trust |  |  |

**Other:**

**
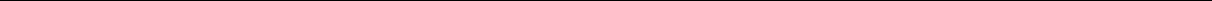

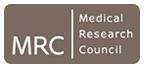

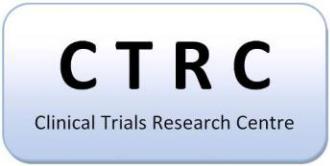

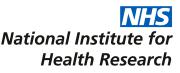

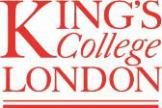
**

IRAS: 182752 1

TREAT Protocol v7.0 29/07/2020

**EudraCT Number:** 2015-002013-29

**Protocol Approval**

**Authorised by Chief Investigator:**

**Signature:** ___________________________________ **Date:** _____________

Professor Carsten Flohr, FRCPCH FRCP MA MSc PhD

Consultant & Reader in Paediatric Dermatology

Department of Paediatric Dermatology

St John’s Institute of Dermatology

King’s College London,

London

**Authorised on behalf of the Lead Statistician**

**Signature:** ___________________________________ **Date:** _____________

Dr Ashley Jones, PhD

Clinical Trials Research Centre

Institute of Child Health

Alder Hey Children’s NHS Foundation Trust

Alder Road

Liverpool

L12 2AP


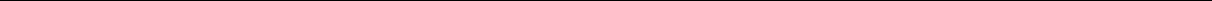


IRAS: 182752 2

TREAT Protocol v7.0 29/07/2020

**EudraCT Number:** 2015-002013-29

**General Information**

This document describes the TREAT trial and provides information about procedures for entering patients into it. The protocol should not be used as an aide-memoir or guide for the treatment of other patients; every care was taken in its drafting, but corrections or amendments may be necessary. These will be circulated to the registered investigators in the trial, but centres entering patients for the first time are advised to contact the coordinating centre (Medicines for Children Clinical Trials Unit) to confirm they have the most up to date version. Clinical problems relating to this trial should be referred to the relevant Chief Investigator via the CTRC.

This protocol defines the participant characteristics required for study entry and the schedule of treatment and follow-up. Participant recruitment will be undertaken in compliance with this document and applicable regulatory and governance requirements and waivers to authorise non-compliance are not permitted.

Incidence of protocol non-compliance, whether reported prospectively (e.g. where a treatment cannot be administered on a scheduled date as a result of public holidays) or retrospectively noted (e.g. as a result of central monitoring) are recorded as protocol deviations, the incidence of which are monitored and reported to trial oversight committees.

**Statement of Compliance**

This study will be carried out in accordance with the World Medical Association Declaration of Helsinki (1964) and the Tokyo (1975), Venice (1983), Hong Kong (1989) and South Africa (1996) amendments and will be conducted in compliance with the protocol, CTRC and KHP CTO Standard Operating Procedures and EU Directive 2001/20/EC, transposed into UK law as the UK Statutory Instrument 2004 No 1031: Medicines for Human Use (Clinical Trials) Regulations 2004 as amended.

**Relationship Statements**

The UK Clinical Research Collaboration (UKCRC; www.ukcrc.org) is a partnership organisation working to establish the UK as a world leader in clinical research. Following a review by an international panel, the Clinical Trials Research Centre (CTRC) at the University of Liverpool has been assessed as reaching the highest quality standard required by the UKCRC and achieved full UKCRC registration.

The CTRC encompasses clinical trials activity in areas including medicines for children (The Medicines for Children Clinical Trials Unit; MC CTU), epilepsy, oral health and obstetrics and gynecology [(http://www.ctrc.org.uk/](http://www.ctrc.org.uk/)). All CTRC activities are underpinned by methodological rigour, a modern data management system, similar technical requirements and a common set of standard operating procedures.

The NIHR Clinical Research Network: Children is part of the National Institute for Health Research Clinical Research Network.

The KHP CTO Quality Team was established in 2008 to manage the Sponsor responsibilities for Clinical Trials of Medicinal Products (CTIMPs), as defined in the Regulations, for trials sponsored or co-sponsored by King’s Health Partner Organisations. Also to facilitate Chief Investigators with the set up and initiation of their trial and to ensure that these trials are conducted according to GCP and the Regulations, throughout the life of the trial.


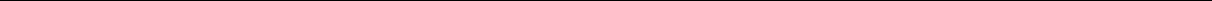


IRAS: 182752 3

TREAT Protocol v7.0 29/07/2020

**EudraCT Number:** 2015-002013-29

**Contact Details: Institutions**

| **Co-Sponsor (Kings College** | **Co-Sponsor (Guys and St** | | | **Trial Management and** |
| --- | --- | --- | --- | --- |
| **London):** | **Thomas’ Foundation NHS** | | | **Monitoring:** |
|  | **Trust):** | | |  |
|  |  |  |  |  |
| Robert Lechler | Jennifer Boston | | | Clinical Trials Research Centre |
| King’s College London | Guy's & St Thomas' | | | (Medicines for Children Clinical |
| University | Foundation NHS Trust | | | Trials Unit) |
| London | R&D Department | | | University of Liverpool Institute of |
| SE1 1UL | 16^th^ Floor, Tower Wing | | | Child Health, |
|  | Great Maze Pond | | | Eaton Road, |
| Tel: 02078486981 | London SE1 9RT | | | Liverpool |
| Email: robert.lechler@kcl.ac.uk |  |  |  | L12 2AP |
|  | Tel: 02071885733 | | |  |
|  | Fax: 02071883472 | | | Tel: 0044 (0) 151 795 8761 |
|  | e-mail: | | | Fax number: 00 44 (0) 151 795 |
|  | [R&D@gstt.nhs.uk](mailto:R&D@gstt.nhs.uk) | | | 8770 |
|  |  |  |  | Email: [treatctu@liv.ac.uk](mailto:treatctu@liv.ac.uk) |
|  |  |  |  |  |
| **Management of Sponsor** | **Research Laboratory Guy’s** | | | **Research Laboratory Guys and** |
| **responsibilities** | **and St Thomas’s Foundation** | | | **St Thomas’s Foundation NHS** |
|  | **NHS Trust (MTX metabolite** | | | **Trust (Urine, creatinine,** |
|  | **analysis))** | | | **cystatin C & CyA trough level** |
|  |  |  |  | **analysis)** |
| Amy Holton | Dr Monica Arenas Hernandez/ | | | Mr Charles Turner/Prof Neil |
| Kings Health Partners Clinical | Dr Tony Marinaki | | | Dalton |
| Trials Office | Purine Research Laboratory, | | | WellChild Laboratory |
| F16, Tower Wing, Guys | Biochemical Sciences, | | | 1st Floor (Arctic) |
| Hospital, Great Maze Pond, | Viapath Analytics, | | | Evelina London Children's |
| London, SE1 9RT | 4^th^ Floor, North wing. | | | Hospital |
| Tel: 020 7188 5732 | St.Thomas’ Hospital | | | St Thomas Hospital |
| Email: amy.holton@kcl.ac.uk | London SE17EH | | | London SE17EH |
|  | Tel: 02071881266 | | | Tel: 02071880159 |
|  | Email:[Monica.Arenas-](mailto:Monica.Arenas-Hernandez@viapath.co.uk) | | | Email: |
|  |  |  |  |  |
|  | [Hernandez@viapath.co.uk](mailto:Monica.Arenas-Hernandez@viapath.co.uk) | | | [charles.turner@gstt.nhs.uk,](mailto:charles.turner@gstt.nhs.uk) |
|  | [Tony.Marinaki@viapath.co.uk](mailto:Tony.Marinaki@viapath.co.uk) | | | [neil.dalton@gstt.nhs.uk](mailto:neil.dalton@gstt.nhs.uk) |
|  |  | | |  |
| **Research Laboratory** | **Research Laboratory London** | | | **Biobanking at King’s College** |
| **Cambridge (metabolic** | **(blood based immunology** | | | **London** |
| **analysis)** | **analysis – mechanistic** | | |  |
|  | **samples)** | | |  |


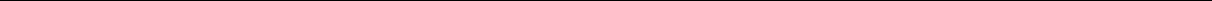


IRAS: 182752 4

TREAT Protocol v7.0 29/07/2020

**EudraCT Number:** 2015-002013-29

**Contact Details: Institutions**

| Dr Christian Frezza | Professor Leonie Taams | John Cason |
| --- | --- | --- |
| MRC Cancer Unit | Centre for Molecular and | The Infectious Diseases BioBank |
| University of Cambridge | Cellular Biology of Inflammation, | Department of Infectious |
| Hutchison/MRC Research | Division of Immunology, | Diseases |
| Centre | Infection & Inflammatory | Kings College London |
| Box 197, Biomedical Campus | Disease, | 2^nd^ Floor, Borough Wing |
| Cambridge, UK | Kings College London, | Guy’s Hospital |
| CB2 0XZ | 1^st^ Floor New Hunt’s House, | London |
|  | Room 1.26F, | SE1 9RT |
| Tel: 01223 763240 | Guy’s Campus, London, SE1 |  |
|  | 1UL | [john.cason@kcl.ac.uk](mailto:john.cason@kcl.ac.uk) |
| [CF366@MRC-CU.cam.ac.uk](mailto:CF366@MRC-CU.cam.ac.uk) | Tel: 020 7848 8633 | Tel: +44 207 188 1180 |
|  | [leonie.taams@kcl.ac.uk](mailto:leonie.taams@kcl.ac.uk) |  |
|  |  |  |
| **Research Laboratory London** | **Research Laboratory** |  |
| **(genotyping)** | **Amsterdam (tape strips)** |  |
|  |  |  |
| Professor Edel O’Toole | Dr Sanja Kezic |  |
| Genome Centre, Blizard | Coronel Institute of Occupational |  |
| Institute, | Health, Amsterdam University |  |
| Barts & the London School of | Medical Centers. Meibergdreef |  |
| Medicine and Dentistry | 9; 1105 AZ |  |
| Queen Mary University Hospital | Amsterdam, |  |
| 4 Newark Street London E1 | The Netherlands |  |
| 2AT |  |  |
|  | [Tel:+31](tel:+31) (0) 205665321 |  |
| Email: [e.a.otoole@qmul.ac.uk](mailto:e.a.otoole@qmul.ac.uk) |  |  |
|  | Email: |  |
|  | [s.kezic@amsterdamumc.nl](mailto:s.kezic@amsterdamumc.nl) |  |
|  |  |  |


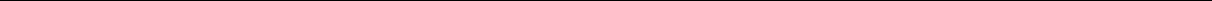


IRAS: 182752 5

TREAT Protocol v7.0 29/07/2020

**EudraCT Number:** 2015-002013-29

**Contact Details: Individuals**

| **Chief Investigator (CI):** | **Trial Nephrologist** |
| --- | --- |
|  |  |
| Professor Carsten Flohr |  |
| Consultant & Chair in Dermatology and | Dr Manish Sinha |
| Population Science |  |
|  | Department of Pediatric Nephrology, |
| Unit for Population-Based Dermatology Research |  |
|  | Evelina London Children’s Hospital, |
| St John’s Institute of Dermatology |  |
|  | Guys’ & St Thomas’ NHS Foundation Trust |
| King’s College London, London, UK |  |
|  | London, SE1 7EH |
| Tel. 07806514078 |  |
|  | Tel: 0207 7188 4587 |
| Fax. 020 7188 7188 ext. 51162 |  |
|  | Email: Manish.Sinha@gstt.nhs.uk |
| Email: [carsten.flohr@kcl.ac.uk](mailto:carsten.flohr@kcl.ac.uk) |  |
|  |  |
|  |  |
| **Medical Expert who will Advise on Protocol** | **Medical Expert who will Evaluate SAE Reports** |
| **Related Clinical Queries (If other than CI):** | **(If other than CI):** |
|  |  |
| Alan Irvine MD | Alan Irvine MD |
| Trinity College Dublin | Trinity College Dublin |
| Paediatric Dermatology | Paediatric Dermatology |
| Our Lady’s Children’s Hospital Crumlin | Our Lady’s Children’s Hospital Crumlin |
| Dublin 12, IRELAND | Dublin 12, IRELAND |
| Phone +3531 428 2532 | Phone +3531 428 2532 |
| Fax +3531 428 2651 | Fax +3531 428 2651 |
| Email: [IRVINEA@tcd.ie](mailto:IRVINEA@tcd.ie) | Email: [IRVINEA@tcd.ie](mailto:IRVINEA@tcd.ie) |
|  |  |


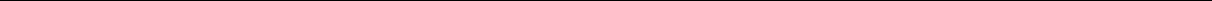


IRAS: 182752 6

TREAT Protocol v7.0 29/07/2020

**EudraCT Number:** 2015-002013-29

**CONTENTS**

| [**1**](#page11) | [**Protocol Summary ................................................................................**](#page11) | | [**11**](#page11) |  |
| --- | --- | --- | --- | --- |
| [**2**](#page15) | [**Background Information ......................................................................**](#page15) | | [**15**](#page15) |  |
|  | [2.1](#page15) | [Introduction ..........................................................................................................](#page15) | | [15](#page15) |
|  | [2.2](#page16) | [Rationale .............................................................................................................](#page16) | | [16](#page16) |
|  | [2.3](#page17) | [Objectives ............................................................................................................](#page17) | | [17](#page17) |
|  | [2.4](#page17) | [Potential Risks and Benefits ................................................................................](#page17) | | [17](#page17) |
| [**3**](#page20) | [**Selection of Centres/Clinicians ...........................................................**](#page20) | | [**20**](#page20) |  |
|  | [3.1](#page20) | [Centre/Clinician inclusion criteria .........................................................................](#page20) | | [20](#page20) |
|  | [3.2](#page20) | [Centre/Clinician Exclusion Criteria .......................................................................](#page20) | | [20](#page20) |
| [**4**](#page21) | [**Trial design............................................................................................**](#page21) | | [**21**](#page21) |  |
| [**5**](#page22) | [**Study Population ..................................................................................**](#page22) | | [**22**](#page22) |  |
|  | [5.1](#page22) | [Inclusion Criteria ..................................................................................................](#page22) | | [22](#page22) |
|  | [5.2](#page22) | [Exclusion Criteria .................................................................................................](#page22) | | [22](#page22) |
| [**6 Enrolment, Randomisation and withdrawal .......................................**](#page24) | | | [**24**](#page24) |  |
|  | [6.1](#page24) | [Recruitment and Screening .................................................................................](#page24) | | [24](#page24) |
|  | [6.2](#page24) | [Screening Visit .....................................................................................................](#page24) | | [24](#page24) |
|  | [6.3](#page25) | [Baseline...............................................................................................................](#page25) | | [25](#page25) |
|  | [6.4](#page26) | [Randomisation and blinding .................................................................................](#page26) | | [26](#page26) |
|  | [6.4.1 Randomisation ....................................................................................................](#page26) | | | [26](#page26) |
|  | [6.4.2 Blinding ...............................................................................................................](#page27) | | | [27](#page27) |
|  | [6.5](#page27) | [Patient Transfer and Withdrawal ..........................................................................](#page27) | | [27](#page27) |
|  | [6.5.1 Patient Transfers .................................................................................................](#page27) | | | [27](#page27) |
|  | [6.5.2 Discontinuation from Trial Intervention ................................................................](#page27) | | | [27](#page27) |
|  | [6.5.3 Withdrawal from Trial Completely ........................................................................](#page29) | | | [29](#page29) |
| [**7**](#page30) | [**Trial Treatments ....................................................................................**](#page30) | | [**30**](#page30) |  |
|  | [7.1](#page30) | [Introduction ..........................................................................................................](#page30) | | [30](#page30) |
|  | [7.2](#page31) | [Arm A - Ciclosporin ..............................................................................................](#page31) | | [31](#page31) |
|  | [7.2.1 Ciclosporin - Formulation, Packaging, Labelling, Storage and Stability ............](#page31) | | | [31](#page31) |
|  | [7.2.2 Preparation, Dosage and Administration of Study Treatment/s ........................](#page31) | | | [31](#page31) |
|  | [7.2.3](#page31) | [Ciclosporin - Dose Modifications ......................................................................](#page31) | | [31](#page31) |
|  | [7.3](#page33) | [Arm B - Methotrexate ...........................................................................................](#page33) | | [33](#page33) |
|  | [7.3.1 Methotrexate - Formulation, Packaging, Labelling, Storage and Stability .........](#page33) | | | [33](#page33) |
|  | [7.3.2](#page33) | [Methotrexate - Preparation, Dosage and Administration of Study Treatment/s 33](#page33) | | |
|  | [7.3.3](#page34) | [Methotrexate - Dose Modifications ...................................................................](#page34) | | [34](#page34) |
|  | [7.4](#page36) | [Unblinding ...........................................................................................................](#page36) | | [36](#page36) |
|  | [7.5](#page36) | [Accountability Procedures for Study Treatments .................................................](#page36) | | [36](#page36) |
|  | [7.6](#page37) | [Assessment of Compliance with Study Treatment/s ............................................](#page37) | | [37](#page37) |
|  | [7.7](#page37) | [Concomitant Medications/Treatments ..................................................................](#page37) | | [37](#page37) |
|  | [7.7.1](#page37) | [Medications Permitted .....................................................................................](#page37) | | [37](#page37) |
|  | [7.7.2 Medications Not Permitted/ Precautions Required ...........................................](#page38) | | | [38](#page38) |
|  | [7.7.3 Data on Concomitant Medication .....................................................................](#page40) | | | [40](#page40) |
|  | [7.8](#page40) | [Co-enrolment Guidelines .....................................................................................](#page40) | | [40](#page40) |
| [**8**](#page41) | [**Assessments and Procedures.............................................................**](#page41) | | [**41**](#page41) |  |
|  | [8.1](#page41) | [Schedule for assessments during the treatment and the follow-up phase ............](#page41) | | [41](#page41) |
|  | [8.1.1](#page43) | [Assessments: ..................................................................................................](#page43) | | [43](#page43) |
|  | [8.1.2](#page43) | [Visit Summary .................................................................................................](#page43) | | [43](#page43) |


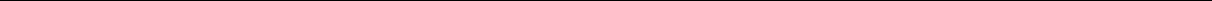


IRAS: 182752 7

TREAT Protocol v7.0 29/07/2020

**EudraCT Number:** 2015-002013-29

| [8.2](#page48) | | [Procedures for Assessing Efficacy.......................................................................](#page48) | | [48](#page48) |
| --- | --- | --- | --- | --- |
| [8.3](#page49) | | [Procedures for Assessing Safety .........................................................................](#page49) | | [49](#page49) |
| [8.4](#page49) | | [Other Assessments .............................................................................................](#page49) | | [49](#page49) |
|  | [8.4.1](#page49) |  | [Quality of Life ...................................................................................................](#page49) | [49](#page49) |
|  | [8.4.2](#page50) |  | [Health Economics ............................................................................................](#page50) | [50](#page50) |
|  | [8.4.3 Special Assays or Procedures .........................................................................](#page51) | | | [51](#page51) |
| [8.5](#page52) | | [Loss to Follow-up ................................................................................................](#page52) | | [52](#page52) |
| [8.6](#page52) | | [Trial Closure ........................................................................................................](#page52) | | [52](#page52) |
| [**9**](#page54) | [**Statistical Considerations ....................................................................**](#page54) | | | [**54**](#page54) |
| [9.1](#page54) | | [Introduction ..........................................................................................................](#page54) | | [54](#page54) |
| [9.2](#page54) | | [Method of Randomisation ....................................................................................](#page54) | | [54](#page54) |
| [9.3](#page54) | | [Outcome Measures .............................................................................................](#page54) | | [54](#page54) |
| [9.4](#page54) | | [Co-Primary Endpoints ..........................................................................................](#page54) | | [54](#page54) |
| [9.5](#page54) | | [Secondary Endpoint(s) ........................................................................................](#page54) | | [54](#page54) |
| [9.6](#page55) | | [Sample Size ........................................................................................................](#page55) | | [55](#page55) |
| [9.7](#page55) | | [Interim Monitoring and Analyses ..........................................................................](#page55) | | [55](#page55) |
| [**10**](#page57) | [**Pharmacovigilance ...............................................................................**](#page57) | | | [**57**](#page57) |
| [10.1](#page57) | | [Terms and Definitions ..........................................................................................](#page57) | | [57](#page57) |
| [10.2](#page58) | | [Notes on Adverse Event Inclusions and Exclusions .............................................](#page58) | | [58](#page58) |
|  | [10.2.1](#page58) | | [Include .........................................................................................................](#page58) | [58](#page58) |
|  | [10.2.2](#page58) | | [Do Not Include .............................................................................................](#page58) | [58](#page58) |
|  | [10.2.3](#page58) | | [Reporting of Pregnancy ...............................................................................](#page58) | [58](#page58) |
| [10.3](#page59) | | [Notes Severity / Grading of Adverse Events ........................................................](#page59) | | [59](#page59) |
| [10.4](#page59) | | [Relationship to Trial Treatment ............................................................................](#page59) | | [59](#page59) |
| [10.5](#page59) | | [Expectedness ......................................................................................................](#page59) | | [59](#page59) |
| [10.6](#page60) | | [Follow-up After Adverse Events ...........................................................................](#page60) | | [60](#page60) |
| [10.7](#page60) | | [Reporting Procedures ..........................................................................................](#page60) | | [60](#page60) |
|  | [10.7.1](#page60) | | [Non serious ARs/AEs ..................................................................................](#page60) | [60](#page60) |
|  | [10.7.2](#page60) | | [Serious ARs/AEs/SUSARs ..........................................................................](#page60) | [60](#page60) |
| [10.8](#page61) | | [Responsibilities – Investigator .............................................................................](#page61) | | [61](#page61) |
|  | [10.8.1](#page62) | | [Maintenance of Blinding ...............................................................................](#page62) | [62](#page62) |
| [10.9](#page62) | | [Responsibilities – CTRC and KHP CTO ..............................................................](#page62) | | [62](#page62) |
|  | [10.9.1](#page63) | | [Safety reports ..............................................................................................](#page63) | [63](#page63) |
| [10.10](#page63) | |  | [Contact Details and Out-of-hours Medical Cover .............................................](#page63) | [63](#page63) |
| [**11**](#page64) | [**Ethical Considerations .........................................................................**](#page64) | | | [**64**](#page64) |
| [11.1](#page64) | | [Ethical Considerations .........................................................................................](#page64) | | [64](#page64) |
| [11.2](#page64) | | [Ethical Approval ...................................................................................................](#page64) | | [64](#page64) |
| [11.3](#page65) | | [Informed Consent Process ..................................................................................](#page65) | | [65](#page65) |
|  | [11.3.1](#page66) | | [Consent for 16 year olds ..............................................................................](#page66) | [66](#page66) |
|  | [11.3.2](#page66) | | [Assent in minors ..........................................................................................](#page66) | [66](#page66) |
| [11.4](#page66) | | [Study Discontinuation ..........................................................................................](#page66) | | [66](#page66) |
| [**12**](#page67) | [**Regulatory Approval ............................................................................**](#page67) | | | [**67**](#page67) |
| [**13**](#page68) | [**Trial Monitoring .....................................................................................**](#page68) | | | [**68**](#page68) |
| [13.1](#page68) | | [Risk Assessment .................................................................................................](#page68) | | [68](#page68) |
| [13.2](#page68) | | [Source Documents ..............................................................................................](#page68) | | [68](#page68) |
| [13.3](#page69) | | [Data Capture Methods .........................................................................................](#page69) | | [69](#page69) |
|  | [13.3.1](#page69) | | [Case Report Forms ......................................................................................](#page69) | [69](#page69) |
| [13.4](#page69) | | [Central Monitoring ...............................................................................................](#page69) | | [69](#page69) |
| [13.5](#page70) | | [Clinical Site Monitoring ........................................................................................](#page70) | | [70](#page70) |
|  | [13.5.1](#page70) | | [Confidentiality ..............................................................................................](#page70) | [70](#page70) |
|  | [13.5.2](#page70) | | [Quality Assurance and Control .....................................................................](#page70) | [70](#page70) |
| [13.6](#page71) | | [Records Retention ...............................................................................................](#page71) | | [71](#page71) |


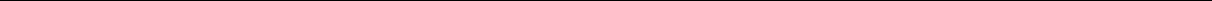


IRAS: 182752 8

TREAT Protocol v7.0 29/07/2020

**EudraCT Number:** 2015-002013-29

| [**14**](#page72) | [**Indemnity ...............................................................................................**](#page72) | | [**72**](#page72) |
| --- | --- | --- | --- |
| [**15**](#page73) | [**Financial Arrangements .......................................................................**](#page73) | | [**73**](#page73) |
| [15.1](#page73) | | [Collaborating Centre Payments ...............................................................................](#page73) | [73](#page73) |
| [15.2](#page73) | | [Research Team .......................................................................................................](#page73) | [73](#page73) |
| [**16**](#page74) | [**Trial Committees ...................................................................................**](#page74) | | [**74**](#page74) |
| [16.1](#page74) | | [Trial Management Group (TMG) ..........................................................................](#page74) | [74](#page74) |
| [16.2](#page74) | | [Trial Steering Committee (TSC) ...........................................................................](#page74) | [74](#page74) |
| [16.3](#page74) | | [Independent Data and Safety Monitoring Committee (IDSMC) ............................](#page74) | [74](#page74) |
| [**17**](#page75) | [**Publication ............................................................................................**](#page75) | | [**75**](#page75) |
| [**18**](#page76) | [**Protocol Amendments .........................................................................**](#page76) | | [**76**](#page76) |
| [18.1](#page76) | | [Version 2 (08/10/2015) ........................................................................................](#page76) | [76](#page76) |
| [18.2](#page76) | | [Version 3.0 23/10/2015 ........................................................................................](#page76) | [76](#page76) |
| [18.3](#page77) | | [Version 4.0 17/10/2016 ........................................................................................](#page77) | [77](#page77) |
| [18.4](#page79) | | [Version 5.0 29/06/2017 ........................................................................................](#page79) | [79](#page79) |
| [18.5](#page80) | | [Version 6.0 18/02/2019 ........................................................................................](#page80) | [80](#page80) |
| [18.6](#page81) | | [Version 7.0 29/07/2020 ........................................................................................](#page81) | [81](#page81) |
| [**19**](#page82) | [**References ............................................................................................**](#page82) | | [**82**](#page82) |

**
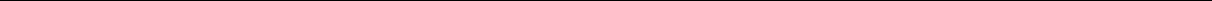
**

IRAS: 182752 9

TREAT Protocol v7.0 29/07/2020

**EudraCT Number:** 2015-002013-29

**Glossary**

| AE | Adverse Event |
| --- | --- |
| AR | Adverse Reaction |
| AZA | Azathioprine |
| CDLQI | Children’s Dermatology Life Quality Index |
| CI | Chief Investigator |
| CHU-9D | Child Health Utility – Nine Dimensions |
| CRF | Case Report Form |
| CS | Corticosteroids |
| CTRC | Clinical Trials Research Centre |
| CYA | Ciclosporin A |
| DFI | Dermatology Family Index |
| DQOL | Dermatology Quality of Life questionnaire |
| EASI | Eczema Area Severity Index |
| FLG | Filaggrin |
| GP | General Practitioner |
| IB | Investigator’s Brochure |
| IDSMC | Independent Data and Safety and Monitoring Committee |
| IEC | Independent Ethical Committee |
| IGA | Investigator Global Assessment |
| IMP | Investigational Medicinal Product |
| KHP CTO | King’s Health Partners Clinical Trials Office |
| MC CTU | Medicines for Children Clinical Trials Unit |
| MTX | Methotrexate |
| MREC | Multi-centre Research Ethics Committee |
| NIHR CRN | National Institute for Health Research Clinical Research Network |
| PI | Principal Investigator |
| PML | Progressive Multifocal Leukoencephalopathy |
| QOL | Quality of Life |
| R&D | Research & Development |
| RCT | Randomised Controlled Trial |
| REC | Research Ethics Committee |
| RN | Research Nurse. When RN is referred to in this protocol it means |
|  | either the RN or someone who has been delegated that duty. |
| SAE | Serious Adverse Event |
| SAR | Serious Adverse Reaction |
| SCORAD | SCORing Atopic Dermatitis severity index |
| SDV | Source Data Verification |
| SPC | Summary of product characteristics |
| SUSAR | Suspected Unexpected Serious Adverse Reaction |
| TB | Tuberculosis |
| TMF | Trial Master File |
| TMG | Trial Management Group |
| TPMT | Thiopurine Methyltransferase |
| TSC | Trial Steering Committee |
| UAR | Unexpected Adverse Reaction |


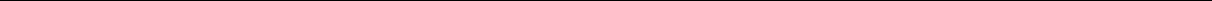


IRAS: 182752 10

TREAT Protocol v7.0 29/07/2020

**EudraCT Number:** 2015-002013-29

**1** **PROTOCOL SUMMARY**

| **Study title:** | Randomised controlled trial assessing the effectiveness, |
| --- | --- |
|  | safety and cost-effectiveness of methotrexate (MTX) |
|  | versus ciclosporin (CyA) in the treatment of severe atopic |
|  | eczema in Children: The **TRE**atment of Severe **A**topic |
|  | Eczema **T**rial (TREAT) |
| **Protocol Short Title/Acronym:** | TREAT Trial |
|  |  |
| **Phase:** | III |
| **Co-Sponsor names:** | King’s College London and Guy’s & St Thomas’ NHS |
|  | Foundation Trust |
|  |  |
| **Chief Investigator:** | Professor Carsten Flohr |
|  |  |
| **Eudract number:** | 2015-002013-29 |
|  |  |
| **REC number:** | 15/EE/0328 |
|  |  |
| **Study design:** | Randomised multicentre trial comparing methotrexate |
|  | (MTX) and ciclosporin (CyA) in patients with severe |
|  | paediatric atopic eczema. |
|  | 102 participants will be randomised applying a ratio of |
|  | 1:1. |
| **Population:** | Children age 2-16 years with severe atopic eczema (o- |
|  | SCORAD≥30) and inadequately responding to potent |
|  | topical treatment. Children should not have received UV |
|  | light therapy within the past 6 months & should never |
|  | have received any systemic immuno-suppressive therapy, |
|  | except for oral corticosteroids for acute flares. |
| **Centres and Distribution:** | Secondary and tertiary paediatric dermatology centres. |
|  |  |
| **Study Duration:** | All participants will be treated for 36 weeks on either |
|  | therapy, with another 24 weeks follow up post treatment |
|  | cessation (total duration 60 weeks per participant). |
| **Intervention:** | 1. CyA: 4mg/kg/day (in divided doses); |
|  | OR |
|  | 2. MTX: started at 0.1mg/kg/week (initial test dose) |
|  | and then continued on 0.4mg/kg/week from week |
|  | 1 (Max dose = 25mg/week)). Tablet or oral |
|  | solution as standard, but MTX can be given |
|  | subcutaneously if gastrointestinal side effects |
|  | warrant this. |
| **Primary Objective:** | The study has two co-primary objectives. |
|  | 1. To assess the change in atopic eczema severity |
|  | between baseline & 12 weeks of treatment in the |
|  | two treatment arms, and |
|  | 2. To examine disease remission (time to first |
|  | significant flare) after treatment cessation in the |
|  | MTX vs CyA groups. |
| **Secondary Objective/s:** | -To examine atopic eczema severity using the Eczema |
|  | Area & Severity Index (EASI), Investigator Global |
|  | Assessment (IGA), objective SCORing Atopic Dermatitis |
|  | (o-SCORAD) & Patient Orientated Eczema Measure |


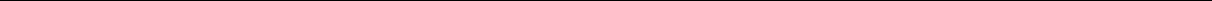


IRAS: 182752 11

TREAT Protocol v7.0 29/07/2020

**EudraCT Number:** 2015-002013-29

|  | (POEM) scores between 0 and 12, 36, 48, 60 weeks and | |
| --- | --- | --- |
|  | using the o-SCORAD at 36, 48 and 60 weeks | |
|  | -To compare the number of flares in each study arm as | |
|  | well as the proportion of children who re-flared following | |
|  | treatment cessation. | |
|  | -To study the impact on quality of life: change in | |
|  | CDLQI/IDQOL & DFI scores between 0, 12, 36, 48 and | |
|  | 60 weeks | |
|  | -To determine the proportion of participants achieving | |
|  | 50% improvement in the o-SCORAD and EASI index at | |
|  | 12, 36, 48, and 60 weeks. | |
|  | -To capture the proportion of participants who withdraw | |
|  | from treatment because of AEs. | |
|  | -To assess the cost-effectiveness of CyA vs MTX based | |
|  | on utility measured using the CHU-9D. | |
|  | -To study the immuno-metabolic effects of MTX and CyA, | |
|  | especially in relation to markers of glycolytic activation | |
|  | and T cell cytokine signature, at baseline, during | |
|  | treatment and after completion of treatment. | |
|  | -To compare the drug side effects/toxicity profiles of both | |
|  | MTX and CyA, | |
|  | - To examine the association between MTX | |
|  | polyglutamate and CyA trough levels and reduction in | |
|  | atopic eczema severity as well as drug-related side | |
|  | effects, | |
|  | and | |
|  | -To study the impact of FLG carriage (yes/no) on | |
|  | reduction in atopic eczema severity. | |
| **Endpoints:** | Co-Primary Endpoints: |  |
|  | 1. Change in atopic eczema severity between baseline | |
|  |  |  |
|  | and 12 weeks of treatment, using the o-SCORAD index. | |
|  | 2. Time to first significant flare after treatment cessation | |
|  | in the MTX vs CyA groups. | |

Secondary Endpoints:

1. Change in atopic eczema severity using the EASI, IGA, o-SCORAD and POEM between baseline and 12, 36, 48, and 60 weeks.
2. Number of flares in each study arm as well as the proportion of children who re-flared following treatment cessation.
3. Disease-specific patient and parental quality of life (QoL) measured with the CDLQI/IDQOL, DFI scores between baseline and 12, 36, 48 & 60 weeks.
4. Proportion of participants achieving 50% improvement in the o-SCORAD and EASI index at 12, 36, 48, and 60 weeks.
5. Proportion of participants who withdraw from treatment because of AEs.
6. Assess the cost/QALY of CyA vs MTX based on utility measured using the CHU-9D.
7. Immuno-metabolic effects of MTX and CyA, especially in relation to markers of glycolytic activation and T cell cytokine signature, at baseline, during treatment and up to 6 months after completion of treatment.


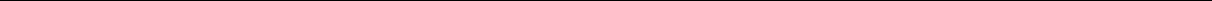


IRAS: 182752 12

TREAT Protocol v7.0 29/07/2020

**EudraCT Number:** 2015-002013-29

|  | 8. Drug-related side effects of both MTX and CyA and |
| --- | --- |
|  | their association with MTX polyglutamate and CyA trough |
|  | levels. |
|  | 9. The association between MTX polyglutamate and CyA |
|  | trough levels and treatment response (reduction in |
|  | disease severity). |
|  | 10. The association between FLG carriage (yes/no) and |
|  | treatment response. |
| **Sample Size:** | 102 |
|  |  |
| **Summary of eligibility criteria:** | 1. Aged 2-16 years at the time of the screening visit |
|  | and randomisation visit |
|  | 2. Diagnosis of severe recalcitrant atopic eczema |
|  | 3. History of inadequate clinical response (in the |
|  | opinion of the treating clinician) to potent topical |
|  | corticosteroids on the body and moderate strength |
|  | topical corticosteroids on the face. |
|  | 4. An objective (o)-SCORAD severity score of at |
|  | least 30 |
| **Version and date of protocol** | V1.0 28/07/2015 |
|  | V2.0 08/10/2015 |
| **amendments:** |  |
|  | V3.0 23/10/2015 |
|  |  |
|  | V4.0 17/10/2016 |
|  | V5.0 29/06/2017 |
|  | V6.0 18/02/2019 |


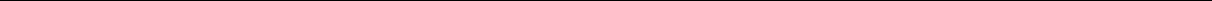


IRAS: 182752 13

TREAT Protocol v7.0 29/07/2020

**EudraCT Number:** 2015-002013-29

| **Protocol Summary - continued** |  | Screening Visit: Participant | |
| --- | --- | --- | --- |
|  |  |  |  |
|  |  | assessed for eligibility criteria. | |
|  |  |  |  |
| **Schematic of Study Design:** |  | Informed consent provided. | |
|  |  |  |  |
|  |  |  |  |


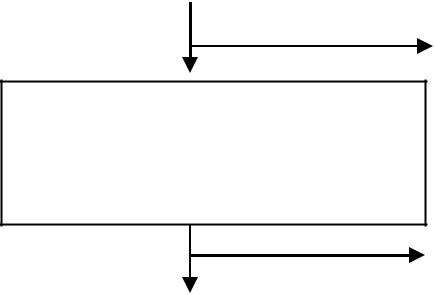


Baseline Visit:

Eligibility

Baseline assessments

Randomisation (n=102)


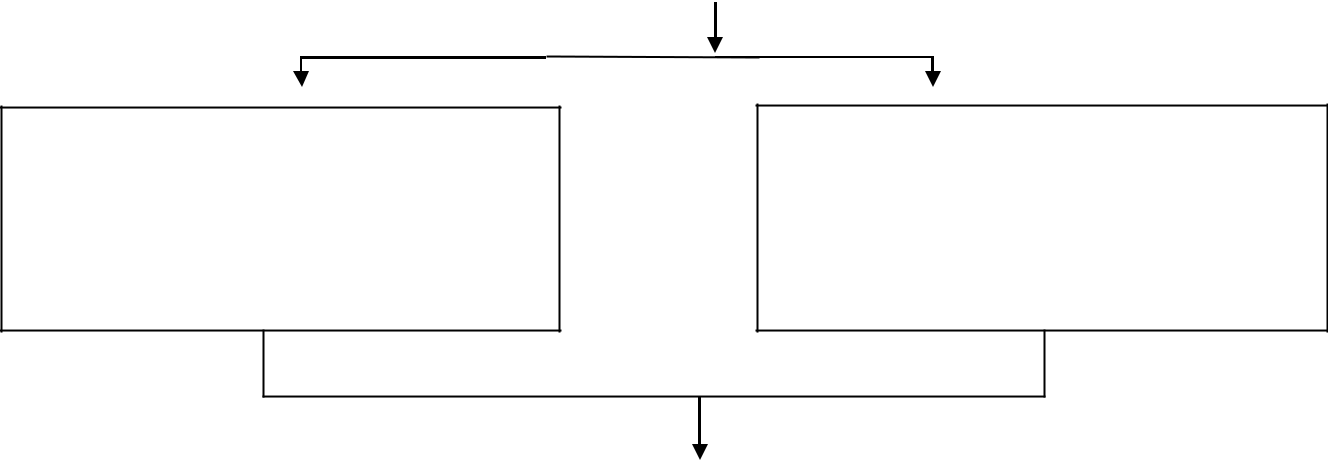


Excluded

Not meeting inclusion criteria Meeting exclusion criteria

Declined to participate Sought other treatment

Excluded

Not meeting inclusion criteria Meeting exclusion criteria

Decided not to participate Sought other treatment

**Ciclosporin n=51**

2mg/kg twice daily (total: 4mg/kg/day)

Frequency: twice daily

Duration: 36 weeks

**Methotrexate n = 51**

Initial test dose of 0.1mg/kg/, then

0.4mg/kg/week from week 1 (max dose =

25mg/week)

Frequency: weekly

Duration: 36 weeks

**Week 1 (for Methotrexate arm only)**

Safety bloods


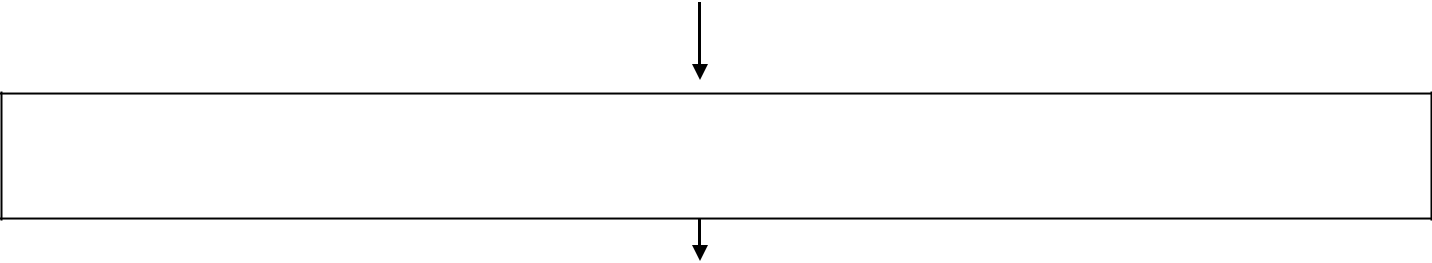


**Week 2**

Safety bloods, urine sample and collection of blood for MTX/CyA metabolite/trough levels


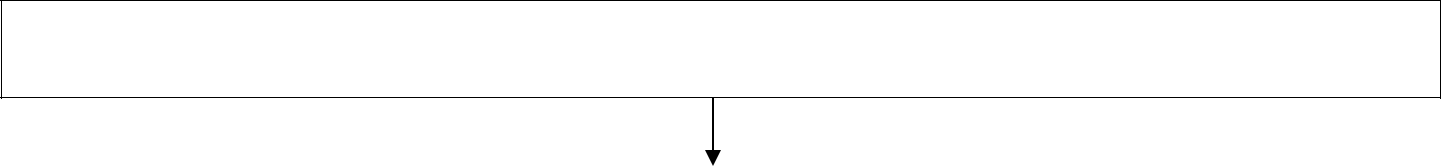


**Week 4 and 8^(1)^**

o-SCORAD, EASI, IGA, POEM, study drug dispensing, safety bloods

**_Week 12_*(1,2,,3,4)*_, 20, 28_*)*_, 36_*(1,2,3,45)*_, 48_**5 **_and 60_*(2,3,4,5)***

o-SCORAD, EASI, IGA, POEM, study drug dispensing, safety bloods

*Key to activities for Week 4, 8, 12, 24, 36, 48 and 60: ^1^ Collection of blood for MTX/CyA metabolite/trough levels, ^2^ Tape stripping for cutaneous metabolic work, ^3^ Collection of blood for mechanistic studies,^4^ Urine sample, ^5^ No study drug dispensing will be performed on week 36, 48 and 60 (Follow-Up phase)*

*
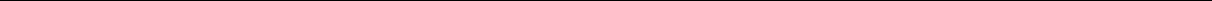
*

IRAS: 182752 14

TREAT Protocol v7.0 29/07/2020

**EudraCT Number:** 2015-002013-29

**2** **BACKGROUND INFORMATION**

**2.1 Introduction**

Background

Atopic eczema is a chronic, pruritic inflammatory skin disease and affects around 20% of UK children, 16% of whom have moderate-severe disease(1). It comes at a high cost, both for the individual patient and their family as well as society at large (~£500mio per year in the UK alone, 21^st^ cause of non-fatal burden of all diseases) (2, 3). Patients with severe atopic eczema suffer significant sleep disturbance, show poor school attendance and are often socially withdrawn and are significantly more likely to suffer of attention-deficit hyperactivity disorder. There is also a clear link with anxiety and depression. Skin infections, in particular with Staphylococcus aureus but also herpes simplex, leading to hospital admissions are another typical feature of poorly controlled eczema(4). Although most cases of atopic eczema are adequately controlled with emollients and topical treatments and/or UV therapy, around 2% of children require oral immuno-suppressive treatment to induce and maintain disease control (5).The European TREAT Survey

The currently available treatment options for severe atopic eczema in children are very limited, and there is concern about their potential short and long-term side effects (5). We consequently conducted the TREatment of severe Atopic eczema in children Taskforce (TREAT) survey among 765 consultant dermatologists and paediatricians from 8 European countries. This showed that 43% of European physicians who look after children with severe atopic eczema use Cyclosporine A (CyA) as their first choice systemic immuno-suppressive agent (6). 31% use oral corticosteroids (CS) and 22% azathioprine (AZA) as 1^st^ choice. However, the situation in the UK is a little different, where 39% use AZA first line compared to 35% for CyA and 19% for CS. Although MTX was only the 3^rd^ most commonly used systemic treatment for severe atopic eczema in the TREAT survey, there has been a lot of interest in its use since two recent randomised controlled trials (RCTs) suggested no significant difference in efficacy between MTX and AZA (adults with severe atopic eczema, n=42) and MTX and CyA (children, n=40) (7, 8). However, both studies were statistically underpowered

(9). In addition, the paediatric trial lacked an intention-to-treat analysis and lower than conventional drug doses were used (CyA 2mg/kg/d; MTX 7.5mg/week, not adjusted by weight).

Cyclosporine A

Cyclosporine is a potent inhibitor of T-lymphocyte-dependent immune responses. A systematic review of 11 clinical trials suggested that it is an efficacious treatment but that relapse is often seen, certainly when it is used only for short bursts(10). The effectiveness of cyclosporine A was similar in children and adults, with good tolerability seen in younger patients with co-morbidities, even at the 5mg/kg/day dose (11). The open label RCT by Harper et al. suggested that more long-term treatment with CyA might result in disease remission, compared to short-term burst treatment (11), but none of the clinical trials included an observation period after treatment was stopped to assess the effect on natural history. The main potential side effects with more long-term use of CyA are increases in blood pressure due to nephrotoxicity, and regular blood pressure and renal function measurements are therefore important.

Azathioprine

Azathioprine inhibits purine synthesis and thus proliferation of leucocytes. The target cells and mechanism of action in atopic eczema are not fully elucidated(12). Azathioprine has a complex metabolism with several immunosuppressant metabolites. The balance between thiopurine metabolites is governed by thiopurine methyltransferase (TPMT) activity, and the pre-treatment determination of TPMT genotype or activity level allows informed drug dosing


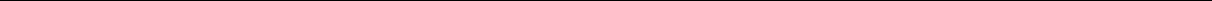


IRAS: 182752 15

TREAT Protocol v7.0 29/07/2020

**EudraCT Number:** 2015-002013-29

to minimise myelotoxicity. Other side effects include headache and gastrointestinal upset, hepatotoxicity and drug hypersensitivity. There is concern about the potential long-term risk of lymphoma based on observations in inflammatory bowel disease, but the risk increase seen may be related to inflammatory bowel disease itself rather than be drug-related(13). More recently, the emergence of progressive multifocal leukoencephalopathy (PML) in patients treated with azathioprine, either in combination with other immunomodulators, or as a single agent, has given further pause regarding this agent. A large scale ecological study of reported cases of PML in patients on immune suppression suggests that azathioprine appears to confer a significantly higher risk of PML compared to Cyclosporine (lower risk) or Methotrexate (minimal risk). These risks may be most relevant in the context of autoimmune disease and have not been reported in atopic dermatitis (14). Azathioprine has a slow onset of action, with clinical improvement sometimes only seen 8 weeks into therapy. Two double-blind, placebo controlled trials in adults with severe atopic eczema reported significant improvement in disease severity and quality of life. More recently, a RCT comparing azathioprine and methotrexate in adults with severe atopic eczema suggested comparable efficacy, but this trial (n=42) was not adequately powered to demonstrate equivalence in efficacy between the two drugs (8).

Methotrexate

As with azathioprine, the mechanism of action of methotrexate in atopic eczema is not fully understood, but it is known to have anti-inflammatory properties and to also reduce allergen-specific T cell activity(1). Gastrointestinal disturbance, in particular nausea, liver function abnormalities and bone-marrow suppression are potential side effects, but the medication is generally well tolerated in children and considered safe in the long-term, partly based on rheumatology experience in children and adults. Onset of action is equally slow as seen with azathioprine. Subcutaneous administration may improve bioavailability and tolerability in patients who have either failed to respond to treatment or who suffer significant gastrointestinal intolerance. In addition to the RCT that compared methotrexate with azathioprine in adults, there has been one recent RCT in children (n=40), comparing methotrexate with cyclosporine, also suggesting equal treatment responses (7).

The case for choosing CyA and MTX for the TREAT trial

Ciclosporin (CyA) is currently the only systemic immuno-suppressive medication licensed for the treatment of severe atopic eczema, and this only in patients ≥18 years of age and for a maximum of 8 weeks and is by far the most commonly used systemic agent in paediatric patients with recalcitrant atopic eczema. MTX is increasingly being used in the same patient group, partly because of the two small, underpowered RCTs mentioned above. There is therefore a clear need to compare methotrexate (MTX) with the most established immuno-suppressive medication, CyA. This has also been highlighted in a recent systematic review of oral immuno-modulatory medication in severe atopic eczema (15). Further support for research into the use of immuno-suppressive medication in severe atopic eczema in children was provided by a national research priority setting exercise run by the James Lind alliance in conjunction with the National Eczema Society in 2012, which involved patients, nurses and dermatologists, and listed the use of immune-suppressants in severe recalcitrant atopic eczema as one of the top five priority areas. This also specifically mentioned the need to compare MTX with more established immuno-suppressive therapies.

**2.2** **Rationale**

It is in particular the reported risk of rebound flares that warrants robust evaluation of CyA as a first line treatment approach against a comparator drug that is not only efficacious and safe but potentially also has the ability to alter the natural history of the disease. We propose that MTX fulfils all three requirements. As the anti-inflammatory action of MTX is poorly understood, this trial will include laboratory research that aims to shed further light on how


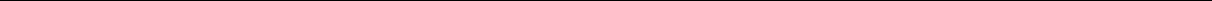


IRAS: 182752 16

TREAT Protocol v7.0 29/07/2020

**EudraCT Number:** 2015-002013-29

MTX achieves its anti-inflammatory effect with the potential to explain re-programming of the disease process.

**2.3 Objectives**

The main aim of this RCT is to assess the safety and efficacy (during therapy and to examine disease remission (time to first significant flare) after treatment cessation) of MTX versus CyA in severe recalcitrant atopic eczema in children.

In addition, we will examine the effect of both drugs on novel systemic and cutaneous markers of inflammation during treatment and after treatment cessation. Drug metabolites of MTX and trough levels of CyA will be measured throughout treatment to see whether they are able to predict treatment response and tolerability. Furthermore, filaggrin (FLG) genotype and T cell cytokine signatures will be determined in all trial participants to see whether these impact on treatment efficacy. We will also perform a health economic evaluation, comparing the cost effectiveness of both trial interventions.

**Primary objectives:**

1. To assess the change in atopic eczema severity between baseline & 12 weeks of treatment in the two treatment arms, and
2. To examine disease remission (time to first significant flare) after treatment cessation in the MTX vs CyA groups.

**Secondary objectives:**

1. To examine atopic eczema severity using the EASI, IGA, o-SCORAD & POEM scores between 0 and 12, 36, 48, 60 weeks,
2. To compare the number of flares in each study arm as well as the proportion of children who re-flared after treatment cessation,
3. To study the impact on quality of life: change in CDLQI/IDQOL & DFI scores between 0, 12, 36, 48 and 60 weeks,
4. To determine the proportion of participants achieving 50% improvement in the o-SCORAD and EASI index at 12, 36, 48, and 60 weeks,
5. To capture the proportion of participants who withdraw from treatment because of AEs,
6. To assess the cost-effectiveness of CyA vs MTX, based on utility measured using the CHU-9D,
7. To study the immuno-metabolic effects of MTX and CyA, especially in relation to markers of glycolytic activation and T cell cytokine signature, at baseline, during treatment and after treatment cessation,
8. To compare the drug side effects/toxicity profiles of both MTX and CyA, and
9. To examine the association between MTX polyglutamate and CyA trough levels and reduction in atopic eczema severity as well as drug-related side effects.
10. To study the impact of FLG carriage (yes/no) on reduction in atopic eczema severity.

**2.4** **Potential Risks and Benefits**

Patients with severe atopic eczema suffer significant sleep loss because of intractable generalised itching, commonly show poor school attendance and are often socially withdrawn. Attention-deficit hyperactivity disorder has recently been linked to severe atopic eczema, and these patients often go on to develop anxiety and depression(16, 17). Skin infections, in particular with *Staphylococcus aureus* but also herpes simplex, leading to hospital admissions are another typical feature (4, 5). The National Institute for Clinical Excellence guidelines for the treatment of children with severe eczema recommends systemic immuno-suppressive therapy with CyA or MTX(18). Indeed, both CyA and MTX have been used in severe inflammatory skin diseases for many years, including children with uncontrolled psoriasis and atopic eczema and are part of established NHS care and clinical


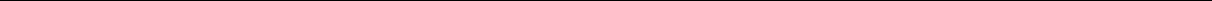


IRAS: 182752 17

TREAT Protocol v7.0 29/07/2020

**EudraCT Number:** 2015-002013-29

practice internationally (6). There are also American Academy of Dermatology treatment guidelines for systemic immuno-suppressive therapies in children with severe atopic eczema, which we adhere to in terms of treatment dose and safety monitoring. However, both treatments are used off label in this paediatric patient population, and patient safety is a key secondary outcome measure of the trial.

**2.4.1 Potential Risks:**

The use of CyA and MTX in children is seen in clinical practice for a large number of diseases and conditions. While malignant diseases, such as lymphoblastic leukaemia and lymphoblastic lymphoma are often treated using high doses, chronic inflammatory conditions, such as juvenile idiopathic arthritis, juvenile dermatomyositis, systemic lupus erythematosus, and scleroderma are treated using lower doses. For severe uncontrolled inflammatory skin diseases, such as psoriasis, typically a therapeutic dose of 0.4mg/kg/week MTX (max 25mg per week) is used(19), as indicated in the Children’s British National Formulary, the dose we are using in the TREAT trial. The Children’s British National Formulary indicates a therapeutic dose of 5mg/kg/day for CyA severe atopic eczema, while our trial protocol stipulates 4mg/kg/day, although treating TREAT trial physicians can go up to 5mg/kg/day, depending on treatment response.

The main potential side effects with the use of CyA are increases in blood pressure due to nephrotoxicity. Follow up of 42 paediatric patients with severe atopic eczema in a randomised controlled trial using continuous (n=21) vs repeated burst treatment (n=21) with 5mg/kg/day of CyA found no clinically significant change in mean serum creatinine or blood pressure over a 12 months period and tolerability was considered either ‘good’ or ‘very good’ in 80% of patients (11). The main reported side effects were: nausea (n=8), paraesthesia

(7), hypertrichosis (5), swollen gums (4), headache (4), rhinitis (3), upper respiratory tract infection (3), abdominal pain (3), and hyperuricaemia (11). The only RCT that used CyA (vs MTX) in children with severe atopic eczema found the following side effects over a 12 weeks treatment period: nausea/vomiting (2/20), glossitis/oral ulceration (1/20), diarrhoea (3/20), pancytopenia (3/20), anaemia (4/20), leukopenia (7/20), thrombocytopenia (2/20), abnormal liver profile (2/20), abnormal renal function (3/20), and hypertension (1/20). Regular blood pressure and renal function measurements are the main safety investigations in the CyA arm of the study. To provide an assessment of renal function above and beyond standard NHS care, we are not only determining plasma creatinine but also cystatin C levels (baseline, 2 weeks, 8 weeks, 12 weeks, 36 weeks and 60 weeks) as well as urinary tubular N-acetyl-beta-D-glucosaminidase (at baseline, 2 weeks, 12 weeks, 36 weeks and 60 weeks).

(11).

As for MTX, gastrointestinal disturbance, in particular nausea, liver function abnormalities and bone-marrow suppression are the main potential side effects, but the medication is well tolerated in the majority of cases and considered safe in the long-term, partly based on the extensive rheumatology and gastroenterology experience in children, using similar if not higher doses for longer periods of time and often in combination with biologics(4, 5, 20-23) . In addition, the only RCT using MTX in children with severe atopic eczema over a 12 week period, the following adverse events were recorded: nausea/vomiting (4/20), abdominal pain (1/20), glossitis/oral ulceration (4/20), diarrhoea (5/20), pancytopenia (1/20), anaemia (6/20), leukopenia (2/20), abnormal liver function (5/20), abnormal renal function (1/20), fatigue (6/20), headache (3/20), and flu-like symptoms (1/20). This overall favourable side effect profile has been confirmed by three observational studies, involving a total of 102 paediatric patients with treatment durations of up to 38 months (24-26). We are performing safety bloods after the initial test dose for MTX to capture rare idiosyncratic reactions early and review participants in both study arms with safety bloods and enquiry about adverse events fortnightly for the first month, then monthly until week 12 and then eight-weekly, while on treatment, in keeping with the American Academy of Dermatology guidelines for the use of these systemic agents in children with severe atopic eczema(27). Patients are also encouraged to contact the study team in case of any concerns about drug adverse reactions


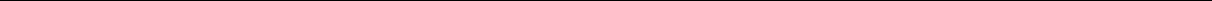


IRAS: 182752 18

TREAT Protocol v7.0 29/07/2020

**EudraCT Number:** 2015-002013-29

between study visits. In addition, all potential participants and their parents will be informed about common and rare serious side effects in the Patient Information Leaflets and also instructed to contact the study team/seek medical advice straight away if any of the following occur:

Methotrexate:

Infections, including fever (temperature above 38^o^C), chills, sore throat or chicken pox

Skin rash, changes in nail or skin colour, or skin ulcers Jaundice

Bleeding gums, unexpected bruising or bleeding that doesn’t stop as quickly as normal

Melaena

Chest pain, difficulty breathing or chronic dry cough

Severe and continuing diarrhoea, vomiting or stomach pains

Ciclosporin:

Infections, including fever (temperature above 38^o^C), chills, sore throat or chicken pox

Unusual bleeding that is difficult to stop Severe bruising

Headaches and associated visual disturbance

**2.4.2 Known Potential Benefits**

Both drugs have been demonstrated to reduce atopic eczema severity and improve quality of life (4, 5) However, there is equipoise with regard to which of the treatments is superior in terms of short-term efficacy (first primary outcome looks at the effectiveness of CyA vs MTX at 12 weeks) and more long-term disease control (secondary outcome is disease control during the six months follow up period).

Ciclosporin:

A systematic review of 11 clinical trials suggested that CyA is an efficacious treatment but that relapse is often seen, certainly when it is used only for short bursts of 2-3 months.^i^ The effectiveness of CyA was similar in children and adults, with good tolerability seen in younger patients with co-morbidities, even at the 5mg/kg/day dose (11). The open label RCT by Harper et al. suggested that more long-term treatment with CyA might result in disease remission, compared to short-term burst treatment(4), but none of the clinical trials included an observation period after treatment was stopped to assess the effect on natural history.

Methotrexate:

Although the evidence base for MTX is smaller than for CyA in severe atopic eczema, the RCT that compared methotrexate with azathioprine in adults (n=42, MTX vs AZA) (8), and the RCT in children (n=40, MTX vs CyA) (7) clearly demonstrated its positive effect on disease severity and quality of life, also underpinned by the three observational studies already discussed above.


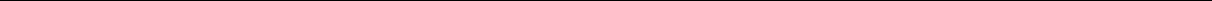


IRAS: 182752 19

TREAT Protocol v7.0 29/07/2020

**EudraCT Number:** 2015-002013-29

**3** **SELECTION OF CENTRES/CLINICIANS**

Study centres will be initiated once all global (e.g. local R&D approval) and study-specific conditions (e.g. training requirements) have been met, and all necessary documents have been returned to CTRC. Initiation meetings will cover the requirements outlined in CTRC and KHP CTO SOPs.

**3.1 Centre/Clinician inclusion criteria**

Each participating centre (and Principal Investigator; PI) has been identified on the basis of:

Having at least one consultant dermatologist with a specific interest in, and responsibility for supervision and management of patients with severe atopic eczema

Showing enthusiasm to participate in the study

Ensuring that sufficient time, staff and adequate facilities are available for the trial Providing information to all supporting staff members involved with the trial or with

other elements of patient management

Identifying that they will be able to recruit the required number of patients Acknowledging and agreeing to conform to the administrative and ethical

requirements and responsibility of the study including adhering to GCP and other regulatory documentation

Other important criteria are:

- 1. Local R&D approval/ Confirmation of capacity and capability
  2. Completion and return of ‘Signature and Delegation Log’ to CTRC
  3. Signed contract between site and sponsor
  4. CTRC and KHP CTO green light process criteria are met

**3.2 Centre/Clinician Exclusion Criteria**

a. Not meeting the inclusion criteria listed above


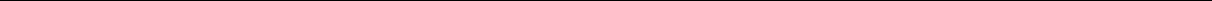


IRAS: 182752 20

TREAT Protocol v7.0 29/07/2020

**EudraCT Number:** 2015-002013-29

**4 TRIAL DESIGN**

This study is a phase III, multi-centre randomised trial comparing methotrexate and ciclosporin. As per protocol trial treatment duration is for 36 weeks. Participants remain in the study for a total of 60 weeks post randomisation.

Participants will be randomised applying a ratio of 1:1.


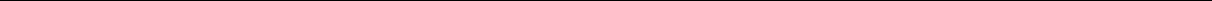


IRAS: 182752 21

TREAT Protocol v7.0 29/07/2020

**EudraCT Number:** 2015-002013-29

**5 STUDY POPULATION**

**5.1** **Inclusion Criteria**

1. Written informed consent for study participation obtained from the patient or parents / legal guardian, with assent as appropriate by the patient, depending on the level of understanding.
2. Aged 2-16 years at the time of the screening and randomisation visit
3. Diagnosis of severe recalcitrant atopic eczema
4. History of inadequate clinical response (in the opinion of the treating clinician) to potent topical corticosteroids on the body and moderate strength topical corticosteroids on the face.
5. An objective (o)-SCORAD severity score of at least 30
6. Participants must live within travelling distance of the recruiting centre
7. Females of childbearing potential and males, who are sexually active, must commit to consistent and correct use of an highly effective method of contraception (e.g. combined hormonal contraception, intrauterine device, physical barrier or abstinence) for the duration of the trial and for 6 months after the last dose of study drug.
   1. Females of childbearing potential for this study are: Females, regardless of their age, with functioning ovaries and no documented impairment of oviductal or uterine function that would cause sterility. This category includes young females who have begun to menstruate, or females with oligomenorrhea.
8. Willingness to comply with study requirements
9. Baseline visit within 2 weeks of the screening visit

**5.2** **Exclusion Criteria**

1. Serious underlying medical condition which in the opinion of the Investigator would compromise the safety of the patient.
2. Pregnant or nursing (lactating) females, where pregnancy is defined as the state of a female after conception and until the termination of gestation, confirmed by a positive hCG laboratory test.
3. Any active and/or chronic infection at screening or baseline (randomisation) visit that, based on the investigator's clinical assessment, makes the subject an unsuitable candidate for the study.
4. Presence of moderate to severe impaired renal function as indicated by clinically significantly abnormal creatinine (≥ 1.5 x upper normal limit (ULN) for age and sex) AND eGFR <60ml/min/1.73m^2^ at screening visit.*
5. Clinical evidence of liver disease or liver injury at screening visit as indicated by abnormal liver function tests such as AST, ALT, GGT, alkaline phosphatase, or serum bilirubin (must not exceed 1.5 x the upper limit value of the normal range for age and sex).
6. Total WBC count <3x10^9^/L, or platelets <150x10^9^/L or neutrophils <1.5x10^9^/L or haemoglobin <8.5 g/L at screening visit.
7. Blood pressure values > 95^th^ percentile for age and sex at screening *and* baseline visit. **
8. Received systemic cortico-steroids within 14 days prior to screening visit and 28 days of baseline visit.
9. Received phototherapy within 4 weeks prior to screening visit and 6 weeks of the baseline visit.
10. Previous exposure to any biologic agents or systemic immuno-suppressive therapy, except for oral corticosteroids (CS) for acute flare management.
11. Concomitant use of disease-modifying and/or immunosuppressive drugs.
12. Received live vaccines within 4 weeks prior to baseline visit.


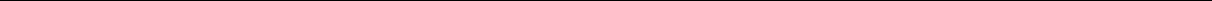


IRAS: 182752 22

TREAT Protocol v7.0 29/07/2020

**EudraCT Number:** 2015-002013-29

1. Radiology report of abnormal chest x-ray at the screening visit (at the discretion of the PI/medically qualified physician - see section 7.1 for further details)
2. Receiving treatment with medicines that are substrates for the multidrug efflux transporter P-glycoprotein or the organic anion transporter proteins (OATP) for which elevated plasma concentrations are associated with serious and/or life-threatening events; this includes bosentan, dabigatran etexilate and aliskiren.
3. Receiving treatment with products containing *Hypericum perforatum* (St. John's wort)
4. Receiving oral treatment with tacrolimus
5. Receiving oral treatment with everolimus and sirolimus
6. Receiving oral treatment with lercanidipine
7. Currently participating in a conflicting study or participation in a clinical study involving a medicinal product in the last 28 days or less than 5 half-lives of the medicinal product prior to the screening visit.
8. Known hypersensitivity to methotrexate or ciclosporin products.
9. Insufficient understanding of the trial by the patient and/or parent/guardian.

*Please ensure the following formula is used for calculating eGFR: eGFR = height (cm) x 40/plasma creatinine (micromol/l)

- If an accurate blood pressure reading is unobtainable (e.g. due the patient being distressed), the patient’s GP may be approached in order to obtain an accurate reading if a blood pressure measurement has been carried out within 4 weeks of the screening and baseline visit. A community nurse may also carry out a blood pressure measurement at the patient’s home to confirm eligibility and also during the follow up visits. Without an accurate blood pressure measurement below the 95^th^ centile of systolic BP for age and sex, a patient cannot be randomised into the trial.


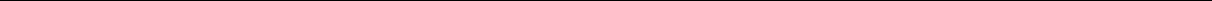


IRAS: 182752 23

TREAT Protocol v7.0 29/07/2020

**EudraCT Number:** 2015-002013-29

**6 ENROLMENT, RANDOMISATION AND WITHDRAWAL**

**6.1** **Recruitment and Screening**

Participants will be identified by the clinical team at each centre via a search of the patient database/s either electronically or manually or clinic list review to find potentially eligible patients. At the routine clinic visit, the patient and/or parent/guardian will be asked whether they would be willing to participate in the study. A Patient Information Sheet and instructions on how to proceed if they are interested in taking part or finding out more about the study will be given to the patient and/or parent/guardian. They will be provided with a telephone number to contact the research nurse if they need to discuss or need further information. All patients and/or parent/guardian will be provided with a full explanation of the trial before obtaining informed written consent (see Section 11.3 for the consent procedure) at the screening visit.

The time taken from initial contact and provision of information to obtaining written consent should be sufficient to enable appropriate discussions with the patient / family about the trial, explanation of the protocol and procedures, and seeking formal consent.

A patient and/or parent/legal guardian should be provided with the study information during their outpatient appointment and may be approached for consent during this visit if considered appropriate. Where the patient and/or parent/legal guardian feel that they have adequate information regarding the trial and are happy to proceed then consent can be sought and screening assessments undertaken on the same day as initial contact and provision of information.

A ‘Consent & Eligibility Log’ will be maintained to record all the patients who attend dermatology clinics that have severe recalcitrant atopic eczema (in the opinion of the treating clinician). Patients will be recorded on the log regardless of whether they decide to participate in the study or are found ineligible to participate. Reasons for not being eligible will be recorded on the log. Reasons for declining to participate will be asked routinely but it will be made clear that they do not have to provide a reason unless happy to do so. The participating site will assign the patient a screening number for use on the study documentation, until randomisation number is allocated.

**6.2** **Screening Visit**

The screening visit of potentially eligible participants will take place following written informed consent to participate. Informed consent can also be taken at or prior to the screening visit, just prior to assessments. If taken prior to the screening visit, then the screening visit should occur within **14 days** of informed consent. A copy of the consent and assent forms should be submitted to CTRC within 7 days of the screening visit. Data from clinical assessments undertaken as part of routine care prior to obtaining consent can be used (i.e. safety bloods, o-SCORAD) for assessing eligibility as long as screening and or/baseline visits are within the specified time frame (section 6.2 & 6.3).

The visit will include:

1. Written informed consent will be obtained from parent/legal guardian or 16 year old patient (or verification of this if obtained previous to the visit)
2. full medical history
3. assessment of eligibility criteria
4. review of concomitant medication
5. pregnancy test where indicated
6. height


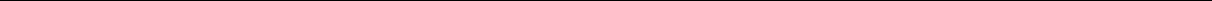


IRAS: 182752 24

TREAT Protocol v7.0 29/07/2020

**EudraCT Number:** 2015-002013-29

1. safety bloods
2. collection of demographic data
3. completion of o-SCORAD. An Excel calculator will be provided to calculate the score. The completed tool and the CRF must be returned to the CTRC. Please note, if an o-SCORAD assessment was carried out prior to the screening visit for a clinical reason, this data can be used if the assessment was completed within 14 days of the screening visit. However, a new o-SCORAD assessment must be carried out for eligibility at the baseline visit.
4. Chest X-ray (at the discretion of the local PI/medically qualified physician)

Please note patients who fail screening can be invited for re-screening after 14 days, if it is appropriate to do so.

**6.3 Baseline**

Baseline visit should be within 2 weeks of the screening visit. However, if safety bloods have been collected within 14 days prior to the baseline visit for a clinical reason, then the screening and baseline visit can be carried out on the same day. In this instance, please ensure both screening and baseline CRFs are completed. **Please note an additional pregnancy test will be required if the screening visit and baseline visit have been completed on separate days to ensure that the patient is eligible at the time of randomisation.**

Baseline visit procedure:

1. confirm Informed Consent status
2. assess eligibility
3. review of concomitant medication
4. pregnancy test where indicated
5. clinician to conduct physical exam
6. blinded assessor to conduct o-SCORAD, EASI and IGA (o-SCORAD calculator provided to the participating sites)
7. patient randomised
8. IMP dispensed
9. parent and child to complete QoL questionnaires (during the visit)
10. height, weight, BP, urine sample collection
11. collection of blood samples and skin tape strips for mechanistic work (tape strips not collected at all sites)
12. collection of blood/saliva for FLG genotyping
13. Parent/patient to complete POEM baseline questionnairepatient diary (including POEM) given to patient/parent to complete at home

All eligibility assessments must be reviewed, and full eligibility confirmed by a **medically qualified physician** authorised to do so on the Delegation Log. This must be explicitly recorded in the medical notes **prior to randomisation**.


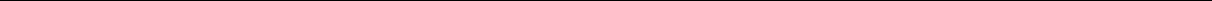


IRAS: 182752 25

TREAT Protocol v7.0 29/07/2020

**EudraCT Number:** 2015-002013-29

**6.4** **Randomisation and blinding**

**6.4.1 Randomisation**

Participants will be randomised to receive MTX or CyA in a 1:1 ratio at the Baseline Visit once:

1. Eligibility criteria have been fulfilled;
2. Fully informed written consent has been obtained;
3. Baseline assessments have been completed

Participants will be randomised using a secure (24-hour) web based randomisation programme controlled centrally by CTRC.


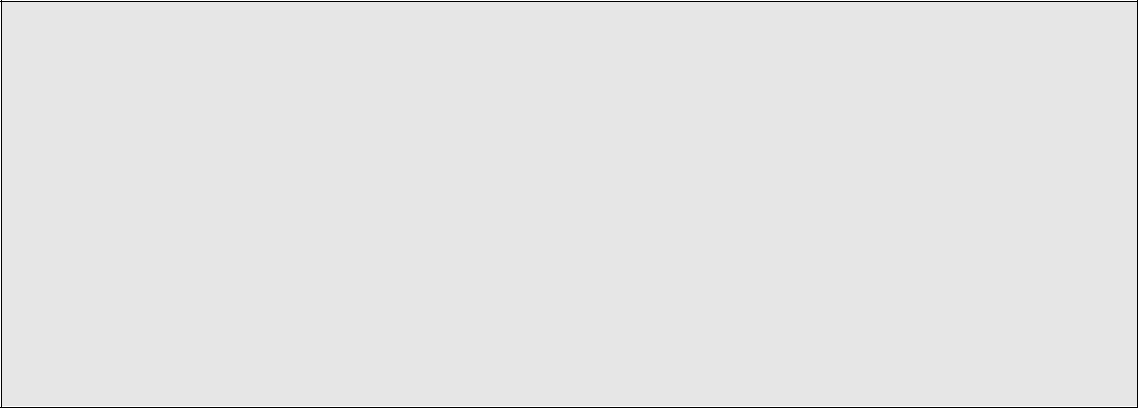


**Randomisation web access:** <https://ctrc.liv.ac.uk/Randomisation/Treat>

*If there are problems with web randomisation, please contact the trial coordinator*

(Note that the CTRC is open from 0900 – 1700, Monday – Friday, excluding public holidays)

*Randomisation backup envelopes will be used in case of failure of the randomisation systems outside CTRC working hours.*

Research staff will be trained to use the randomisation system and will be added to the trial delegation log and authorised to carry out this role. Following this (and the green light for their site), they will be issued with usernames and passwords to access the system.

In the event of a randomisation system failure, the centre should contact the trial coordinator at CTRC (Monday to Friday between 9:00 to 17:00 excluding bank holidays) to try to resolve the problem.

Centres will be provided with emergency back-up randomisation envelopes to be used in the event of a system failure that occurs outside CTRC office hours or when a system failure cannot be resolved in a reasonable timeframe. In the event that emergency back-up envelopes are required, the randomising person will select the next sequentially numbered, opaque, pressure-sealed envelope that will give the randomisation allocation. The envelope will be similar to those used for pay slips, which cannot be viewed without fully opening and their construction is resistant to accidental damage or tampering. Page 1 of the randomisation envelope containing information on the allocation should be returned to the coordinating centre in a pre-paid envelope, and pages 2 & 3 of the randomisation envelope should be inserted into the patient’s medical records.

The research staff will check to ensure that the correct number of randomisation envelopes is present, that they are intact and that the sequential numbering system is maintained. Any discrepancies should be immediately reported to the CTRC.


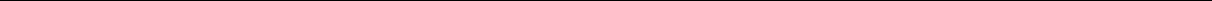


IRAS: 182752 26

TREAT Protocol v7.0 29/07/2020

**EudraCT Number:** 2015-002013-29

**6.4.2 Blinding**

As the trial interventions are at different frequencies (daily vs once weekly), have rather different side effect profiles and since no placebo is used as part of the study, blinding of the local investigator and research nurse will not be possible. However, the assessor who will perform the severity assessments (o-SCORAD, EASI & IGA), will be blinded to the trial allocation. The following steps are in place to ensure blinding is maintained:

-All severity assessments will be conducted by an independent member of the research team, who has no direct involvement or other contact with the trial participants.

- Participants will be reminded in their study visits not to mention the treatment they are on to the independent member of the research team carrying out the assessments.
- The blinded assessor will wear a badge at the severity assessment, reminding participants not to mention what treatment they are on.
- All questions relating to the acceptability and use of the trial drugs will be completed in a patient diary that will be returned to the study research nurse.
- Dispensing of study drugs will be completely independent from the blinded assessor.

Following each severity assessment, the blinded assessor will be asked whether they had become unblinded to the allocation and this information will be recorded in the Case Report Form. If the blinded assessor does become unblinded, this will be used to inform a sensitivity analysis.

Whilst it will not be possible to blind participants to their treatment allocation either, efforts will be made to minimise expectation bias by emphasising in the trial literature that there is currently no strong evidence favouring either drug choice in severe atopic eczema in children.

**6.5 Patient Transfer and Withdrawal**

Follow-up of patients who transfer or withdraw will be continued through the trial Research Nurses and the lead investigator at each centre, unless the participant explicitly also withdraws consent for follow-up.

**6.5.1 Patient Transfers**

For patients moving from the area, every effort should be made for the patient to be followed-up at another participating trial centre and for this trial centre to take over responsibility for the patient.

A copy of the patient CRFs should be provided to the new site. The patient (or parent/legal representative) will have to sign a new consent form at the new site, and until this occurs, the patient remains the responsibility of the original centre. The CTRC should be notified in writing of patient transfers.

**6.5.2 Discontinuation from Trial Intervention**

Patients will be discontinued from treatment for any of the following reasons:

1. Parent/ legal representative (or, where applicable, the patient) withdraws consent.
2. Unacceptable toxicity based on investigator’s judgement
3. Development of illness preventing further treatment.
4. Any change in the patient’s condition that justifies the discontinuation of treatment in the clinician’s opinion.
5. Patient cannot be monitored as per protocol

If a patient wishes to discontinue from trial treatment, centres should nevertheless explain the importance of remaining on trial follow-up, or failing this, of allowing routine follow-up


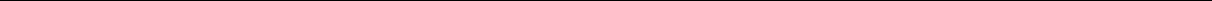


IRAS: 182752 27

TREAT Protocol v7.0 29/07/2020

**EudraCT Number:** 2015-002013-29

data to be used for trial purposes. Generally, follow-up will continue unless the patient explicitly also withdraws consent for follow-up (see section 6.5.3).

Should a trial participant be administered one of the trial treatments (ciclosporin or methotrexate) as part of routine clinical care following cessation of their trial treatment then safety reporting procedures should be followed as described in section 10.

Data collected from participants who discontinue from trial treatment prior to week 36, will differ from those still on trial treatment as the collection of trial data and samples will be reduced. If the participant is administered one of the trial treatments (ciclosporin or methotrexate) as part of routine clinical care following cessation of their trial treatment (prior to week 36), information relating to drug monitoring should not be recorded on the CRFs (except for on the concomitant medication form).

The following data **should only** be collected for participants who have been discontinued from trial treatment between randomisation and week 36:

**Table 1. Schedule of study procedures (patients discontinued from trial treatment)**

|  | Week | Week | Week | Week | Week | Week | Week | Week | Week | Week | Unscheduled |
| --- | --- | --- | --- | --- | --- | --- | --- | --- | --- | --- | --- |
|  | 1 | 2 | 4 | 8 | 12 | 20 | 28 | 36 | 48 | 60 | visit |
| Procedures | Visit | Visit | Visit | Visit | Visit | Visit | Visit | Visit | Visit | Visit |  |
|  | 1 | 2 | 3 | 4 | 5 | 6 | 7 | 8 | 9 | 10 |  |
| Concomitant | **x** | **x** | **x** | **x** | **x** | **x** | **x** | **x** | **x** | **x** | x |
| drugs |  |  |  |  |  |  |  |  |  |  |  |
|  |  |  | **x** | **x** | **x** | **x** | **x** | **x** | **x** | **x** | X (if |
| o-SCORAD) |  |  |  |  |  |  |  |  |  |  | required) |
|  |  |  | **x** | **x** | **x** | **x** | **x** | **x** | **x** | **x** | X (if |
| EASI, IGA |  |  |  |  |  |  |  |  |  |  | required) |
| POEM (patient |  |  | **x** | **x** | **x** | **x** | **x** | **x** | **x** | **x** |  |
| assessed) |  |  |  |  |  |  |  |  |  |  |  |
| (Post 36 week |  |  |  |  |  |  |  |  |  |  |  |
| patient diary) |  |  |  |  |  |  |  |  |  |  |  |
| Parent and |  |  |  |  | **x** |  |  | **x** | **x** | **x** |  |
| child QoL |  |  |  |  |  |  |  |  |  |  |  |
| (CDLQI/IDQOL |  |  |  |  |  |  |  |  |  |  |  |
| & DFI) |  |  |  |  |  |  |  |  |  |  |  |
| Child HRQL |  |  |  |  | **x** |  |  | **x** | **x** | **x** |  |
| (CHU-9D) |  |  |  |  |  |  |  |  |  |  |  |
| Resource Use |  |  | **x** | **x** | **x** | **x** | **x** | **x** | **x** | **x** |  |
| (post 36 week |  |  |  |  |  |  |  |  |  |  |  |
| patient diary) |  |  |  |  |  |  |  |  |  |  |  |
| Adverse | Collected until 4 weeks after trial treatment stopped | | | | | | |  |  |  |  |
| events (AE & |  |  |  |  |  |  |  |  |  |  |  |
| SAE) |  |  |  |  |  |  |  |  |  |  |  |
| Confirmation of | **x** | **x** | **x** | **x** | **x** | **x** | **x** | **x** | **x** | **x** | x |
| appropriate |  |  |  |  |  |  |  |  |  |  |  |
| contraception |  |  |  |  |  |  |  |  |  |  |  |
| use, where |  |  |  |  |  |  |  |  |  |  |  |
| applicable |  |  |  |  |  |  |  |  |  |  |  |


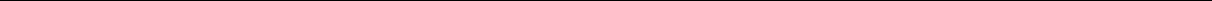


IRAS: 182752 28

TREAT Protocol v7.0 29/07/2020

**EudraCT Number:** 2015-002013-29

**6.5.3 Withdrawal from Trial Completely**

Patients are free to withdraw consent at any time without providing a reason. In consenting to the trial, patients are consented to trial treatment, trial procedures, follow-up phase after treatment cessation and data collection (including sample collection). If voluntary withdrawal occurs, the patient (or parent/legal representative, where applicable) should be asked to allow continuation of scheduled evaluations, complete an end-of-study evaluation and be given appropriate care under medical supervision until the symptoms of any adverse event resolve or the subject’s condition becomes stable. If consent is withdrawn completely then the reasons for withdrawal of consent will be collected (if possible) and reported for both groups. Participants who wish to withdraw consent for the trial will have anonymised data collected up to the point of that withdrawal of consent included in the analyses unless the patient explicitly states that this is not their wish. The patient will not contribute further data to the study, and the CTRC should be informed in writing by the responsible physician and a withdrawal CRF should be completed. The CTRC should notify laboratories that are in possession of samples that belong to patients who have withdrawn and requested that their data should not be analysed and should be destroyed. The destruction of the samples will be adequately documented.


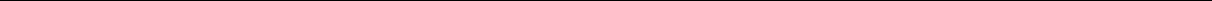


IRAS: 182752 29

TREAT Protocol v7.0 29/07/2020

**EudraCT Number:** 2015-002013-29

**7 TRIAL TREATMENTS**

**7.1 Introduction**

**Table 2:Investigational Medicinal Products (IMPS) and Approved Formulations**

| **Arm** | **IMPs** | **Formulations** |
| --- | --- | --- |
| **CyA** | Ciclosporin | Brand: Neoral |
|  |  | Capsules: 10mg, 25mg, 50mg, 100mg |
|  |  |  |
|  | Ciclosporin | Brand: Neoral |
|  |  | Oral solution: 100mg/ml |
|  |  |  |
|  | Methotrexate | Brand: any brand with marketing authorisation within EEA |
|  |  | Tablets: 2.5mg |
| **MTX** |  |  |
|  | Methotrexate | Brand: any brand with marketing authorisation within EEA |
|  |  |  |
|  |  | Injection: 25mg/ml, 50mg/ml prefilled pen |
|  |  |  |
|  | Methotrexate | Brand: any brand with marketing authorisation within EEA |
|  |  | Oral solution: 2mg/ml |
|  |  |  |

Please note that folic acid (for those randomised to methotrexate) is classed as non-investigational medicinal product (NIMP) in this trial. The product should be dispensed by pharmacies in accordance to standard clinical practice.

CYA and MTX oral liquid preparations are available as licensed products in the UK. In the TREAT trial, these oral liquid preparations are available for children who are unable to swallow tablets/capsules or express a preference for the oral solution.

The provision of both IMPs and NIMPs is the responsibility of each individual participating site in accordance with standard purchasing arrangement. Both IMPs and NIMPs used in this trial are not provided free of charge from the sponsor, as they are considered an NHS treatment cost.

The drug dosing for MTX and CyA as well as the frequency of the study visits and safety assessments, including safety bloods, are in keeping with SmPC guidance and the American Academy of Dermatology guidelines for the use of systemic immuno-suppressive therapy in children and young people with severe atopic eczema (27) (see Section 80, page 39).

Also in keeping with the SmPC guidance, as a safety measure some patients may require a chest x-ray at the screening visit at the discretion of the Principal

Investigator or a medically qualified physician. Such patients are those who are at high risk of tuberculosis (TB) (recent travel to a country where TB is common or have been in contact with someone who has active TB). The patient will need to have a radiology report of clear/normal chest X-ray, before randomisation occurs. If the patient has an abnormal radiology report they will not be eligible to be randomised. The radiation dose from the chest X-ray is equivalent to between 2 - 8 days of natural background radiation and is of negligible risk.

Countries where TB is common can be found at the following link: <https://www.gov.uk/tb-test-visa/countries-where-you-need-a-tb-test-to-enter-the-uk>


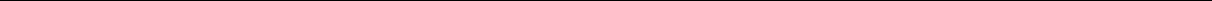


IRAS: 182752 30

TREAT Protocol v7.0 29/07/2020

**EudraCT Number:** 2015-002013-29

**7.2 Arm A - Ciclosporin**

**7.2.1 Ciclosporin - Formulation, Packaging, Labelling, Storage and Stability**

**Table 3: Ciclosporin approved formulation**

| **Arm** | **IMP** | **Formulation** |
| --- | --- | --- |
| **CyA** | Ciclosporin | Brand: Neoral |
|  |  | Capsules: 10mg, 25mg, 50mg, 100mg |
|  |  |  |
|  | Ciclosporin | Brand: Neoral |
|  |  | Oral solution: 100mg/ml |
|  |  |  |

The Sponsor will provide Medicines & Healthcare products Regulatory Agency (MHRA) & Health Products Regulatory Authority (HPRA) approved regulatory compliant IMP labels for the trial; these should be kept in the Pharmacy File until the point of dispensing. Please contact the CTRC for further supplies of trial labels or request a pdf file of the label for local printing in an appropriate label format. The subject randomisation number and name of investigator should be recorded on the trial label at dispensing.

Refer to the relevant SmPC for information on storage requirements.

Temperature monitoring should be in line with local requirements for general medicine supplies held in Pharmacy.

**7.2.2** **Preparation, Dosage and Administration of Study Treatment/s**

**Table 4: Ciclosporin capsules**

|  | **Ciclosporin oral solution/capsules -** Neoral brand only |
| --- | --- |
| **Dose** | 2mg/kg (total: 4mg/kg/day) |
|  | (rounded to the nearest whole capsule where applicable) |
| **Frequency** | Twice daily |
|  |  |
| **Duration** | 36 weeks |
|  |  |
| **Route** | Oral |
|  |  |
| **Notes** | Advise participants to avoid grapefruit juice |
|  |  |

**7.2.3 Ciclosporin - Dose Modifications**

Patients should remain on the full treatment dose (4mg/kg/day for CyA) for the 12 weeks. After that, dose increases to a maximum of 5mg/kg/day or dose decreases are allowed, according to the treatment response. For decisions on treatment continuation, PIs should refer to the table below. For situations not included in the below table, this is down to the individual study centre PI and any dose changes will be recorded in the CRF.


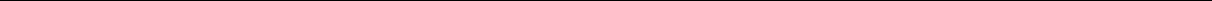


IRAS: 182752 31

TREAT Protocol v7.0 29/07/2020

**EudraCT Number:** 2015-002013-29

**Table 5: Dose modification schedule**

***Note:*** *any events that meet the monitoring parameters in the table below should be recorded and reported as an adverse event (see section 10.7)*

| **Monitoring parameters** | **Values** | **Action** |
| --- | --- | --- |
| eGFR | Fall of >=20% (compared with | Repeat renal profile should be |
|  | eGFR value that was used to | done within 2 weeks of the last |
|  | confirm eligibility) | visit. If eGFR falls again >=20 |
|  |  | % following repeat bloods, the |
|  |  | trial nephrologist must be |
|  |  | contacted to discuss potential |
|  |  | dose reduction. If repeat bloods |
|  |  | cannot be obtained within 2 |
|  |  | weeks of the last visit, the trial |
|  |  | nephrologist must be contacted |
|  |  | within 48 hours of the site |
|  |  | becoming aware that a repeat |
|  |  | renal profile cannot be obtained |
|  |  | within the timeframe (2 weeks), |
|  |  | to discuss potential dose |
|  |  | reduction. |
| Blood pressure | >95^th^ centile for age and sex on | CyA dose adjustment, |
|  | two consecutive visits | reduction by at least 20% |
|  |  | initially and patient review. A |
|  |  | temporary dose suspension at |
|  |  | the PI’s discretion is permitted. |
|  |  | Repeat BP after a fortnight. |
| Liver function test | AST, ALT or alkaline phosphatase | CyA dose adjustment reduction |
|  | more than 2× upper limit of | by at least 20% initially or a |
|  | reference range | temporary dose suspension at |
|  |  | the PI’s discretion. |
|  |  | Repeat LFT weekly until |
|  |  | normal. Further reductions in |
|  |  | dose or stopping medication |
|  |  | may be required but should be |
|  |  | discussed with the Chief |
|  |  | Investigator. |
| Platelet count | <100x10^9^/L | CyA dose adjustment reduction |
|  |  | by at least 20% initially or a |
|  |  | temporary dose suspension at |
|  |  | the PI’s discretion. |
|  |  | Repeat platelets weekly until |
|  |  | normal. Further reductions in |
|  |  | dose or stopping medication |
|  |  | may be required but should be |
|  |  | discussed with the Chief |
|  |  | Investigator. |
| Neutrophil count | <1.5x10^9^/L | CyA dose adjustment reduction |
|  |  | by at least 20% initially or a |
|  |  | temporary dose suspension at |
|  |  | the PI’s discretion. |
|  |  | Repeat neutrophils weekly until |
|  |  | normal. Further reductions in |
|  |  | dose or stopping medication |


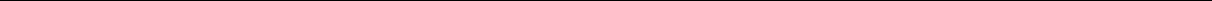


IRAS: 182752 32

TREAT Protocol v7.0 29/07/2020

**EudraCT Number:** 2015-002013-29

|  |  | may be required but should be |
| --- | --- | --- |
|  |  | discussed with the Chief |
|  |  | Investigator. |
|  |  |  |
| Unexplained bruising, | Not applicable | Review by the PI prior |
| chicken pox contact or |  | to continuing with CyA |
| rash suspected to be |  |  |
| chicken pox infection |  |  |

Treatments may be suspended for clinical reasons. If for any reason a patient misses a treatment dose, this will be documented in the patient diary but the patient should not discontinue trial treatment or be withdrawn from the trial treatment. Patients who discontinue trial treatment should be asked to continue with study assessments. See section 6.5.2.

Occasional monitoring of ciclosporin blood levels is recommended e.g. when Neoral is co-administered with medicines that are known to interact with ciclosporin or where the patient is not responding to the therapy. This will be assessed by the local investigator on a per patient basis and put in place where deemed clinically appropriate.

**7.3 Arm B - Methotrexate**

**7.3.1 Methotrexate - Formulation, Packaging, Labelling, Storage and Stability**

**Table 6: Methotrexate approved formulations**

| **Arm** | **IMPs** | **Formulations** |
| --- | --- | --- |
|  | Methotrexate | Brand: any brand with marketing authorisation within EEA |
|  |  | Tablets: 2.5mg |
| **MTX** |  |  |
|  | Methotrexate | Brand: any brand with marketing authorisation within EEA |
|  |  |  |
|  |  | Injection: 25mg/ml, 50mg/ml prefilled pen |
|  |  |  |
|  | Methotrexate | Brand: any brand with marketing authorisation within EEA |
|  |  | Oral solution : 2mg/ml |
|  |  |  |

The Sponsor will provide MHRA & HPRA approved regulatory compliant IMP labels for the trial; these should be kept in the Pharmacy File until the point of dispensing. Please contact the CTRC for further supplies of trial labels or request a pdf file of the label for local printing in an appropriate label format. The subject trial number and name of investigator should be recorded on the trial label at dispensing.

Refer to the relevant SmPC for information on storage requirements for methotrexate tablets.

Temperature monitoring should be in line with local requirements for general medicine supplies held in Pharmacy.

**7.3.2 Methotrexate - Preparation, Dosage and Administration of Study Treatment/s**

**Table 7: Methotrexate tablets/ subcutaneous injection/oral solution**

**
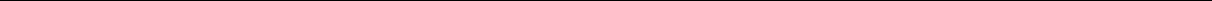
**

IRAS: 182752 33

TREAT Protocol v7.0 29/07/2020

**EudraCT Number:** 2015-002013-29

|  | **Methotrexate tablets / subcutaneous injection/ oral solution** | | | | |
| --- | --- | --- | --- | --- | --- |
|  |  | | | | |
| **Dose** | Initial dose of 0.1mg/kg/week, then 0.4mg/kg/week from week 1 (maximum | | | | |
|  | 25mg/week) | |  |  |  |
|  | For patients ≥ 22kg, doses should be rounded as per table below irrespective | | | | |
|  | of formulation. | |  |  |  |
|  |  |  |  |  |  |
|  |  | **Weight band** | **0.1mg/kg test** | **0.4mg/kg dose** |  |
|  |  |  | **dose** |  |  |
|  |  | <22kg | no rounding required | |  |
|  |  | 22 to <29 kg | 2.5mg | 10mg |  |
|  |  | 29 to <36 kg | 2.5mg | 12.5mg |  |
|  |  | 36 to <43 kg | 5mg | 15mg |  |
|  |  | 43 to <50 kg | 5mg | 17.5mg |  |
|  |  | 50 to <57 kg | 5mg | 20mg |  |
|  |  | 57 to <63kg | 5mg | 22.5mg |  |
|  |  | 63kg + | 7.5mg | 25mg |  |
|  | Methotrexate pre-filled syringe is **NOT** available as 2.5mg and 5mg pre-filled | | | | |
|  | syringe. | |  |  |  |
|  |  | |  |  |  |
| **Frequency** | Weekly | |  |  |  |
|  |  | |  |  |  |
| **Duration** | 36 weeks | |  |  |  |
|  |  | |  |  |  |
| **Route** | Oral or subcutaneous | |  |  |  |
|  |  | | | | |
| **Formulation** | Decision about formulation used to be made by local clinician, taking into | | | | |
|  | account patient’s preference | | |  |  |
| **Note** | The methotrexate dosing regimen reflects current clinical practice across | | | | |
|  | European paediatric dermatology departments (based on TREAT survey | | | | |
|  | among >300 paediatric dermatologists from 8 European countries) (6). It is also | | | | |
|  | in keeping with the British National Formulary guidance for the treatment of | | | | |
|  | severe cutaneous inflammatory disease in children (19), and that of the | | | | |
|  | American Academy of Dermatology for severe paediatric eczema (27). | | | | |

It is acknowledged that extra precaution is necessary when prescribing and dispensing methotrexate and the following additional measures as listed below should be implemented for this trial in line with standard clinical care:

- The child and their parents/carers must be carefully advised of the dose and frequency and the reason for taking methotrexate and folic acid at each visit.
- Only the 2.5mg strength of methotrexate tablet will be prescribed and dispensed.
- The prescription and the dispensing label will clearly show the dose and frequency of methotrexate administration.
- The child and their parents/carers will be provided with a methotrexate drug monitoring booklet which will provide additional supporting information on dose, frequency and adverse effects monitoring.

**7.3.3** **Methotrexate - Dose Modifications**

Patients will start on an initial test dose (0.1/mg/kg/week) for MTX (week 0) and then continue on the full treatment dose (0.4mg/kg/week for MTX – maximum dose of


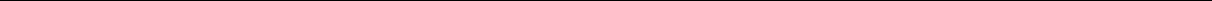


IRAS: 182752 34

TREAT Protocol v7.0 29/07/2020

**EudraCT Number:** 2015-002013-29

25mg/week) from the following week (week 1), providing there have not been any significant side effects and the results from the safety bloods are acceptable (see Table 7).

Please note if the results from the patient’s week 1 safety bloods indicate a degree of toxicity to the initial test dose for MTX but the results are within the trial monitoring parameters (see Table 7), a review by the principal investigator and the chief investigator is required, before a dose adjustment is made.

Patients should remain on the full treatment dose (0.4mg/kg/week for MTX – maximum dose of 25mg/week) until week 12 (visit 5). After that, dose modifications are allowed, according to the treatment response (maximum dose of 25mg/week). For decisions on treatment continuation, PIs should refer to the table below. For situations not included in the below table, this is down to the individual study centre PI and any dose changes will be recorded in the CRF.

**Table 8: Methotrexate dose modification schedule**

***Note:*** *any events that meet the monitoring parameters in the table below should be recorded and reported as an adverse event (see section 10.7)*

| **Monitoring parameters** | **Values** | **Action** |
| --- | --- | --- |
| eGFR | Fall of >=20% (compared with | Repeat renal profile should be |
|  | eGFR value that was used to | done within 2 weeks of the last |
|  | confirm eligibility) | visit. If eGFR falls again >=20 |
|  |  | % following repeat bloods, the |
|  |  | trial nephrologist must be |
|  |  | contacted to discuss potential |
|  |  | dose reduction. If repeat bloods |
|  |  | cannot be obtained within 2 |
|  |  | weeks of the last visit, the trial |
|  |  | nephrologist must be contacted |
|  |  | within 48 hours of the site |
|  |  | becoming aware that a repeat |
|  |  | renal profile cannot be obtained |
|  |  | within the timeframe (2 weeks), |
|  |  | to discuss potential dose |
|  |  | reduction. |
| Blood pressure | >95^th^ centile for age and sex on | MTX dose adjustment, |
|  | two consecutive visits | reduction by at least 20% |
|  |  | initially and patient review. A |
|  |  | temporary dose suspension at |
|  |  | the PI’s discretion is permitted. |
|  |  | Repeat BP after a fortnight. |
| Liver function test | AST, ALT or alkaline phosphatase | MTX dose adjustment |
|  | more than 2× upper limit of | reduction by at least 20% |
|  | reference range | initially or a temporary dose |
|  |  | suspension at the PI’s |
|  |  | discretion. |
|  |  | Repeat LFT weekly until |
|  |  | normal. Further reductions in |
|  |  | dose or stopping medication |
|  |  | may be required but should be |
|  |  | discussed with the Chief |
|  |  | Investigator. |


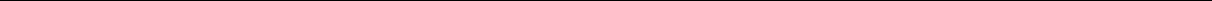


IRAS: 182752 35

TREAT Protocol v7.0 29/07/2020

**EudraCT Number:** 2015-002013-29

| Platelet count | <100x10^9^/L | MTX dose adjustment |
| --- | --- | --- |
|  |  | reduction by at least 20% |
|  |  | initially or a temporary dose |
|  |  | suspension at the PI’s |
|  |  | discretion. |
|  |  | Repeat platelets weekly until |
|  |  | normal. Further reductions in |
|  |  | dose or stopping medication |
|  |  | may be required but should be |
|  |  | discussed with the Chief |
|  |  | Investigator. |
| Neutrophil count | <1.5x10^9^/L | MTX dose adjustment |
|  |  | reduction by at least 20% |
|  |  | initially or a temporary dose |
|  |  | suspension at the PI’s |
|  |  | discretion. |
|  |  | Repeat neutrophils weekly until |
|  |  | normal. Further reductions in |
|  |  | dose or stopping medication |
|  |  | may be required but should be |
|  |  | discussed with the Chief |
|  |  | Investigator. |
| Unexplained bruising, | Not applicable | Review by the PI prior to |
| chicken pox contact or |  | continuing with MTX. |
| rash suspected to be |  |  |
| chicken pox infection |  |  |
| New or worsening | Not applicable | Review by the PI prior to |
| unexplained dyspnoea |  | continuing with MTX |
| or cough |  |  |

The MTX dose/route should remain within the trial parameters (0.4mg/kg/week, maximum dose of 25mg/week/patient) until week 12 (visit 5) on the therapeutic dose. During the first 12 weeks, a dose increase is acceptable for growth (at same dose/kg as at trial entry).

Methotrexate treatment may be suspended for clinical reasons. If for any reason a patient misses a treatment dose, this will be documented in the patient diary but the patient should not discontinue trial treatment or be withdrawn from the trial. Patients who discontinue trial treatment should be asked to continue with study assessments.

**7.4** **Unblinding**

Unblinding is not applicable as PIs, RNs and pharmacists will be aware of treatment allocation. Participants will not be blinded to the treatment allocation either, as there is no placebo and both treatments are given at different frequencies (daily vs weekly).

The blinded assessor who carries out the severity assessment will be blinded to the trial allocation.

**7.5** **Accountability Procedures for Study Treatments**

All IMP is to be sourced via usual NHS procurement arrangements. The research team will liaise with the local pharmacy department to ensure that the site has enough of the IMP in stock with appropriate shelf life to be used in the study.


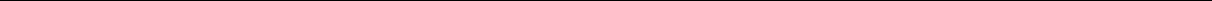


IRAS: 182752 36

TREAT Protocol v7.0 29/07/2020

**EudraCT Number:** 2015-002013-29

Responsible site personnel must maintain accurate accountability records of the IMP dispensed, including details of the manufacturer, name, form, strength, batch number, expiry date and quantity, to whom dispensed and date of transaction. Local procedures should be used if the manufacturer issues a recall.

**7.6 Assessment of Compliance with Study Treatment/s**

Patients should be instructed to return any unused IMPs.

Accountability Log provides space to record IMP supplies returned by trial patients before destruction. Returned or unused IMP doses should be disposed/destroyed on an ongoing basis according to local policy.

**7.7 Concomitant Medications/Treatments**

A concomitant medication/treatment is any drug or substance administered between the screening visit and the visit at week 60. All such medications should be reported to the investigator and recorded on the Concomitant Medications CRF.

**7.7.1** **Medications Permitted**

It is expected that all trial participants will be on concomitant topical therapy for their atopic eczema, in particular regular emollients but also (antiseptic) bath additives and mild-to-potent topical corticosteroids (TCS) of the patient’s/local investigator’s choice. Topical calcineurin inhibitors and oral antihistamines and antibiotics as well as rescue oral corticosteroids are also permitted.

Any medication required for any ongoing illness (illnesses not listed in the exclusion criteria in section 5.2), birth contraception and any rescue medications will also be permitted and recorded both during the treatment and follow up period.

The following are also permitted medications:

Oral contraceptives or hormone-replacement therapy (also refer to section 7.7.2 for potential drug interactions)

Maintenance therapy for other medical conditions listed in the patients’ medical history at the time of screening

Medications for the treatment of asthma which may include but are not limited to inhaled therapies, such as corticosteroids, short acting bronchodilators, combination corticosteroids/long acting bronchodilators, and oral therapies (eg montelukast, theophylline)

Intranasal corticosteroids used for the treatment of allergic rhinitis

Medications such as paracetamol and non-steroidal anti-inflammatory drugs (eg ibuprofen) and routinely-taken dietary supplements, including vitamins, are allowed at the discretion of the local PI and provided that the medication in question has no discernible impact on the study

Fixed regimen of psychiatric medications, including but not limited to tricyclic antidepressants, serotonin reuptake inhibitors, or benzodiazepines

Antiviral medications to treat non-systemic *Herpes simplex* virus (eg acyclovir for cold sores or herpes zoster).

Inactivated vaccine

Antibiotics for treatment of atopic eczema-related skin infections, unless specified in the protocol


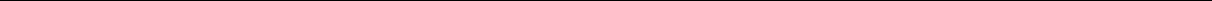


IRAS: 182752 37

TREAT Protocol v7.0 29/07/2020

**EudraCT Number:** 2015-002013-29

Female patients who have attained menarche will undergo a pregnancy test at the screening and baseline visit. They will be counselled against pregnancy during the course of treatment, and will be advised to use highly effective contraceptive measures if appropriate. Any pregnancies that do occur during the study must be reported using a pregnancy CRF to the CTRC within 24 hours of the participating site becoming aware.

**7.7.2** **Medications Not Permitted/ Precautions Required**

The local investigator should instruct the patients and their parents/caregivers to notify the study site about any new medications he/she takes after the start of the study drug.

The following therapies are prohibited during the study **for all patients**:

Use of wet wraps or other occlusive dressings

Concomitant systemic anti-inflammatory or immunosuppressant medication, including but not limited to azathioprine and mycophenolate mofetil

Phototherapy or tanning booth/parlour Allergen immunotherapy

Biologic agents

Live vaccines 4 weeks prior to baseline, while in the study, and for 12 weeks following the last dose of study drug.

Patients who discontinue the study drug due to the use of one of the above should be asked to continue with study assessments.

Patients who use any of the following therapies during the study should not be discontinued from study drug and should not be withdrawn from the study. The investigator must enter the therapies into the concomitant medications CRF. The investigator should contact the trial coordinator (who will contact the Chief Investigator) if questions arise regarding the safety risks associated with continuing study drug.

Other/alternative therapies which may include, but is not limited, to acupuncture, phytotherapy, and herbal substances used for the treatment of atopic eczema. The investigator should ask the patient to stop these therapies for the remainder of the study if in their opinion it is safe to do so.

Patients are advised to avoid self-medication with over the counter ibuprofen if on the methotrexate arm of the study due to the potential interaction between the two medicines. Patients are instead advised to take paracetamol. If accidental use of over the counter ibuprofen occurs, this will not lead to the withdrawal of the patient from the study drug or trial, and clinicians are advised to monitor closely in line with current practice.

**7.7.2.1 Those randomised to ciclosporin**

Ciclosporin is extensively metabolised by CYP 3A isoenzymes, in particular CYP3A4, and is a substrate of the multidrug efflux transporter P-glycoprotein. Various drugs are known to either increase or decrease plasma or whole blood concentrations of ciclosporin.

Treatment with the following concomitant medications is prohibited during the study:

Bosentan

Dabigatran etexilate

Aliskiren


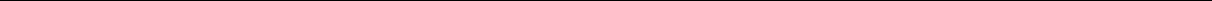


IRAS: 182752 38

TREAT Protocol v7.0 29/07/2020

**EudraCT Number:** 2015-002013-29

Hypericum perforatum (St. John's wort)

Tacrolimus (except for topical treatment – see section 7.7.1)

Everolimus and sirolimus

Lercanidipine

Treatment with a live (attenuated) vaccine

Other drugs known to interact with ciclosporin may be prescribed at the discretion of the local Investigator when considered necessary for the patient's safety and well-being. If drugs are given concomitantly, careful monitoring for drug-related adverse effects is recommended in line with clinical practice. Investigators should refer to the SmPC for further information, and the following points should be considered:

Drugs that decrease ciclosporin concentration include:

Barbiturates (e.g. Phenobarbital), carbamazepine, oxcarbazepine, phenytoin; nafcillin, intravenous sulfadimidine, probucol, orlistat, ticlopidine, sulfinpyrazone, terbinafine, rifampicin, octerotide.

Drugs that may increase ciclosporin concentration include:

Nicardipine, metoclopramide, oral contraceptives, methylprednisolone (high dose), allopurinol, cholic acid and derivatives, protease inhibitors, imatinib, colchicine, nefazodone.

Macrolide antibiotics: azithromycin, clarithromycin,

Erythromycin (Note: Erythromycin may be used as rescue medicine but it should not be used for longer than two weeks in the trial).

Azole antibiotics: Ketoconazole, fluconazole, itraconazole and voriconazole

Verapamil

Telaprevir

Amiodarone

Danazol

Diliazem

Imatinib

Care should also be taken when using ciclosporin together with other active substances that exhibit nephrotoxic synergy such as: aminoglycosides (including gentamycin, tobramycin), amphotericin B, ciprofloxacin, vancomycin, trimethoprim (+ sulfamethoxazole); fibric acid derivatives (e.g. bezafibrate, fenofibrate); NSAIDs (including diclofenac, naproxen, sulindac); melphalan histamine H2-receptor antagonists (e.g. cimetidine, ranitidine); methotrexate.

Ciclosporin is also an inhibitor of CYP3A4, the multidrug efflux transporter P-glycoprotein and organic anion transporter proteins (OATP) and may increase plasma levels of co-medications that are substrates of this enzyme and/or transporters. Ciclosporin may reduce the clearance of digoxin, *colchicine, statins, etoposide* prednisolone, non-steroidal anti-inflammatory drugs (NSAIDs), and others.

**7.7.2.2 Those randomised to methotrexate**

Drugs known to interact with methotrexate may be prescribed at the discretion of the local Investigator when considered necessary for the patient's safety and well-being. If drugs are given concomitantly, careful monitoring for drug-related adverse effects is recommended in line with clinical practice. Investigators should refer to the SmPC for further information, and the following points should be considered:

Concomitant use of hepatotoxic medicinal products

Concomitant use of haematotoxic medicinal products


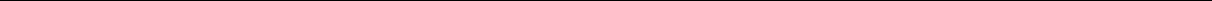


IRAS: 182752 39

TREAT Protocol v7.0 29/07/2020

**EudraCT Number:** 2015-002013-29

Oral antibiotics like tetracyclines, chloramphenicol, and non-absorbable broad-spectrum antibiotics can interfere with the enterohepatic circulation, by inhibition of the intestinal flora or suppression of the bacterial metabolism

Antibiotics, like penicillines, glycopeptides, sulfonamides, ciprofloxacin and cefalotin can, in individual cases, reduce the renal clearance of methotrexate

Methotrexate is plasma protein bound and may be displaced by other protein bound drugs such as salicylates, hypoglycaemics, diuretics, sulphonamides, diphenylhydantoins, tetracyclines, chloramphenicol and p-aminobenzoic acid, and the acidic anti-inflammatory agents, which can lead to increased toxicity when used concurrently.

Probenecid, weak organic acids such as loop diuretics, and pyrazoles (phenylbutazone) can reduce the elimination of methotrexate

The concomitant administration of products which cause folate deficiency (e.g. sulphonamides, trimethoprim-sulphamethoxazole) can lead to increased methotrexate toxicity.

Vitamin preparations or other products containing folic acid, folinic acid or their derivatives may decrease the effectiveness of methotrexate.

Concomitant use of mercaptopurine

Concomitant administration of proton-pump inhibitors like omeprazole or pantoprazole may delay clearance of methotrexate.

Methotrexate may decrease the clearance of theophylline

Combined treatment with methotrexate and retinoids like acitretin or etretinate increases the risk of hepatotoxicity

Levetiracetam

For any other medication in question, the local investigator should consult the prescribing information (Summary of product characteristics: [www.medicines.org.uk/emc/](http://www.medicines.org.uk/)).The trial coordinator should be contacted (who will contact the study CI) to ensure that there are no safety risks associated with continuing study drug.

**7.7.3 Data on Concomitant Medication**

All concomitant medication must be recorded on the subject’s CRF, according to instructions for CRF completion.

**7.8 Co-enrolment Guidelines**

To avoid potentially confounding issues, ideally patients should not be recruited into other trials. Individuals who have participated in a trial testing a medicinal product within 28 days or less than 5 half lives of the IMP preceding screening will be ineligible for the TREAT trial. Where recruitment into another trial is considered to be appropriate and without having any detrimental effect on the TREAT trial, this must first be discussed with the CTRC who will contact the Chief Investigator Professor Carsten Flohr.


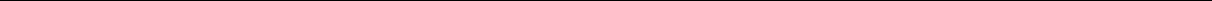


IRAS: 182752 40

TREAT Protocol v7.0 29/07/2020

**EudraCT Number:** 2015-002013-29

**8 ASSESSMENTS AND PROCEDURES**

**8.1 Schedule for assessments during the treatment and the follow-up phase**

The total duration of the study is for 60 weeks (following randomisation). Patients will receive IMP for 36 weeks and followed up for 60 weeks post randomisation. The below interventions will take place according to the study visit schedule table at the stated time points. However, the following visit windows are allowed: 2 days +/- at week 1 and 2, 1 week +/- at week 4, and then 2 weeks +/- thereafter.


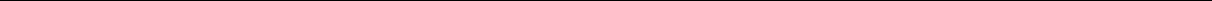


IRAS: 182752 41

TREAT Protocol v7.0 29/07/2020

**EudraCT Number:** 2015-002013-29

**Table 9: Schedule of study procedures**

**
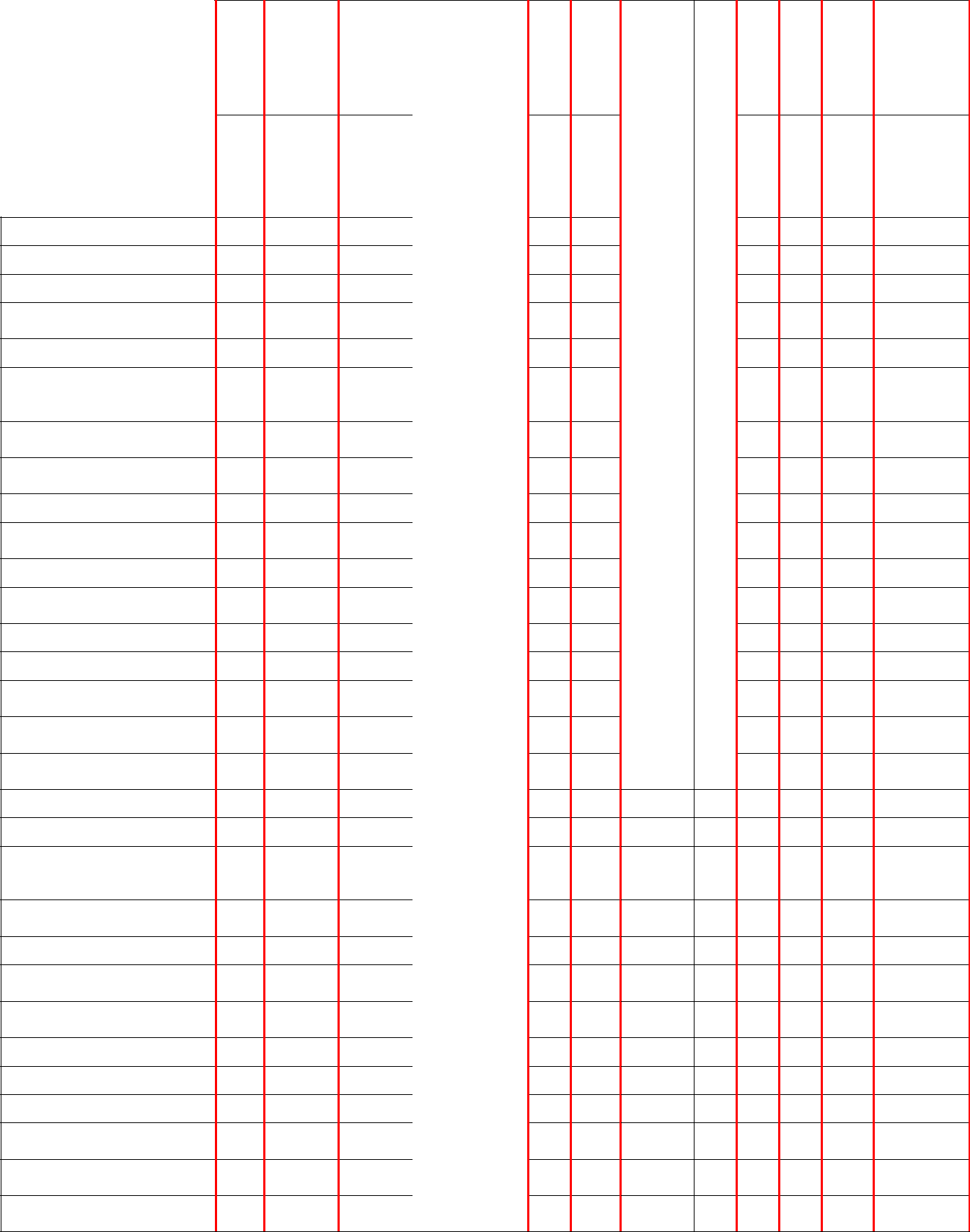
**

**Procedures**

Informed consent

Inclusion/exclusion criteria

Medical history

Concomitant drugs Demographics

Physical exam (including mouth/throat examination and chest auscultation)

o-SCORAD)

EASI, IGA

POEM (patient assessed)

Parent and child QoL (CDLQI/IDQOL & DFI)

Child HRQL (CHU-9D)

Resource Use (patient diary)

Height & weight Height

Blood pressure

Adverse events (AE & SAE)

**a**

Safety bloods **^b^**

Chest X-Ray **^d^**

Pregnancy test (beta-HCG)

Confirmation of appropriate contraception use, where applicable

Urine sample collection (NAG)

Randomisation

Study drug dispensing (as needed at each visit)

MTX metabolite level (blood)

CyA trough level (blood) **^e^**

Cystatin C level (blood)

Creatinine level (blood)

Tape stripping for cutaneous metabolic work **^f^**

Collection of blood for mechanistic studies **^g^**

Collection of blood/saliva for FLG genotyping ^i^

| **Visit Screening** |
| --- |

**x**

**x**

**x**

**x**

**x**

**x**

**x**

1. **^c^**

**x**

**x**

**x**

| **Week 0** |
| --- |

| **ation Randomis Baseline/** |
| --- |

**x**

**x**

**x**

**x**

**x**

**x**

**x**

**x**

**x**

**x**

**x**

**x**

**x**

**x**

**x**

**x**

**x**

**x**

**x**

**x**

**x**

| **arm** |
| --- |
| **1** |

| **Visit 1 only) (MTX Week** |
| --- |

**x**

**x**

**x**

**x**

**x**

**x**

| **Week 2** | **Week 4** |
| --- | --- |
|  |  |
| **Visit 2** | **Visit 3** |
|  |  |
|  |  |
|  |  |
|  |  |
| **x** | **x** |
|  |  |
| **x** | **x** |
|  | **x** |
|  | **x** |
|  | **x** |
|  |  |
|  |  |
|  | **x** |
| **x** | **x** |
|  |  |
| **x** | **x** |
| **x** | **x** |
| **x** | **x ^c^** |
|  |  |
|  |  |
| **x** | **x** |
| **x** |  |
|  |  |
|  | **x** |
| **x** |  |
| **x** |  |
| **x** |  |
| **x** |  |
|  |  |
|  |  |
|  |  |


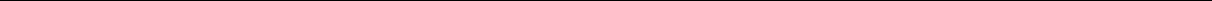


| **Visit 4 Week 8** |
| --- |

**x**

**x**

**x**

**x**

**x**

**x**

**x**

**x**

**x**

**x**

**x**

**x**

**x**

**x**

**x**

**x**

| **Visit 5 Week 12** |
| --- |

**x**

**x**

**x**

**x**

**x**

**x**

**x**

**x**

**x**

**x**

**x**

**x**

**x**

**x**

**x**

**x**

**x**

**x**

**x**

**x**

**x**

| **Week 20** | **Week 28** |
| --- | --- |
|  |  |
| **Visit 6** | **Visit 7** |
|  |  |
|  |  |
|  |  |
|  |  |
| **x** | **x** |
|  |  |
| **x** | **x** |
| **x** | **x** |
| **x** | **x** |
| **x** | **x** |
|  |  |
|  |  |
| **x** | **x** |
| **x** | **x** |
|  |  |
| **x** | **x** |
| **x** | **x** |
| **x** | **x** |

1. **x**
2. **x**

| **Visit 8 Week 36** |
| --- |

**x**

**x**

**x**

**x**

**x**

**x**

**x**

**x**

**x**

**x**

**x**

**x**

**x**

**x**

**x**

**x**

**x**

**x**

**x**

**x**

| **Visit 9 Week 48** |
| --- |

**x**

**x**

**x**

**x**

**x**

**x**

**x**

**x**

**x**

| **Visit 10 Week 60** |
| --- |

**x**

**x**

**x**

**x**

**x**

**x**

**x**

**x**

**x**

**x**

**x**

**x**

**x**

**x**

**x**

**x**

| **d visit Unschedule** |
| --- |

**x**

1. **^h^**
2. **^h^**

**x (only if required)**

**x**

**x (only if required)**

**x**

IRAS: 182752 42

TREAT Protocol v7.0 29/07/2020

**EudraCT Number:** 2015-002013-29

1. Collect until **4 weeks** after treatment stopped
2. Safety bloods includes assessment of liver function, renal function and full blood count **^c^ Lipids to also be assessed at these time points as part of safety bloods**
3. Screening chest X-Ray on discretion of the local PI/medically qualified physician in those at risk of TB
4. Collection of blood for ciclosporin levels should be measured in the morning, 12 hours (+/-30 minutes) after the previous evening’s dose, immediately prior to the administration of the morning dose. In younger children, where regular ciclosporin dosing occurs prior to school and in the early evening prior to bedtime (e.g. 0730 and 1930), on the evenings prior to study visits where the ciclosporin level is to be measured, the evening dose should be given later in accordance with the time of the visit appointment.
5. Not collected by all participating sites
6. Samples only collected from patients recruited at St Thomas’ Hospital
7. Severity assessments only to be collected if an unscheduled visit occurs between week 36 and week 60

^I^ If blood/saliva for FLG genotyping not collected at randomisation visit, the sample can be collected at any visit post randomisation.

**8.1.1** **Assessments:**

1. Children will be assessed by trained blinded assessors at 0, 4, 8, 12, 20, 28, 36, 48 and 60 weeks (o-SCORAD, EASI, & IGA). POEM (included in the patient diary) will be completed by the patient/parent at home.
2. The number of flares, side effects and adverse events during treatment will be captured through questionnaires administered by the research nurses and through a diary collected by the patient or parent/guardian.
3. Child and parental QoL (CDLDI/IDQOL/DFI) & CHU-9D will be assessed at baseline, 12, 36, 48 and 60 weeks.
4. Blood for drug metabolite/trough levels will be taken at 2, 8, 12, 36 weeks.
5. Blood and tape strips for mechanistic work will be taken at baseline, 12, 36, and 60 weeks. Mechanistic blood samples and tape strip samples will only be collected from patients recruited at St Thomas’ Hospital.
6. A blood sample will be collected at the week 0 visit for filaggrin analysis. If a blood sample cannot be collected a saliva sample will be used for this analysis.
7. AE and SAE data will be collected, until 4 weeks after treatment has stopped.

**8.1.2 Visit Summary**

The following provides the activities to be completed at each visit:

**Screening visit**

Informed consent

Inclusion/exclusion criteria Medical history

Concomitant drugs Demographics

o-SCORAD Height

Safety bloods*

Pregnancy test for females of child bearing potential

Chest X-Ray in those at risk of TB (discretion of local PI/medically qualified physician) *Lipid assessment will occur as part of safety bloods at this time point.


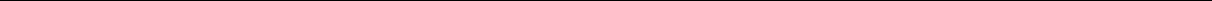


IRAS: 182752 43

TREAT Protocol v7.0 29/07/2020

**EudraCT Number:** 2015-002013-29

**Baseline/randomisation visit – Week 0**

Inclusion/exclusion criteria Concomitant drugs

Pregnancy test for females of child bearing potential (see section 6.3) Physical exam

o-SCORAD EASI, IGA

POEM (patient assessed) Parent & child QoL

Height & weight Blood pressure

Urine sample collection (NAG) Randomisation

Study drug dispensing

Blood sample for cystatin C level and creatinine level

Tape stripping for cutaneous metabolic work (only at St Thomas’ Hospital) Collection of blood for mechanistic studies (only at St Thomas’ Hospital)

Collection of blood/saliva for FLG genotyping (if sample is not collected at the randomisation visit, the sample can be collected at any visit post randomisation)

**Visit 1 – Week 1 (only for methotrexate arm)**

Safety bloods

Height & weight

Adverse events

Concomitant medications

Physical examination

**Visit 2 – Week 2**

Concomitant drugs Height & weight

Physical exam Blood pressure

Adverse events (AE & SAE) Safety bloods

Urine sample collection (NAG)


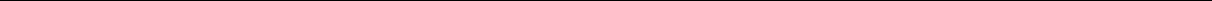


IRAS: 182752 44

TREAT Protocol v7.0 29/07/2020

**EudraCT Number:** 2015-002013-29

Collection of blood for MTX/CyA metabolite/trough levels, cystatin C level and creatinine level

**Visit 3 – Week 4**

Concomitant drugs Height & weight

Physical exam o-SCORAD

EASI, IGA

POEM (patient assessed) Blood pressure

Adverse events (AE & SAE) Safety bloods*

Study drug dispensing

*Lipid assessment will occur as part of safety bloods at this time point.

**Visit 4 – Week 8**

Concomitant drugs Height & weight

Physical exam o-SCORAD

EASI, IGA

POEM (patient assessed) Blood pressure

Adverse events (AE & SAE) Safety bloods

Study drug dispensing

Collection of blood for MTX/CyA metabolite/trough levels, cystatin C level and creatinine level

**Visit 5 – Week 12**

Concomitant drugs Physical exam

o-SCORAD EASI, IGA

POEM (patient assessed)


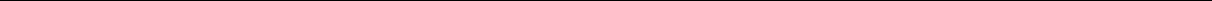


IRAS: 182752 45

TREAT Protocol v7.0 29/07/2020

**EudraCT Number:** 2015-002013-29

Parent & child QoL Height & weight

Blood pressure

Adverse events (AE & SAE) Safety bloods

Urine sample collection (NAG) Study drug dispensing

Collection of blood for MTX/CyA metabolite/trough levels, cystatin C level and creatinine level

Tape stripping for cutaneous metabolic work Collection of blood for mechanistic studies

**Visit 6 – Week 20**

Concomitant drugs Height & weight

Physical exam o-SCORAD

EASI, IGA

POEM (patient assessed) Blood pressure

Adverse events (AE & SAE) Safety bloods

Study drug dispensing

**Visit 7 – Week 28**

Concomitant drugs Height & weight

Physical exam o-SCORAD

EASI, IGA

POEM (patient assessed) Blood pressure

Adverse events (AE & SAE) Safety bloods

Study drug dispensing

**Visit 8 – Week 36**

**
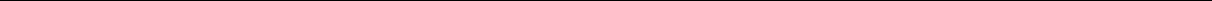
**

IRAS: 182752 46

TREAT Protocol v7.0 29/07/2020

**EudraCT Number:** 2015-002013-29

Concomitant drugs Physical exam

o-SCORAD EASI, IGA

POEM (patient assessed) Parent & child QoL

Height & weight Blood pressure

Adverse events (AE & SAE) Safety bloods

Urine sample collection (NAG)

Collection of blood for MTX/CyA metabolite/trough levels, Cystatin C level and Creatinine level

Tape stripping for cutaneous metabolic work Collection of blood for mechanistic studies

**Visit 9 – Week 48**

Concomitant drugs Physical exam

o-SCORAD EASI, IGA

POEM (patient assessed) Parent & child QoL

**Visit 10 – Week 60**

Concomitant drugs Physical exam

o-SCORAD EASI, IGA

POEM (patient assessed) Parent & child QoL

Height & weight Blood pressure Safety bloods

Urine sample collection (NAG)

Collection of blood for Cystatin C level and Creatinine level Tape stripping for cutaneous metabolic work


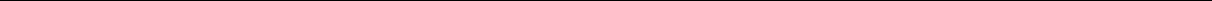


IRAS: 182752 47

TREAT Protocol v7.0 29/07/2020

**EudraCT Number:** 2015-002013-29

Collection of blood for mechanistic studies

**Unscheduled Visits**

In some circumstances a patient may need to return to the site for a non-routine visit. The following data should be recorded on the follow up CRF:

Source of unscheduled visit Reason for return

Related adverse events

Safety bloods (only collected if part of the reason for the unscheduled visit) Concomitant medications

Blood pressure (only if part of the reason for the unscheduled visit) Outcome of the visit

Severity assessment (o-SCORAD, EASI & IGA) data only collected if the unscheduled visit occurs between week 36 and week 60

**Patient Diary (including POEM)**

All patients and/or parent/legal guardian will be asked to complete a diary during treatment and a separate diary during follow up. The diary will ask participants to record how often they have taken the study medication and if they have experienced any side effects. The diary will also include POEM, questions on flares and use of medications for eczema. On a monthly basis, there will be questions to find out if it has been necessary to see a health care professional, whether antibiotic creams or tablets were prescribed for a skin infection and whether any time off nursery or school was required. Patients will be given more than one patient diary to complete at home on occasions where the study visits are longer than 4 weeks apart.

**8.2 Procedures for Assessing Efficacy**

Efficacy of trial treatments will be assessed throughout the period of the study using objective measures.

The first co-primary outcome measure for the trial is change in atopic eczema severity between baseline and 12 weeks of treatment, using the objective SCORing Atopic Dermatitis (o-SCORAD) severity index, conducted by the blinded assessor (independent member of the research team) at baseline and at 12 weeks.

The second co-primary outcome is the time to the first significant flare after treatment cessation in the MTX vs CyA groups. The total number of flares will also be recorded in questionnaires. The time to the first significant flare is defined as the (earliest) time that a patient either re-started systemic treatment or returns to their baseline o-SCORAD (or worse), following cessation of trial treatment^*^.

The secondary outcome of change in atopic eczema severity using the o-SCORAD, EASI, IGA and Patient Oriented Eczema Measure (POEM) scores will be assessed between baseline and 12, 36, 48, 60 weeks.


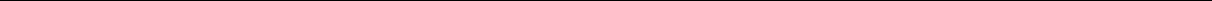


IRAS: 182752 48

TREAT Protocol v7.0 29/07/2020

**EudraCT Number:** 2015-002013-29

- This refinement to the definition of the co-primary outcome was made, as it was felt that many patients may not ever return to their baseline (or worse) o-SCORAD following treatment cessation but still require systemic therapy because of the severity of their disease and/or the impact this has on their quality of life. The original wording of the definition did not fully reflect real world practice and treatment of a significant disease re-flare after systemic treatment cessation. The TMG consequently proposed the suggested refinement of the definition which was subsequently agreed by the independent oversight committees of the trial, the IDSMC and TSC. Those involved in the decision making were blind to all study results.

**8.3 Procedures for Assessing Safety**

The following safety bloods and investigations will be performed:

1. Safety blood profiles (full blood count, renal function and liver function) will be taken at screening, 2, 4, 8, 12, 20, 28, 36 and 60 weeks. Lipids will also be assessed at screening and week 4 as part of the safety blood profile. Safety bloods will also be taken at week 1 for the methotrexate arm. If it is not possible to obtain a safety blood sample (e.g. insufficient sample, participant refusal) at their visit, the remaining trial procedures can be carried out as normal and the patient can continue in the trial. However, the participant should be advised to temporarily stop trial treatment, until further advice is sought. The CTU should be contacted with the following information:

Details of which safety blood values are missing Date started treatment

Date and reason of any dose changes previously required

Details of any previous blood samples that had safety implications Date of next scheduled visit

CTU will then liaise with CI (or delegate) and sponsor and will advise the site regarding the necessity to arrange an unscheduled visit to obtain missing safety assessment/s and when to reintroduce trial treatment. If a participant is not able to comply with the requested measures then permanent discontinuation of trial treatment may be considered, though in these circumstance the participant will be asked to continue in the study for follow-up purposes.

Please note, the above process does not apply to safety bloods taken at week 1 for the methotrexate arm, as this sample must be taken.

1. BP will be taken at baseline, 2, 4, 8, 12, 20, 28, 36 and 60 weeks
2. Chest X-Ray in those at risk of TB (discretion of local PI/medically qualified physician) at screening visit
3. Physical examination will be carried out at each visit (except at the screening visit).

An Independent Data and Safety Monitoring Committee (IDSMC) will be constituted and will monitor adverse events and patient safety during the trial. Data on adverse events, including severity, seriousness, and expectedness as part of pharmacovigilance will be recorded at each follow-up visit and communicated to the CTRC. Requirements for pharmacovigilance reporting is detailed fully in Section 10 (Pharmacovigilance).

**8.4 Other Assessments**

**8.4.1** **Quality of Life**

Quality of life assessments of the patient and family will be conducted via paper-based questionnaires and quality of life sheets to the patients and carers. If necessary the RN/member of the research team will provide guidance on how to complete the


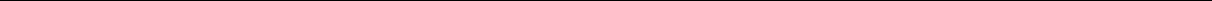


IRAS: 182752 49

TREAT Protocol v7.0 29/07/2020

**EudraCT Number:** 2015-002013-29

questionnaires and will collect them from the patients and carers at the end of the study visits.

The following quality of life questionnaires will be administered to the patient and/or parent/guardian:

**CDLQI**

To be completed by the patient (4 years – 16 years) during the visit. There is no difference between the questions of either the CDLQI – older children and the CDLQI – with cartoons. The researcher should assess which CDLQI questionnaire will be most appropriate for each individual child.

**IDQOL**

To be completed by the parent/guardian for patients who are below the age of 4 years during the visit.

**DFI**

To be completed by the parent/guardian during the visit.

**CHU-9D**

Proxy Child Health Utility 9D is to be completed by parent/guardian for patients who are less than 7 years of age at time of recruitment.

Self-complete Child Utility 9D is to be completed by patients who are aged 7 years and over at time of recruitment.

Please note that if a patient turns 7 years old whilst on the trial, the participating site must continue to administer the proxy CHU-9D for consistency.

**8.4.2** **Health Economics**

Information relating to the health economic evaluation, such as GP visits and attendance at other healthcare professionals due to their eczema, will also be gathered via a paper based participant completed diary. The diary will also record whether medications were required to treat a skin infection and whether the participant has required time off school or nursery due to their eczema.

Upon review of the participant diary, the research nurses will contact participants/parent/ guardian via phone in order to collect any missing data.

A within-trial cost-effectiveness analysis will be conducted to assess whether CyA offers value for money compared to MTX for children with moderate-to-severe AE from the perspective of the NHS and personal social services (28). The focus of the economic evaluation will be on estimating cost and effect differences and quantifying the likelihood that the intervention is cost-effective.

Intervention resource use (including drug use and safety monitoring) will be recorded by the trial CTU. *Wider resource use (including GP and practice nurse consultations, hospital admissions and prescriptions for eczema treatments) will be elicited via study diaries at baseline and weeks 4, 8, 12, 20, 28, 36, 48 and 60. Published unit costs will be attached to items for a common price year to determine the mean overall cost in each arm. Costs and


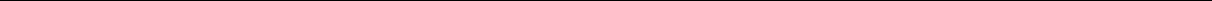


IRAS: 182752 50

TREAT Protocol v7.0 29/07/2020

**EudraCT Number:** 2015-002013-29

outcomes will be discounted in weeks 53 to 60 to reflect the timeframe being greater than 12 months.

Incremental cost-effectiveness will be assessed by estimating the cost per unit change in o-SCORAD and POEM. These can be compared with estimates from other interventions which have been evaluated using these outcomes.

However, there is no straightforward means of assessing that cost/unit change in effectiveness represents a good use of NHS resources. A cost-utility study which provided a cost/QALY gained would be more interpretable across a broader range of medical conditions. Therefore, Quality-Adjusted Life Years for the trial period (based upon CHU-9D captured at baseline and weeks 12, 36, 48 and 60 using the proxy version for those aged under 7 years at time of recruitment) will be estimated using linear interpolation and are under the curve with baseline adjustment (29).

Using this information on costs and benefits, an incremental cost utility analysis will be conducted using accepted methodology (30-32).

- GPs will be approached to complete a short questionnaire to capture wider resource use recorded in the participant’s medical records. The information requested will cover the period the participant has been in the trial and 4 weeks prior to their randomisation visit. GPs will be compensated for completed returned questionnaires. Participating sites will post out the questionnaire and a cover letter to the participants’ GPs. However, if participating sites have direct access to the participant’s GP records via a shared electronic database, the research team may complete the questionnaire. All participants and/or parent/guardian will be provided with a full explanation before obtaining informed verbal consent (whether provided by participant’s GP or participating site).

**8.4.3** **Special Assays or Procedures**

**Blood sample collection**

The following blood samples will be collected for mechanistic work at weeks 0 (baseline), 12,

36 and 60 using standard phlebotomy techniques:

a). 1x 5 ml Z serumSep Clot Activator vacuette tube (gold top) for blood serum

b). 1x remaining blood allowed for immunology (fresh PBMCs) and metabolic work (frozen PBMCs)

**Sample transport, processing and storage for mechanistic work:**

We aim to collect mechanistic samples for all patients recruited at St Thomas’ Hospital at each of the four time points (0, 12, 36, and 60 weeks) for the immunology and metabolic work, and we will make use of our own fully established biobanking facilities at King’s College London (KCL) to process and store samples.

Samples will be transported by courier, ensuring that they are fully processed by 4pm on the same day and within a maximum of 6 hours post venesection. The lab work will commence at the end of the trial and will be performed by full-time lab technicians in London.

**Cutaneous tape strip collection:**

Round adhesive tape discs (3.8 cm2, D-Squame; CuDerm, Dallas, Tex) will be attached to the skin of the forearm. Each tape will be pressed onto the volar aspect of the forearm for 10 seconds with standardized force by using a disc pressure applicator (CuDerm). The tape strip will be gently removed with tweezers and stored in a closed vial at -80^o^C until analysis. The first strip will be discarded because it might contain dirt and remnants of cosmetic


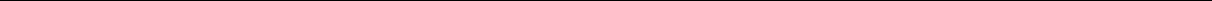


IRAS: 182752 51

TREAT Protocol v7.0 29/07/2020

**EudraCT Number:** 2015-002013-29

products; the second to sixth tape strips will be applied on the same skin spot. The tape strip samples will only be collected from patients recruited from St Thomas’ Hospital.

**Mechanistic studies:**

*Immunological parameters:*

We will study whether significant changes are observed in the percentages of Tregs, in the percentages of pro/anti-inflammatory cytokine-expressing CD4+ T cells, or in the corresponding levels of these cytokines in serum following treatment, whether this is different in MTX vs CyA treated patients, and whether this correlates to response to treatment at 12 and 36 weeks and the risk of re-flares at 60 weeks.

*Systemic and local metabolic parameters:*

We will examine whether the initial treatment response at 12 weeks to MTX (vs CyA) is already associated with differences in the systemic metabolic profiles (shift from pro-inflammatory glycolytic activation to an anti-inflammatory metabolic profile), and whether this is also seen at 36 and 60 weeks, explaining a more sustained disease remission following MTX (vs CyA) therapy. We will also study whether the observed systemic metabolic changes are associated with corresponding metabolic profiles and gene expression in the skin.

**Urine (NAG)**

Urine samples will be collected for mechanistic work at weeks 0, 2, 12. 36 and 60.

**Sample transport, processing and storage**

The following samples will require processing and storage at participating sites prior to transporting to the KCL Bio Bank/external laboratories (pg. 4-6): -Cystatin C

-Creatinine

-MTX metabolite levels

-Ciclosporin trough levels

-Urine (NAG)

-Tape stripping

-FLG

Processing of the samples can be carried out by a local research nurse or laboratory technician. Sample collection, processing and transport requirements are detailed in a sample manual, which will be provided to participating sites, and training in this will be provided at the site training visit.

**8.5 Loss to Follow-up**

If any of the trial participants are lost to follow up contact will initially be attempted through the PI or designated research staff at each centre. If the lead investigator at the trial centre is not the participant’s usual clinician responsible for their specialist care then follow up will also be attempted through this latter clinician.

Where possible, information on the reason for loss to follow up will be recorded.

**8.6 Trial Closure**

The end of the trial is defined to be the date on which data for all participants is frozen and data entry privileges are withdrawn from the trial database. However, the trial may be closed prematurely by the Trial Steering Committee (TSC), on the recommendation of the


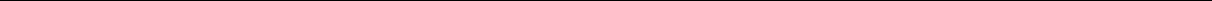


IRAS: 182752 52

TREAT Protocol v7.0 29/07/2020

**EudraCT Number:** 2015-002013-29

Independent Data and Safety Monitoring Committee (IDSMC). Should the trial be closed prematurely, all active participants (receiving treatment or in follow up) will be called in for a final follow up visit and assessments will be undertaken. Ongoing care will be at the discretion of the treating clinician.


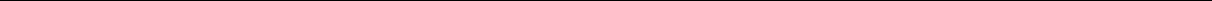


IRAS: 182752 53

TREAT Protocol v7.0 29/07/2020

**EudraCT Number:** 2015-002013-29

**9 STATISTICAL CONSIDERATIONS**

**9.1 Introduction**

A separate and full statistical analysis plan (SAP) will be developed prior to the final analysis of the trial. The main features of these planned statistical analyses are included here in the main protocol.

**9.2 Method of Randomisation**

Participants will be randomised using a secure (24-hour) web-based randomisation programme controlled centrally by the CTRC. Randomisation lists will be generated in a 1:1 ratio using simple block randomisation with random variable block length (see section 6.4 for back-up randomisation method).

**9.3** **Outcome Measures**

**9.4 Co-Primary Endpoints**

1. Change in atopic eczema severity between baseline and 12 weeks of treatment, using the o-SCORAD index.
2. Time to first significant flare after treatment cessation in the MTX vs CyA groups.

**9.5** **Secondary Endpoint(s)**

1. Change in atopic eczema severity using the EASI, IGA, o-SCORAD and POEM between baseline and 12, 36, 48, and 60 weeks.
2. Number of flares in each study arm as well as the proportion of children who re-flared following treatment cessation.
3. Proportion of participants achieving 50% improvement in the o-SCORAD index at 12, 36, 48, and 60 weeks.
4. Proportion of participants who withdraw from treatment because of AEs.
5. Disease-specific patient and parental quality of life (QoL) measured with the CDLQI/IDQOL and DFI scores between baseline and 12, 36, 48 & 60 weeks.
6. Assess the cost-effectiveness of CyA vs MTX, based on utility measured using the CHU-9D.
7. Immuno-metabolic effects of MTX and CyA, especially in relation to markers of glycolytic activation and T cell cytokine signature, at baseline, during treatment and post treatment cessation.
8. Drug-related side effects of both MTX and CyA and their association with MTX polyglutamate and CyA trough levels.
9. The association between MTX polyglutamate and CyA trough levels and treatment response (reduction in disease severity).


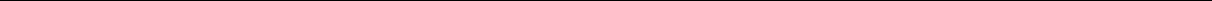


IRAS: 182752 54

TREAT Protocol v7.0 29/07/2020

**EudraCT Number:** 2015-002013-29

10. The association between FLG carriage (yes/no) and treatment response.

**9.6 Sample Size**

For the first primary outcome (o-SCORAD), the change from baseline to 12 weeks will be calculated for each participant. The study aims to detect a difference of 8 o-SCORAD points, assuming a standard deviation (SD) of 10 (based on the only other paediatric RCT with systemic immunosuppressive medication in children (7), which saw a SD of 6.3 (MTX arm) vs 8.9 (CyA arm) at 12 weeks) a sample size of 41 per group, increasing to 49 per group to allow for an estimated 18% loss to follow up, will be required to provide 90% power using a t-test with a 0.025 two-sided significance level. Our assumption of 8 Units in the o-SCORAD index representing the minimal clinically important difference between treatments is based on calculations from three other RCTs with immuno-suppressive medication in children and adults with severe atopic eczema (33).

The co-primary outcome of this trial is time to first significant flare after treatment cessation (return to baseline o-SCORAD (or worse) or re-starting systemic treatment), as this may be an important factor influencing potential change in prescribing behaviour. The number of patients on CyA burst treatment who went into remission after the first three months of treatment in the study by Harper et al was three out of 21, indicating that 86% of patients re-flared. Assuming a similar flare risk in our CyA group, a sample size of 43 in each group (51 in each group with estimated loss to follow up of 18%) will have 80% power to detect a reduction in re-flare of 30% (from 86% to 56%) using a two-sided test with a 0.025 significance level.

Randomising a total of 102 participants, 51 into each of the study arms, satisfies both sample size calculations.

**9.7** **Interim Monitoring and Analyses**

Formal interim analyses of the accumulating data will be performed at regular intervals (at least annually) for review by an Independent Data Monitoring and Safety Committee (IDSMC). These analyses will be performed at the Clinical Trials Research Centre. The IDSMC will be asked to give advice on whether the accumulated data from the trial, together with results from other relevant trials, justifies continuing recruitment of further patients or further follow-up. A decision to discontinue recruitment, in all patients or in selected subgroups will be made only if the result is likely to convince a broad range of clinicians including participants in the trial and the general clinical community. If a decision is made to continue, the IDSMC will advise on the frequency of future reviews of the data on the basis of accrual and event rates. The IDSMC will make recommendations to the Trial Steering Committee (TSC, see section 16) as to the continuation of the trial.

After the primary outcome data are available from 25 patients (o-SCORAD index at 12 weeks) the standard deviation of the 25 scores, and the 95% confidence limits for this estimate, will be calculated without unblinding allocation. If the 95% confidence limits of the estimate of the SD of the o-SCORAD index at 12 weeks overlap 10 the trial will continue unchanged.If the upper 95% confidence limit of the estimate of the SD of the o-SCORAD index at 12 weeks is less than 10 the trial will continue unchanged but the TSC will be informed that the trial power is greater than planned. If the lower 95% confidence limit of the estimate of the SD is greater than 10 the study is underpowered. EME will decide whether to invite an extension or close the study.

A comprehensive statistical analysis plan will be developed before any formal statistical analyses are carried out.


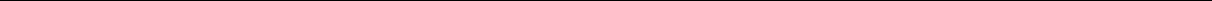


IRAS: 182752 55

TREAT Protocol v7.0 29/07/2020

**EudraCT Number:** 2015-002013-29

The primary analysis will use the principle of intention to treat based on all the randomised participants, as far as is practically possible. If consent for treatment is withdrawn but the participant is happy to remain in the study for follow-up, they will be followed up until completion. However if they decide to withdraw consent completely then the reasons for withdrawal of consent will be collected (if possible) and reported for both groups.

The analysis of change in o-SCORAD from baseline to 12 weeks will use the method of analysis of covariance and the covariates that will be used in the model will be treatment group and the baseline measurement

Analysis of time to first significant flare after treatment cessation will be summarised by Kaplan-Meier curves for each treatment group and compared overall using the log rank test and survival regression methods. The time to first significant flare after treatment cessation will be measured from randomisation.

For the secondary outcomes, continuous data will be reported as difference in means and will be analysed using ANCOVA where appropriate and binary data will be reported in terms of relative risk with appropriate 95% confidence intervals.

Missing data will be monitored and strategies developed to minimise its occurrence. Missing data will be handled by considering the robustness of the complete case analysis to sensitivity analyses using various imputation assumptions; however these will be informed by data collected on the reasons for missing data.


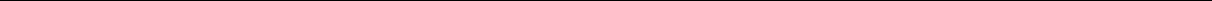


IRAS: 182752 56

TREAT Protocol v7.0 29/07/2020

**EudraCT Number:** 2015-002013-29

**10 PHARMACOVIGILANCE**

**10.1 Terms and Definitions**

The Medicines for Human Use (Clinical Trials) Regulations 2004 (SI 2004/1031) definitions:

**Adverse Event (AE)**

Any untoward medical occurrence in a subject to whom a medicinal product has been administered, including occurrences which are not necessarily caused by or related to that product.

**Adverse Reaction (AR)**

Any untoward and unintended response in a subject to an investigational medicinal product which is related to any dose administered to that subject.

**Unexpected Adverse Reaction (UAR)**

An adverse reaction the nature and severity of which is not consistent with the information about the medicinal product in question set out in:

In the case of a product with a marketing authorization, in the summary of product characteristics for that product

In the case of any other investigational medicinal product, in the investigator's brochure relating to the trial in question.

**Serious Adverse Event (SAE), Serious Adverse Reaction (SAR) or Suspected Unexpected Serious Adverse Reaction (SUSAR)**

Any adverse event, adverse reaction or unexpected adverse reaction, respectively, that:

results in death

is life-threatening* (subject at immediate risk of death)

requires in-patient hospitalisation or prolongation of existing hospitalisation** results in persistent or significant disability or incapacity, or

consists of a congenital anomaly or birth defect Other important medical events***

*‘life-threatening’ in the definition of ‘serious’ refers to an event in which the patient was at risk of death at the time of the event; it does not refer to an event which hypothetically might have caused death if it were more severe.

**Hospitalisation is defined as an inpatient admission, regardless of length of stay, even if the hospitalisation is a precautionary measure for continued observation. Hospitalisations for a pre-existing condition, including elective procedures that have not worsened, do not constitute an SAE.

***Other important medical events that may not result in death, be life-threatening, or require hospitalisation may be considered a serious adverse event/experience when, based upon appropriate medical judgment, they may jeopardise the subject and may require medical or surgical intervention to prevent one of the outcomes listed in this definition


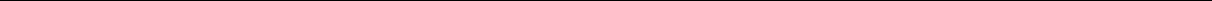


IRAS: 182752 57

TREAT Protocol v7.0 29/07/2020

**EudraCT Number:** 2015-002013-29

**10.2 Notes on Adverse Event Inclusions and Exclusions**

**10.2.1 Include**

An exacerbation of a pre-existing illness

An increase in frequency or intensity of a pre-existing episodic event/condition A condition (even though it may have been present prior to the start of the trial)

detected after trial drug administration

Continuous persistent disease or symptoms present at baseline that worsens following the administration of the study/trial treatment

Laboratory abnormalities that require clinical intervention or further investigation (unless they are associated with an already reported clinical event).

Abnormalities in physiological testing or physical examination that require further investigation or clinical intervention

Injury or accidents

**10.2.2 Do Not Include**

Medical or surgical procedures- the condition which leads to the procedure is the adverse event

Pre-existing disease or conditions present before treatment that do not worsen Situations where an untoward medical occurrence has occurred e.g. cosmetic

elective surgery

Overdose of medication without signs or symptoms

The disease being treated or associated symptoms/signs unless more severe than expected for the patient’s condition

**10.2.3 Reporting of Pregnancy**

Females of childbearing potential will be tested for pregnancy as part of the trial screening visit and baseline visit. Any pregnancy that occurs (including any male participant that has a partner that becomes pregnant) during the study should be reported using a pregnancy CRFto the CTRC within 24 hours of the site becoming aware of its occurrence and the participant should be instructed immediately to stop taking the study drug. All pregnancies that occur during treatment need to be followed up until after the outcome using the pregnancy CRF. Consent to report information regarding these pregnancy outcomes should be obtained from the mother prior to completion and faxing of the pregnancy CRF. Any SAE experienced during pregnancy must be reported on the SAE form as appropriate (section 10.1).

The investigator should contact the participant to discuss the risks of continuing with the pregnancy and the possible effect to the foetus. Appropriate Obstetric care should be arranged.

The CTRC will report all pregnancies to the trial Sponsor. Any pregnancies that results in complication will be reported to the MHRA (UK sites) or HPRA (Dublin site).

Data on the patient and the outcome of the pregnancy must be collected. This data will be entered in the annual Developmental Safety Update Reports and the final Clinical Study Report.


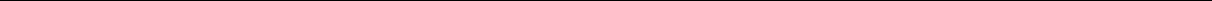


IRAS: 182752 58

TREAT Protocol v7.0 29/07/2020

**EudraCT Number:** 2015-002013-29

**10.3 Notes Severity / Grading of Adverse Events**

The assignment of the severity/grading should be made by the investigator responsible for the care of the participant using the definitions below.

Regardless of the classification of an AE as serious or not, its severity must be assessed according to medical criteria alone using the following categories:

**Mild**: does not interfere with routine activities

**Moderate**: interferes with routine activities

**Severe**: impossible to perform routine activities

A distinction is drawn between serious and severe AEs. Severity is a measure of intensity (see above) whereas seriousness is defined using the criteria in section 10.1, hence, a severe AE need not necessarily be a Serious Adverse Event.

**10.4 Relationship to Trial Treatment**

The assignment of the causality should be made by the investigator responsible for the care of the participant using the definitions in table 9.

If any doubt about the causality exists the local investigator should inform the study coordination centre who will notify the Chief Investigator. In the case of discrepant views on causality between the investigator and others, the MHRA will be informed of both points of view.

**Table 10: Definitions of Causality**

| **Relationship** | **Description** |
| --- | --- |
| **Unrelated** | There is no evidence of any causal relationship. N.B. An |
|  | alternative cause for the AE should be given |
|  |  |
| **Unlikely** | There is little evidence to suggest there is a causal relationship |
|  | (e.g. the event did not occur within a reasonable time after |
|  | administration of the trial medication). There is another |
|  | reasonable explanation for the event (e.g. the participant’s clinical |
|  | condition, other concomitant treatment). |
|  |  |
| **Possibly** | There is some evidence to suggest a causal relationship (e.g. |
|  | because the event occurs within a reasonable time after |
|  | administration of the trial medication). However, the influence of |
|  | other factors may have contributed to the event (e.g. the |
|  | participant’s clinical condition, other concomitant treatments). |
|  |  |
| **Probably** | There is evidence to suggest a causal relationship and the |
|  | influence of other factors is unlikely. |
|  |  |
| **Almost certainly** | There is clear evidence to suggest a causal relationship and other |
|  | possible contributing factors can be ruled out. |
|  |  |

**10.5 Expectedness**

An AE whose causal relationship to the study drug is assessed by the investigator as “possibly”, “probably”, or “almost certainly” is an Adverse Drug Reaction.

All events judged by the investigator to be possibly, probably, or almost certainly related to the IMP, graded as serious and **unexpected** (see SPC for list of Expected Adverse Events) should be reported as a SUSAR.


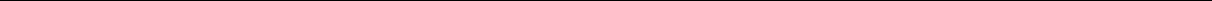


IRAS: 182752 59

TREAT Protocol v7.0 29/07/2020

**EudraCT Number:** 2015-002013-29

**10.6 Follow-up After Adverse Events**

All adverse events should be followed until satisfactory resolution or until the investigator responsible for the care of the participant deems the event to be chronic or the patient to be stable.

When reporting SAEs and SUSARs the investigator responsible for the care of the participant should apply the following criteria to provide information relating to event outcomes: resolved; resolved with sequelae (specifying with additional narrative); not resolved/ongoing; ongoing at final follow-up; fatal or unknown.

**10.7 Reporting Procedures**

All adverse events should be reported from randomisation until 4 weeks after treatment cessation. Depending on the nature of the event the reporting procedures below should be followed. Any questions concerning adverse event reporting should be directed to the CTRC in the first instance. A flowchart is given below to aid in determining reporting requirements.

Should a trial participant be administered one of the trial treatments (ciclosporin or methotrexate) as part of routine clinical care following cessation of their trial treatment then safety reporting procedures should be followed as described here.

**10.7.1 Non serious ARs/AEs**

All such events, whether expected or not, should be recorded on an Adverse Event Form, which should be transmitted to the CTRC within seven days of the form being due.

**10.7.2 Serious ARs/AEs/SUSARs**

SARs, SAEs and SUSARs should be reported to the CTRC within 24 hours of the local site becoming aware of the event. The SAE form asks for the nature of event, date of onset, severity, corrective therapies given, outcome and causality. The responsible investigator should sign the causality of the event. Additional information should be sent within 5 days of the reaction has not resolved at the time of reporting.

The CTRC will pass on any SUSARs to Kings Health Partners Clinical Trials Office (KHP CTO) who will notify the MHRA of all SUSARs occurring during the study. The CTRC will notify the main REC of all SUSARs occurring during the study. The MHRA and main REC will be notified according to the following timelines; fatal and life-threatening within 7 days of notification and non-life threatening within 15 days. All investigators will be informed of all SUSARs occurring throughout the study. Local investigators should report any SUSARs and /or SAEs as required locally.


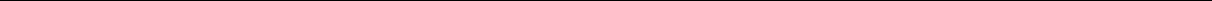


IRAS: 182752 60

TREAT Protocol v7.0 29/07/2020

**EudraCT Number:** 2015-002013-29

Adverse event


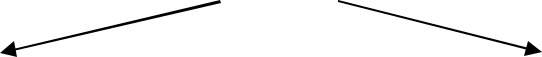


| Unrelated/Unlikely | |  | Possibly/Probably/Almost Certainly related | | |  |
| --- | --- | --- | --- | --- | --- | --- |
| Serious |  | Not serious | Serious |  | Not serious |  |
| Unexpected | Expected | Unexpected Expected | Unexpected | Expected | Unexpected | Expected |


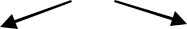

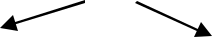

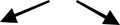

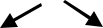

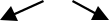

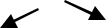

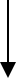

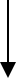

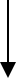

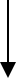

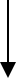

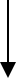

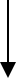

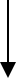


| **Unexpected** | **Expected** | Complete AE CRF and |
| --- | --- | --- |
| **SAE report** | **SAE** | submit as per routine |
| **to CTRC** | **report to** | schedule |
| **within 24** | **CTRC** |  |
| **hours** | **within 24** |  |
|  | **hours** |  |

| **SUSAR** | **SAR** | Unexpected | Expected AR. |
| --- | --- | --- | --- |
| **report to** | **report to** | AR. Report | Report on AE |
|  |  |  |  |
| **CTRC** | **CTRC** | on AE CRF | CRF and |
|  |  |  |  |
| **within 24** | **within 24** | and submit | submit as per |
|  |  |  |  |
| **hours** | **hours** | as per routine | routine |
|  |  |  |  |
|  |  | schedule | schedule |

**10.8 Responsibilities – Investigator**

The Investigator is responsible for reporting all AEs that are observed or reported during the study, regardless of their relationship to study product.

All SAEs must be reported immediately (but no later than 24 hours) by the investigator to the CTRC on an SAE form unless the SAE is specified in the protocol as not requiring immediate reporting. All other adverse events should be reported on the regular progress/follow-up reports.

**Minimum information required for reporting:**

Study identifier

Study centre

Patient number

A description of the event

Date of onset

Current status

Whether study treatment was discontinued

The reason why the event is classified as serious Investigator assessment of the association between the event and study treatment

1. The SAE form should be completed by a designated investigator, a physician named on the ‘signature list and delegation of responsibilities log’ as responsible for reporting SAEs and making trial related medical decisions. The investigator should assess the SAE for the likelihood that it is a response to the investigational medicinal product. In the absence of the designated investigator the form should be completed and signed by an alternative member of the research site trial team and submitted to the CTRC. As soon as possible thereafter the responsible investigator should check the SAE form, make amendments as appropriate, sign and re-send to the CTRC. The initial report shall be followed by detailed reports as appropriate.


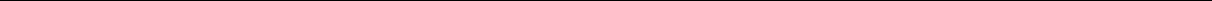


IRAS: 182752 61

TREAT Protocol v7.0 29/07/2020

**EudraCT Number:** 2015-002013-29

1. When submitting an SAE to the CTRC research sites should also telephone the appropriate trial co-ordinator/data manager to advise that an SAE report has been submitted.
2. Send the SAE form by fax (within 24 hours or next working day) to the CTRC
3. The responsible investigator must **notify** their R&D department of the event (as per standard local governance procedures).
4. In the case of an SAE the subject must be followed-up until clinical recovery is complete and laboratory results have returned to normal, or until the event has stabilised. Follow-up may continue after completion of protocol treatment if necessary.
5. Follow-up information is noted on another SAE form by ticking the box marked ‘follow-up’ and faxing to the CTRC as information becomes available. Extra, annotated information and/or copies of test results may be provided separately.
6. The patient **must** be identified by trial number, date of birth and initials only. The patient’s name **should not** be used on any correspondence.

**10.8.1 Maintenance of Blinding**

Investigators at sites will be aware of participants allocation as are the dermatology research nurses who look at the trial patients on a day-to-day basis. However, the blinded assessor who will perform the severity assessments will be blinded to the trial allocation.

**10.9** **Responsibilities – CTRC and KHP CTO**

The CTRC is undertaking duties delegated by the trial co-sponsor/s, Guys and St Thomas’ Foundation Trust and King’s College London and will forward completed SAE reports of SUSARs to KHP CTO. KHP CTO is responsible for the reporting of SUSARs and other SARs to the regulatory authorities (MHRA, competent authorities of other European member states in which the trial is taking place) and, the CTRC is responsible for the reporting to the research ethics committees as follows:

SUSARs which are fatal or life-threatening must be reported not later than 7 days after the CTRC is first aware of the reaction. Any additional relevant information must be reported within a further 8 days.

SUSARs that are not fatal or life-threatening must be reported within 15 days of the CTRC first becoming aware of the reaction.

A list of all SARs (expected and unexpected) must be reported annually.

It is recommended that the following safety issues should also be reported in an expedited fashion:

An increase in the rate of occurrence or a qualitative change of an expected serious adverse reaction, which is judged to be clinically important;

Post-study SUSARs that occur after the patient has completed a clinical trial and are notified by the investigator to the sponsor;

New events related to the conduct of the trial or the development of the IMPs and likely to affect the safety of the subjects, such as:

- 1. A SAE which could be associated with the trial procedures and which could modify the conduct of the trial;


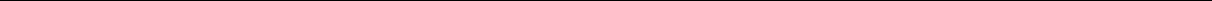


IRAS: 182752 62

TREAT Protocol v7.0 29/07/2020

**EudraCT Number:** 2015-002013-29

- 1. A significant hazard to the subject population, such as lack of efficacy of an IMP used for the treatment of a life-threatening disease;
  2. A major safety finding from a newly completed animal study (such as carcinogenicity).
  3. Any anticipated end or temporary halt of a trial for safety reasons and conducted with the same IMP in another country by the same sponsor;

Recommendations of the Independent Data and Safety Monitoring Committee, if any, where relevant for the safety of the subjects.

Staff at the CTRC will liaise with the Chief Investigator (or designated other specified in the protocol) who will evaluate all SAEs received for seriousness, expectedness and causality. Investigator reports of suspected SARs will be reviewed immediately and those that are SUSARs identified and forwarded to Kings Health Partners Clinical Trials Office for onward reporting to the regulatory authority. The CTRC will be responsible for onward reporting to the MREC of any SUSARs. The causality assessment given by the Local Investigator at the hospital cannot be overruled and in the case of disagreement, both opinions will be provided with the report.

The PIs at all institutions participating in the trial will be notified of any SUSARs.

Patient safety incidents that take place in the course of research should be reported to the National Patient Safety Agency (NPSA) by each participating NHS Trust in accordance with local reporting procedures.

**10.9.1 Safety reports**

Safety reports will be generated during the course of the trial which allows for monitoring of SAE and ADR reporting rates across sites. The CTRC/KHP CTO will send developmental safety update reports containing a list of all SARs to regulatory authorities and MREC. Any concerns raised by the IDSMC or inconsistencies noted at a given site may prompt additional training at sites, with the potential for the CTRC/KHP CTO to carry out site visits if there is suspicion of unreported AEs in patient case notes. Additional training will also be provided if unacceptable delay in safety reporting timelines. If any safety reports identify issues that have implications for the safety of trial participants, the PIs at all institutions participating in the trial will be notified.

**10.10 Contact Details and Out-of-hours Medical Cover**

The patient diary will direct patients to contact the research team at their site should medical advice be required during office hours. If medical advice is required outside of office hours then the participant will be advised to seek usual medical advice. The reviewing physician will be able to make the decision to cease the trial medication if the participant is receiving it at the time.


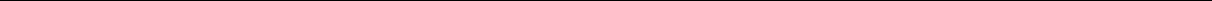


IRAS: 182752 63

TREAT Protocol v7.0 29/07/2020

**EudraCT Number:** 2015-002013-29

**11 ETHICAL CONSIDERATIONS**

**11.1 Ethical Considerations**

The study will abide by the principles of the World Medical Association Declaration of Helsinki (1996), the principles of GCP and in accordance with all applicable regulatory requirements, including but not limited to the Research Governance Framework and the Medicines for Human Use (Clinical Trial) Regulations 2004, as amended in 2006 and any subsequent amendments.

This protocol and related documents will be submitted for review to the REC, and to the MHRA for Clinical Trial Authorisation.

The Chief Investigator will submit a final report at conclusion of the trial to the KHP-CTO (on behalf of the Sponsor), the REC and the MHRA within the timelines defined in the Regulations.

There are no major ethical issues however the following points have been considered:

**Study interventions:** Whilst both CyA and MTX have potential side effects, both medications are used in standard NHS practice. Full information about possible risks and benefits of both medications will be provided to parents and participants and data on adverse events will be collected and monitored throughout the trial. In addition, the treatment of severe atopic eczema in participants will not be compromised by participation in the trial. The precise risks and benefits of participating in the study will be outlined in patient information sheets, to be formulated with service user involvement.

**Blood and other tests:** The risks of taking blood include temporary discomfort from the needle in the arm, bleeding, bruising, swelling at the needle site and, in rare instances, infection. None of the other investigations and procedures, including BP measurement, urine testing, and tape stripping are uncomfortable or harmful.

**Ongoing treatment after the study has been completed:** All participants will be able to receive further systemic immuno-suppressive therapy via the NHS if required, after the study has been completed.

**Consent in paediatric population:** Children up to the age of 16 will be eligible for enrolment in the trial and so age-appropriate Participant Information Sheets (PISs) will be prepared in line with current guidelines. Please see section 11.2 for further details.

**11.2 Ethical Approval**

The trial protocol will not be initiated until it has received the favourable opinion of a Multi-centre Research Ethics Committee (MREC). Subsequent to this, it must also undergo independent review at R&D offices at the R&D offices at participating sites. The local R&D office should be sent the appropriate site specific information form complete with the necessary authorisation signatures, plus any other documentation requested for review. A copy of local Research & Development (R&D) approval should be forwarded to CTRC before the site is initiated and patients recruited.


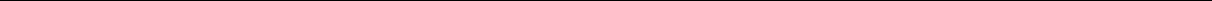


IRAS: 182752 64

TREAT Protocol v7.0 29/07/2020

**EudraCT Number:** 2015-002013-29

Children from the age of 2 to 16 will be eligible for enrolment in the trial. Proxy consent from the parent or legally acceptable representative should be obtained prior to each patient participating in the trial, after a full explanation has been given of the treatment options, including the conventional and generally accepted methods of treatment. Age and stage-of development specific Patient Information and Consent Forms (PISC) should also be implemented and patient assent obtained where appropriate. The right of the parent/ legal representative to refuse consent for the minor to participate in the trial without giving reasons must be respected. After the patient has entered the trial, the clinician must remain free to give alternative treatment to that specified in the protocol, at any stage, if he/she feels it to be in the best interest of the patient. However, the reason for doing so should be recorded and the patient will remain within the trial for the purpose of follow-up and data analysis. Similarly, the parent/legal representative of the patient remains free to withdraw the patient at any time from the protocol treatment and trial follow-up without giving reasons and without prejudicing the further treatment of the minor.

For children of school age, efforts will be made to arrange appointments outside of school hours whenever possible.

**11.3 Informed Consent Process**

Informed consent is a process initiated prior to an individual agreeing to participate in a trial and continues throughout the individual’s participation. Informed consent is required for all patients participating in CTRC coordinated trials. In obtaining and documenting informed consent, the investigator should comply with applicable regulatory requirements and should adhere to GCP and to the ethical principles that have their origin in the Declaration of Helsinki.

Discussion of objectives, risks and inconveniences of the trial and the conditions under which it is to be conducted are to be provided to patients by medically qualified physicians with experience in obtaining informed consent. Where appropriate, age-and-stage-of-development appropriate Patient Information and Consent forms, describing in detail the trial interventions/products, trial procedures and risks will be approved by an independent ethical committee (IEC) and the patient (parent/legal representative in the case of minors) will be asked to read and review the document. Upon reviewing the document, the investigator/medically qualified physician will explain the research study to the patient (parent/legal representative in the case of minors). This information will emphasise that participation in the trial is voluntary and that the participant may withdraw from the trial at any time and for any reason. All participants will be given opportunity to ask any questions that may arise, should have the opportunity to discuss the study with their surrogates and time to consider the information prior to agreeing to participate. A contact point where further information about the trial may be obtained will be provided.

The patient (parent or legal representative in the case of minors) will then sign and date the informed consent document. Both the person taking consent (medically qualified physician on the delegation log) and the participant must personally sign and date the form. A copy of the informed consent document will be given to the patient/their legally acceptable representative for their records. The original copy will be filed in the Investigator Site File,


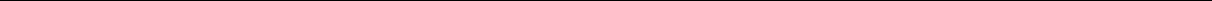


IRAS: 182752 65

TREAT Protocol v7.0 29/07/2020

**EudraCT Number:** 2015-002013-29

and a further copy will go in the participants medical notes. One final copy of the consent form should be sent to the CTRC.

Adequate time to consider trial entry will be allowed before written consent of the participants/parent/legal representative will be obtained by the responsible clinician. A patient and/or parent/legal guardian should be provided with the study information during their outpatient appointment and may be approached for consent during this visit if considered appropriate. Where the patient and/or parent/legal guardian feel that they have adequate information regarding the trial and are happy to proceed then consent can be sought and screening assessments undertaken on the same day as initial contact and provision of information.

The patient may, without being subject to any resulting detriment, withdraw from the trial at any time by revoking the informed consent (Similarly, the parent or legal representative may withdraw a minor under the same conditions). The rights and welfare of the patients will be protected by emphasising to them that the quality of medical care will not be adversely affected if they decline to participate in this study.

**11.3.1 Consent for 16 year olds**

A participant involved in the study who reaches the age of 16 (and is therefore no longer a minor) should be approached to provide consent as a competent adult at their next scheduled visit after their 16th birthday.

**11.3.2 Assent in minors**

If capable, and under appropriate circumstances, minors should be approached to provide assent by a delegated clinician with experience with minors. Age-and-state-of-development IEC-approved Patient Information Sheet and Assent forms, describing (in simplified terms) the details of the trial intervention/product, trial procedures and risks should be used. The minor should personally write their name and date the assent form, which is then signed by the parent/legal representative and the researcher.

Assent forms do not substitute for the consent form signed by the patient’s legally acceptable representative. Assent should be taken where appropriate and documented in the patient notes, however the absence of assent does not exclude the patient provided consent has been obtained from the parent/legal representative.

**11.4 Study Discontinuation**

In the event that the study is discontinued, it would be possible for patients to continue on the medication prescribed at their site under the NHS, but this would be at the discretion of the clinician responsible for their care.


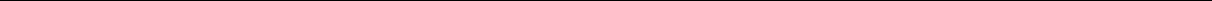


IRAS: 182752 66

TREAT Protocol v7.0 29/07/2020

**EudraCT Number:** 2015-002013-29

**12 REGULATORY APPROVAL**

This trial falls within the remit of the EU Directive 2001/20/EC, transposed into UK law as the UK Statutory Instrument 2004 No 1031: Medicines for Human Use (Clinical Trials) Regulations 2004 as amended. This trial has been registered with the MHRA and HPRA (Ireland) and has been granted a Clinical Trial Authorisation (CTA).

IRAS: 182752 67

TREAT Protocol v7.0 29/07/2020

**EudraCT Number:** 2015-002013-29

**13 TRIAL MONITORING**

Trial monitoring is carried out to ensure that the rights and well-being of human participants are protected during the course of a clinical trial. A risk assessment is performed for each trial coordinated by the CTRC and KHP CTO to determine the level and type of monitoring required for specific hazards. The nature and extent of monitoring will be specific to the individual trial.

Trial Oversight Committees related to the monitoring of the trial are detailed in section [16.](#page74)

**13.1 Risk Assessment**

In accordance with the CTRC SOP TM005 this trial is undergoing a risk assessment, to be completed in partnership between:

Representative/s of the Trial Sponsor Chief Investigator

Trial Coordinator and supervising Trial Manager Trial Statistician and supervising Statistician

Information Systems team CTRC Director

Guidance issued by the MRC, Department of Health and the MHRA on risk-adapted approaches to the management of CTIMPs (Ref) propose a three level categorisation for the potential risk associated with the IMP, assigned according to the following categories:

***Type A*** *‘no higher than that of standard medical care’;*

***Type B*** *‘somewhat higher than that of standard medical care’;*

***Type C*** *‘markedly higher than that of standard medical care’.*

The TREAT trial falls into the second category (Type B). This level of risk informs the risk assessment, regulatory requirements, nature and extent of the monitoring, and the management processes used in the trial.

**13.2 Source Documents**

***Source data****: All information in original records and certified copies of original records of clinical findings, observations, or other activities in a clinical trial necessary for the reconstruction and evaluation of the trial. Source data are contained in source documents (original records or certified copies). (ICH E6, 1.51).*

***Source documents****: Original documents, data, and records (e.g., hospital records, clinical and office charts, laboratory notes, memoranda, subjects diaries or evaluation checklists, pharmacy dispensing records, recorded data from automated instruments, copies or transcriptions certified after verification as being accurate copies, microfiches, photographic negatives, microfilm or magnetic media, x-rays, subject files, and records kept at the pharmacy, at the laboratories and at medico-technical departments involved in the clinical trial). (ICH E6, 1.52).*

IRAS: 182752 68

TREAT Protocol v7.0 29/07/2020

**EudraCT Number:** 2015-002013-29

In order to resolve possible discrepancies between information appearing in the CRF and any other patient related documents, it is important to know what constitutes the source document and therefore the source data for all information in the CRF. The data that is to be recorded in the CRF should be consistent and verifiable with source data in source documents *other* than the CRF (e.g. medical record, laboratory reports and nurses’ notes). For the data where no prior record exists and which is recorded directly in the CRF, the CRF will be considered the **source document**, unless otherwise indicated by the investigator.

In addition to the above, date(s) of conducting informed consent (plus assent where appropriate and if taken) process including date of provision of patient information, registration number, randomisation number and the fact that the patient is participating in a clinical trial (including possible treatment arms) should be added to the patient’s medical record chronologically, i.e. when treatment is allocated to the patient.

Investigators and the institutions will permit trial-related monitoring, audits, REC review, and regulatory inspections (where appropriate) by providing direct access to source data and other documents (i.e. patients’ case sheets, blood test reports, X-ray reports, histology reports etc).

**13.3 Data Capture Methods**

**13.3.1 Case Report Forms**

The study case report form (CRF) is the primary data collection instrument for the study. All data requested on the CRF must be recorded. CTRC will provide participating sites with CRFs and guidance on how the CRF should be completed. All missing data must be explained. If a space on the CRF is left blank because the procedure was not done or the question was not asked, write “N/D”. If the item is not applicable to the individual case, write “N/A”. Or if the data item is un-known, write “NK”. If a data item has not been recorded on source data then write ‘NR’. All entries should be printed legibly in black ink. If any entry error has been made, to correct such an error, draw a single straight line through the incorrect entry and enter the correct data above it. All such changes must be initialled and dated. DO NOT ERASE OR WHITE OUT ERRORS. For clarification of illegible or uncertain entries, print the clarification above the item, then initial and date it. All completed CRFs, patient diaries and questionnaires should be photocopied by the participating site prior to sending originals to the CTRC.

**13.4 Central Monitoring**

Data stored at CTRC will be checked for missing or unusual values (range checks) and checked for consistency within participants over time. Any suspect data will be returned to the site in the form of data queries. Data query forms will be produced at the CTRC from the trial database and sent either electronically or through the post to a named individual (as listed on the site delegation log). Sites will respond to the queries providing an explanation/resolution to the discrepancies and return the data query forms to CTRC. The forms will then be filed along with the appropriate CRFs and the appropriate corrections made on the database. There are a number of monitoring features in place at the CTRC to ensure reliability and validity of the trial data, to be detailed in the trial monitoring plan.

IRAS: 182752 69

TREAT Protocol v7.0 29/07/2020

**EudraCT Number:** 2015-002013-29

**13.5 Clinical Site Monitoring**

In order to perform their role effectively, Kings Health Partners Clinical Trials Office, the trial coordinator (or monitor) and persons involved in Quality Assurance and Inspection may need direct access to primary data, e.g. patient records, laboratory reports, appointment books, etc. Since this affects the patient’s confidentiality, this fact is included on the Parent Information Sheet and Informed Consent Form.

**13.5.1 Confidentiality**

Individual participant medical information obtained as a result of this study is considered confidential and disclosure to third parties is prohibited with the exceptions noted below. Case report forms will be labelled with the patient’s initials and unique trial screening and/or randomisation number. Medical information may be given to the participant’s medical team and all appropriate medical personnel responsible for the participant’s welfare.

The CTRC will be undertaking activities requiring the transfer of identifiable data: Verification that appropriate informed consent is obtained will be enabled by the provision of copies of participant’s signed informed consent/assent forms being supplied to the CTRC by recruiting centres, which requires that name data will be transferred to the CTRC. This transfer of identifiable data is disclosed in the PISC.

The CTRC will preserve the confidentiality of participants taking part in the study and The University of Liverpool is registered as a Data Controller with the Information Commissioners Office.

**13.5.2 Quality Assurance and Control**

QA includes all the planned and systematic actions established to ensure the trial is performed and data generated, documented/recorded and reported in compliance with applicable regulatory requirements. QC includes the operational techniques and activities done within the QA system to verify that the requirements for quality of the trial-related activities are fulfilled. The level and nature of monitoring will be described in the trial monitoring plans, which will be finalised upon completion of the trial risk assessment. To ensure the integrity of the data the following policies will be observed:

Data will be evaluated for compliance with protocol and accuracy in relation to source documents

The study will be conducted in accordance with procedures identified in the protocol. The Principal Investigator and RN for each site will attend the training visit, which will incorporate elements of trial specific training necessary to fulfil the requirements of

the protocol.

A greenlight checklist will be completed by the trial coordinator/KHP CTO to verify appropriate approvals and documentation are in place prior to initiation of a site and the relevant personnel have received trial specific training

The CTRC will monitor screening, recruitment and withdrawal rates between sites and report to the TMG

Regular QC checks will be performed on data already inputted to ensure data entered is of a high standard

Independent oversight of the study will be provided by the Independent Data and Safety Monitoring Committee and the Trial Steering Committee

IRAS: 182752 70

TREAT Protocol v7.0 29/07/2020

**EudraCT Number:** 2015-002013-29

**13.6 Records Retention**

The investigator at each investigational site must make arrangements to store the essential trial documents, (as defined in Essential Documents for the Conduct of a Clinical Trial (ICH E6, Guideline for Good Clinical Practice)) including the Investigator Site File and Pharmacy Site File, until the Clinical Trials Unit informs the investigator that the documents are no longer to be retained, or for a maximum period of 15 years (whichever is soonest).

In addition, the investigator is responsible for archiving of all relevant source documents so that the trial data can be compared against source data after completion of the trial (e.g. in case of inspection from authorities).

The investigator is required to ensure the continued storage of the documents, even if the investigator, for example, leaves the clinic/practice or retires before the end of required storage period. Delegation must be documented in writing.

The CTRC undertakes to store originally completed CRFs for the same period, except for source documents pertaining to the individual investigational site, which are kept by the investigator only. The CTRC undertakes to archive as per their contractual requirement; the documents will be archived in compliance with the principles of GCP. All electronic CRFs and trial data will be archived onto an appropriate media for long term accessible storage. Hard copies of data will be boxed and transferred to specially renovated, secure, premises where unique reference numbers are applied to enable confidentiality, tracking and retrieval.

IRAS: 182752 71

TREAT Protocol v7.0 29/07/2020

**EudraCT Number:** 2015-002013-29

**14 INDEMNITY**

The TREAT trial is sponsored by Guys and St Thomas’ NHS Foundation Trust and Kings College London and co-ordinated by the CTRC in the University of Liverpool.

Kings College London has clinical trial insurance and professional indemnities in place to cover its liabilities in regards to any work undertaken by its staff in the course of their employment at the University. As this is an investigator-initiated study, the Association of the British Pharmaceutical Industry (ABPI) guidelines for patient compensation by the pharmaceutical industry do not apply.

The Guys and St Thomas’ NHS Foundation Trust does not hold insurance against claims for compensation for injury caused by participation in a clinical trial and they cannot offer any indemnity. As this is an investigator-initiated study, The Association of the British Pharmaceutical Industry (ABPI) guidelines for patient compensation by the pharmaceutical industry do not apply. However, in terms of liability, NHS Trust and Non-Trust Hospitals have a duty of care to patients treated, whether or not the patient is taking part in a clinical trial, and they are legally liable for the negligent acts and omission of their employees. Compensation is therefore available in the event of clinical negligence being proven.

**Clinical negligence is defined as:**

“A breach of duty of care by members of the health care professions employed by NHS bodies or by others consequent on decisions or judgments made by members of those professions acting in their professional capacity in the course of their employment, and which are admitted as negligent by the employer or are determined as such through the legal process”.

IRAS: 182752 72

TREAT Protocol v7.0 29/07/2020

**EudraCT Number:** 2015-002013-29

**15 FINANCIAL ARRANGEMENTS**

This trial is funded by the MRC-NIHR Efficacy and Mechanism Evaluation (EME) Board of the Department of Health. Contractual agreements will be in place between the sponsor and collaborating sites that will incorporate financial agreements.

**15.1 Collaborating Centre Payments**

Collaborating/recruiting centres will receive a per patient payment.

**15.2 Research Team**

As the study is funded by the MRC-NIHR EME, it will be automatically adopted onto the NIHR portfolio, which will allow trusts to apply to their comprehensive local research network for service support costs as required.

IRAS: 182752 73

TREAT Protocol v7.0 29/07/2020

**EudraCT Number:** 2015-002013-29

**16 TRIAL COMMITTEES**

**16.1 Trial Management Group (TMG)**

A Trial Management Group (TMG) will be formed comprising the Chief Investigator, other lead investigators (clinical and non-clinical) and members of the Clinical Trials Unit. The TMG will be responsible for the day-to-day running and management of the trial and will meet approximately 3 times a year. Refer to the TMG terms of reference and trial oversight committee membership document for further details.

**16.2 Trial Steering Committee (TSC)**

The Trial Steering Committee consists of an independent chairperson, two independent experts in the field of dermatology, a paediatrician, a medical statistician, two Patient and Public Involvement representative and the Chief Investigator or delegated representative. The role of the TSC is to provide overall supervision for the trial and provide advice through its independent Chairman. The ultimate decision for the continuation of the trial lies with the TSC. Refer to the TSC terms of reference and trial oversight committee membership document for further details.

**16.3 Independent Data and Safety Monitoring Committee (IDSMC)**

The independent Data and Safety Monitoring Committee (IDSMC) consists of an independent chairperson, plus 2 independent members: one who is an expert in the field of dermatology and one who is an expert in medical statistics.

The IDSMC will be responsible for reviewing and assessing recruitment, interim monitoring of safety and effectiveness, trial conduct and external data. The IDSMC will first convene prior to the start of recruitment and will then define frequency of subsequent meetings (at least annually). Details of monitoring are provided in section 9.

The IDSMC will provide a recommendation to the Trial Steering Committee concerning the continuation of the study. Refer to the IDSMC charter and trial oversight committee membership document for further details.

IRAS: 182752 74

TREAT Protocol v7.0 29/07/2020

**EudraCT Number:** 2015-002013-29

**17 PUBLICATION**

The results from different centres will be analysed together and published as soon as possible. Individual Clinicians must undertake not to submit any part of their individual data for publication without the prior consent of the Trial Management Group.

The Trial Management Group will form the basis of the Writing Committee and advise on the nature of publications. The Uniform Requirements for Manuscripts Submitted to Biomedical Journals [(http://www.icmje.org/)](http://www.icmje.org/) will be respected. All publications shall include a list of participants, and if there are named authors, these should include the trial’s Chief Investigator(s), Statistician(s) and Trial Manager(s) involved at least. If there are no named authors (i.e. group authorship) then a writing committee will be identified that would usually include these people, at least. The ISRCTN allocated to this trial should be attached to any publications resulting from this trial.

The members of the TSC and IDSMC should be listed with their affiliations in the Acknowledgements/Appendix of the main publication.

IRAS: 182752 75

TREAT Protocol v7.0 29/07/2020

**EudraCT Number:** 2015-002013-29

**18 PROTOCOL AMENDMENTS**

**18.1 Version 2 (08/10/2015)**

page 2 & 7 – change from Senior Lecturer to Reader for Dr Carsten Flohr

page 22 – change to exclusion criteria 7 to include males and to say that an acceptable method of contraception must be used for 6 months after the last dose of study drug page 23 – addition of the following to the exclusion criteria:

Receiving treatment with medicines that are substrates for the multidrug efflux transporter P-glycoprotein or the organic anion transporter proteins (OATP) for which elevated plasma concentrations are associated with serious and/or life-threatening events; this includes bosentan, dabigatran etexilate and aliskiren.

Receiving treatment with products containing *Hypericum perforatum* (St. John's wort)

Receiving oral treatment with tacrolimus

Receiving oral treatment with everolimus and sirolimus Receiving oral treatment with lercanidipine

page 26 – section 6.5.2 has been amended to say that patients who develop an unacceptable toxicity based on the investigator’s judgement will be withdrawn. page 31 – text added to explain the dosing regimen for methotrexate

page 34-36 – Details have been added to section 7.7.2 to describe any medications that are not permitted and any precautions that are required to be taken with regards to administration of concomitant medications.

**18.2 Version 3.0 23/10/2015**

Page 2 – addition of qualifications for Carsten Flohr and Ashley Jones.

Page 5 – title altered to ‘Professor’ for Leonie Taams

Page 11 – Progressive Multifocal Leukoencephalopathy (PML), Tuberculosis (TB) and Thiopurine Methyltransferase (TPMT) added to Glossary

Page 15 – schematic of study design altered to reflect new schedule and figures for recruitment corrected

Page 16-17 – Further details added on why we are comparing ciclosporin with methotrexate. Page 18-20 – Further detail on risk:benefit ratio of trial interventions added

Page 24 – Exclusion to reflect abnormal chest x-ray added to the exclusion criteria Page 27 – Details on steps in place to ensure blinding is maintained added

Page 29 – Details added on the drug dosing for the trial Investigational Medicinal Products and information regarding chest x-rays required for patients with a risk of TB

Page 31 – Information on when occasional monitoring of ciclosporin is recommended added Page 32-33 – Further details on precautions to be taken when prescribing and dispensing methotrexate added

Page 34 – ‘New or worsening unexplained dyspnoea or cough’ added to the monitoring parameters for Methotrexate

Page 36 – Information added with regards to avoiding ibuprofen whilst taking methotrexate and management of this if it occurs.

Page 39 – window added for week 1 and 2.

Page 40 – Table of study procedures altered to reflect new schedule.

Page 41-45 – Visit summary altered to reflect new schedule

Page 45 – Details on what is recorded in patient diary added

Page 45 – Procedures for assessing safety updated to reflect new schedule Page 46 – Details on what is recorded in patient diary added Page 46 – ‘intervention vs control’ changed to ‘MTX vs CyA’

IRAS: 182752 76

TREAT Protocol v7.0 29/07/2020

**EudraCT Number:** 2015-002013-29

Page 59 – Patient information leaflets changed to patient information sheets

| **18.3** | **Version 4.0 17/10/2016** | | |
| --- | --- | --- | --- |
|  |  |  |  |
| **Page** |  | **Section** | **Change made** |
| **number** |  |  |  |
| Front page |  | NA | EME logo removed |
| Front page |  | NA | Addition of ISRCTN number added |
| Page 5 |  | NA | Contact details for the KCL Bio Bank amended |
| Page 11 |  | NA | Child Health Utility – Nine Dimensions added to Glossary |
| Page 12 |  | 1 | Addition of REC number to protocol summary |
| Page 12 |  | 1 | Addition of oral solution to intervention section |
| Page 12 |  | 1 | Addition of CHU-9D questionnaire used to assess cost |
|  |  |  | effectiveness of CyA vs MYA |
| Page 21 |  | 3.1 | Addition of confirmation of capacity and capability as part of centre |
|  |  |  | inclusion criteria |
| Page 23 |  | 5.1 | Inclusion 4. -clarification of inadequate clinical response to topical |
|  |  |  | corticosteroids |
| Page 23 |  | 5.1 | Inclusion 7 – addition of highly effective contraception including |
|  |  |  | examples |
| Page 23 |  | 5.1 | Inclusion 9 – deletion of criteria as patient no longer required to |
|  |  |  | swallow tablets/capsules as oral solutions are available |
| Page 23 |  | 5.2 | Exclusion 4 – clarification that values for both creatinine and eGFR |
|  |  |  | are required to assess renal function |
| Page 23 |  | 5.2 | Exclusion 6 – unit measurement for haemoglobin amended |
| Page 23 |  | 5.2 | Exclusion 7 – information on blood pressure measurement taken by |
|  |  |  | local GP or community nurse if unobtainable at screening or |
|  |  |  | baseline visits added. |
| Page 24 |  | 5.2 | Exclusion 13 – addition that chest x-rays can be requested by the |
|  |  |  | discretion of 'medically qualified physician' |
| Page 24 |  | 5.2 | Exclusion 21 – addition of 'patient and/or parent/guardian' |
| Page 25 |  | 6.1 | Further details added to clarify that consent and the screening visit |
|  |  |  | can be carried out on the same day |
| Page 25 |  | 6.1 | Screening log amended to ‘Consent & Eligibility log’ |
| Page 25 |  | 6.2 | Detail added to clarify that data from clinical assessments carried |
|  |  |  | out prior to consent for routine clinical purposes can be used for |
|  |  |  | trial eligibility |
| Page 26 |  | 6.3 | Addition of safety bloods collected prior to screening visit can be |
|  |  |  | used to assess eligibility at baseline visit |
| Page 26 |  | 6.3 | FLG genotyping sample collected at baseline visit |
| Page 27 |  | 6.4.2 | Removal of any reference to the blinded assessor being a research |
|  |  |  | nurse as the blinded assessment can be carried out by an |
|  |  |  | independent member of the research team who has no direct |
|  |  |  | involvement or contact with trial patient |
|  |  |  |  |

Page 29

6.5.3

Detail added that CTRC are to notify the laboratories if a patient has withdrawn from the trial and requested that their data not to be analysed and to be destroyed

IRAS: 182752 77

TREAT Protocol v7.0 29/07/2020

**EudraCT Number:** 2015-002013-29

| Page 30 | 7.1 | Addition of Methotrexate and Ciclosporin oral solution |
| --- | --- | --- |
| Page 30 | 7.1 | Addition of radiation exposure |
| Page 32 | 7.2.3 | Removal of reference to treatment failure as does not apply to the |
|  |  | trial |
| Page 32 | 7.2.3 | Further detail on missed treatment (Methotrexate) |
| Page 34-35 | 7.3.3 | Further detail on initial test dose and guidance for PI’s if safety |
|  |  | bloods at week 1 indicate a degree of toxicity to the MTX test dose |
|  |  | Increase of the number of weeks that a patient will remain on full |
|  |  | treatment dose |
|  |  | Removal of reference to treatment failure as does not apply to the |
|  |  | trial |
|  |  | Further detail on missed treatment (Ciclosporin) |
| Page 36 | 7.7.1 | Addition of pregnancy CRF |
| Page 37 | 7.7.2 | Removal of antibiotics for treatment of atopic eczema related skin |
|  |  | infections in the medications not permitted section |
| Page 40-42 | 8.1 | Removal of reference to deviations outside visit window discussed |
|  |  | with CI |
|  |  | Table of study procedures amended to reflect new schedule |
|  |  | Clarification that mechanistic blood samples are to be collected |
|  |  | from sites that can transport samples to the KCL bio bank by 4pm |
|  |  | on the same day |
|  |  | Clarification that AE and SAE data are to be collected until 4 weeks |
|  |  | after treatment has stopped |
| Pages 42-47 | 8.1.2 | Visit summary amended to reflect new schedule |
|  |  | Addition of unscheduled visit and data expected to be recorded |
|  |  | during visit |
|  |  | Addition of separate diary during follow up and that patients will be |
|  |  | provided with more than one diary to complete on occasions where |
|  |  | the study visits are longer than 4 weeks apart |
| Page 47 | 8.2 | Further detail added to second co-primary outcome |
| Page 48 | 8.3 | Detail added to clarify procedure if safety bloods are unobtainable |
|  |  | due to insufficient sample or patient refusal |
| Page 48 | 8.4.1 | Quality of life questionnaires separated into individual sections |
| Page 49 | 8.4.2 | Further detail added to health economic evaluation. |
| Page 50-52 | 8.4.3 | Removal of specified timeline for laboratory analysis to commence |
|  |  | in London and Dublin |
|  |  | Addition to the number of tape strips collected and clarification that |
|  |  | not all participating sites will be involved in the collection of tape |
|  |  | strips. |
|  |  | Clarification of the samples that require processing and storage |
|  |  | prior to transporting to the KCL bio bank and reference to the |
|  |  | sample manual |
| Page 56 | 10.2.3 | Clarification on reporting requirement to the MHRA/REC/HPRA for |
|  |  | pregnancies that result in complications. |
| Page 58 | 10.7.2 | Addition of ‘Unlikely’ to the adverse event flowchart |
| Page 64 | 11.3.1 | Addition of consent of a 16 year old who is no longer a minor |
|  |  | should be approached to provide consent as a competent adult at |
|  |  | their next scheduled visit after their 16th birthday. |
| Page 67 | 13.3.1 | Updated to remind participating sites to photocopy completed |
|  |  |  |

IRAS: 182752 78

TREAT Protocol v7.0 29/07/2020

**EudraCT Number:** 2015-002013-29

|  |  |
| --- | --- |
| Page 72 | 16.2 |
|  |  |
| NA | NA |
|  |  |

CRFS, questionnaires and diaries prior to sending the original copies to the CTRC

Further detail added to the members involved in the trial steering committee

Other minor typographical errors corrections and clarifications in order to ensure consistency made throughout

| **18.4** | **Version 5.0 29/06/2017** | | |
| --- | --- | --- | --- |
|  |  |  |  |
| **Page** |  | **Section** | **Change made** |
| **number** |  |  |  |
| 5 |  | NA | Contact details for Sponsor and trial management and monitoring |
|  |  |  | amended |
| 27 |  | 6.3 | Addition of pregnancy test during the baseline visit to confirm |
|  |  |  | patient eligibility at the point of randomisation (also noted in section |
|  |  |  | 7.7.1) |
| 27 |  | 6.3 | Addition of POEM baseline questionnaire used to capture POEM |
|  |  |  | data at baseline visit only |
|  |  |  |  |

1. 6.5.2Addition of adverse event reporting procedures for patients discontinued from trial treatment but remain to be treated by their clinician on one of the trial interventions

| 30 | 7.2.3 | Clarification to report events that meet the monitoring parameters |
| --- | --- | --- |
|  |  | (table 4) as adverse events (ciclosporin) |
| 31 | 7.2.3 | Clarification that a fall of eGFR >=20% is compared to the value |
|  |  | obtained at the screening visit (ciclosporin) |
| 31 | 7.2.3 | Clarification to the action taken if eGFR falls >=20% (ciclosporin) |
| 32 | 7.3.1 | Addition of 25mg/ml methotrexate prefilled pen |
| 32 | 7.3.2 | Further guidance on methotrexate doses |
| 33 | 7.3.3 | Clarification to report events that meet the monitoring parameters |
|  |  | (table 4) as adverse events. |
| 34 | 7.3.3 | Clarification that a fall of eGFR >=20% is compared to the value |
|  |  | obtained at the screening visit (methotrexate) |
| 34 | 7.3.3 | Clarification to the action taken if eGFR falls >=20% (methotrexate) |
| 41 | 8.1 | Table of study procedures amended to reflect new schedule |
| 42-46 | 8.1.2 | Visit summary amended to reflect new schedule |
|  |  |  |

1. 10.7Addition of adverse event reporting procedures for patients discontinued from trial treatment but remain to be treated by their clinician on one of the trial interventions

IRAS: 182752 79

TREAT Protocol v7.0 29/07/2020

**EudraCT Number:** 2015-002013-29

**18.5 Version 6.0 18/02/2019**

| **Page** | **Section** | **Change made** |
| --- | --- | --- |
| **number** |  |  |
| 4-5 | NA | Contact details for Sponsor and external laboratories amended |
| 7 | NA | Contact details for CI and trial nephrologist amended |
| 12 | 1 | Text added to refine co-primary outcome |
| 17 | 2.3 | Text added to refine the secondary endpoints |
| 25 | 6.3 | Addition of text highlighting importance of eligibility |
| 28 | 6.5.2 | Detail added to clarify collection of data for patients who |
|  |  | discontinue trial treatment |
| 32 | 7.2.3 | Further detail added to the monitoring parameter table, to allow for |
|  |  | temporary interruption to MTX |
| 35-36 | 7.3.3 | Further detail added to the monitoring parameter table, to allow for |
|  |  | temporary interruption to MTX |
| 43 | 8.1 | Table of study procedures amended to reflect that the FLG sample |
|  |  | can be collected at any visit following randomisation. |
| 44 | 8.1.2 | Text added to indicate which hospital collects tape striping and |
|  |  | mechanistic samples. |
| 48-49 | 8.2 | Further detail added for the justification of refining the co-primary |
|  |  | outcome |
| 49 | 8.3 | Reference to Creatinine, Cystatin C, and urine NAG as being |
|  |  | safety bloods removed. |
| 50-51 | 8.4.2 | Additional text added to allow research nurses to contact patients |
|  |  | in order to collect missing health resource use data. Addition of |
|  |  | GP questionnaire to collect health resource use data. |
| 51 | 8.4.3 | Clarification that mechanistic samples are to be collected from all |
|  |  | patients recruited at St Thomas’ Hospital. |
| 52 | 8.4.3 | Clarification that all research samples will be either sent to the |
|  |  | KCL bio bank or one of the external laboratories referenced in |
|  |  | pgs.4-6 |
| 54 | 9.4 & | Text added to refine the co-primary and secondary endpoints |
|  | 9.5 |  |
| 55 | 9.6 | Text added to refine the co-primary outcome |
| 55-56 | 9.7 | Text added to refine the analysis of the co primary outcome |
| NA | NA | Other minor typographical errors corrections and clarifications in |
|  |  | order to ensure consistency made throughout |

IRAS: 182752 80

TREAT Protocol v7.0 29/07/2020

**EudraCT Number:** 2015-002013-29

**18.6 Version 7.0 29/07/2020**

| **Page number** | **Section** | **Change made** |
| --- | --- | --- |
| 4-5 | NA | Contact details for external |
|  |  | laboratories amended |
| 43 | Table 9: Schedule of study | Clarification that mechanistic |
|  | procedures | samples are to be collected |
|  |  | from all patients recruited at |
|  |  | St Thomas’ Hospital. |
| 44 | 8.1.1 Assessments | Clarification that mechanistic |
|  |  | samples and tape strip |
|  |  | samples are to be collected |
|  |  | from all patients recruited at |
|  |  | St Thomas’ Hospital. |
| 50 | 8.4.2 Health Economics | Method of consent changed |
|  |  | to verbal consent |
| 71 | 13.6 Records Retention | Clarification to CTRC |
|  |  | archiving of trial data |

IRAS: 182752 81

TREAT Protocol v7.0 29/07/2020

**EudraCT Number:** 2015-002013-29

**19 REFERENCES**

1. Emerson RM, Williams HC, Allen BR. Severity distribution of atopic dermatitis in the community and its relationship to secondary referral. The British journal of dermatology. 1998;139(1):73-6.
2. Ben-Gashir MA, Seed PT, Hay RJ. Are quality of family life and disease severity related in childhood atopic dermatitis? Journal of the European Academy of Dermatology and Venereology : JEADV. 2002;16(5):455-62.
3. Kemp AS. Cost of illness of atopic dermatitis in children: a societal perspective. PharmacoEconomics. 2003;21(2):105-13.
4. McAleer MA, Flohr C, Irvine AD. Management of difficult and severe eczema in childhood. Bmj. 2012;345:e4770.
5. Flohr C, Irvine AD. Systemic therapies for severe atopic dermatitis in children and adults. The Journal of allergy and clinical immunology. 2013;132(3):774- e6.
6. Proudfoot LE, Powell AM, Ayis S, Barbarot S, Baselga Torres E, Deleuran M, et al. The European TREatment of severe Atopic eczema in children Taskforce (TREAT) survey. The British journal of dermatology. 2013;169(4):901-9.
7. El-Khalawany MA, Hassan H, Shaaban D, Ghonaim N, Eassa B. Methotrexate versus cyclosporine in the treatment of severe atopic dermatitis in children: a multicenter experience from Egypt. European journal of pediatrics. 2013;172(3):351-6.
8. Schram ME, Roekevisch E, Leeflang MM, Bos JD, Schmitt J, Spuls PI. A randomized trial of methotrexate versus azathioprine for severe atopic eczema. The Journal of allergy and clinical immunology. 2011;128(2):353-9.
9. Williams HC. Commentary: are methotrexate and azathioprine really equivalent for treating severe atopic eczema? Brit J Dermatol. 2012;166:705-6.
10. Schmitt J, Schmitt N, Meurer M. Cyclosporin in the treatment of patients with atopic eczema - a systematic review and meta-analysis. Journal of the European Academy of Dermatology and Venereology : JEADV. 2007;21(5):606-19.
11. Harper JI, Ahmed I, Barclay G, Lacour M, Hoeger P, Cork MJ, et al. Cyclosporin for severe childhood atopic dermatitis: short course versus continuous therapy. The British journal of dermatology. 2000;142(1):52-8.
12. Meggit SJ GJ, Reynolds NJ. Azathioprine dosed by thiopurine methyltransferase activity for moderate-to-severe atopic eczema: a double-blind, randomised controlled trial. . Lancet. 2006;367:839-46.
13. Masunaga Y, Ohno K, Ogawa R, Hashiguchi M, Echizen H, Ogata H. Meta-analysis of risk of malignancy with immunosuppressive drugs in inflammatory bowel disease. The Annals of pharmacotherapy. 2007;41(1):21-8.
14. Schmedt N, Andersohn F, Garbe E. Signals of progressive multifocal leukoencephalopathy for immunosuppressants: a disproportionality analysis of spontaneous reports within the US Adverse Event Reporting System (AERS). Pharmacoepidemiology and drug safety. 2012;21(11):1216-20.
15. Roekevisch E, Spuls PI, Kuester D, Limpens J, Schmitt J. Efficacy and safety of systemic treatments for moderate-to-severe atopic dermatitis: a systematic review. The Journal of allergy and clinical immunology. 2014;133(2):429-38.
16. Schmitt J RM, Pfennig A, Leopold K, Meurer M. Psychiatric comorbidity in adult eczema. British Journal of Dermatology. 2009;161(4):878-83.
17. Schmitt J BA, Meurer M. Atopic exzema in adulthood. Hautarzt. 2008;59(10):841-50.
18. Guidelines N. Atopic eczema in children from birth up to the age of 12 years. 2007.
19. Press JFCBNFoLBGaP.
20. Sunseri W HJ, Lerer T et. al. Retrospective cohort study of methotrexate use in the treatment of pedaitric Crohn's disease. Inflammatory Bowel Diseases. 2014;20(8):1341-5.

IRAS: 182752 82

TREAT Protocol v7.0 29/07/2020

**EudraCT Number:** 2015-002013-29

1. Klein A KI, Foeldvari I et al. Efficacy and safety of oral and parenteral methotrexate therapy in children with juvenile idiopathic arthritis: an observational study with patients from the German Methotrexate Registry. Arthritis Care & Research. 2012;64(9):1349-56.
2. Willot S NA, Deslandres C. Methotrexate in the treatment of inflammatory bowel disease: an 8-year retrospective study in a Canadian pediatric IBD center. Inflammatory Bowel Diseases. 2011;17(12):2521-6.
3. Uhlen S BR, Narebski K, Goulet O, Schmitz J, Cezard JP, Turck D, Ruemmele FM. Efficacy of methotrexate in pediatric Crohn's disease: a French multicenter study. Inflammatory Bowel Diseases. 2006;12(11):1053-7.
4. Deo M YA, Hill S et al. Methotrexate for treatment of atopic dermatitis in children and adolescents. International Journal of Dermatology. 2014;53:1037-41.
5. Rahman SI SE, Flanagan KH et al. The methotrexate polyglutamate assay supports the efficacy of methotrexate for severe inflammatory skin disease in children. J Am Acad Dermatology. 2014;70:252-6.
6. Roberts H OD. Methotrexate is a safe and effective treatment for paediatric discoid (nummular) eczema: a case series of 25 children. Australas J Dermatol. 2010;51:128-30.
7. Sidbury R DD, Cohen DE et al. American Academy of Dermatology. Guidelines of care for the management of atopic dermatitis Section 3. Management and treatment with phototherapy and systemic agents. J Am Acad Dermatol. 2014;71(2):327-49.
8. NICE. Guide to the methods of technology appraisal 2013 2013 [Available from: [https://www.nice.org.uk/article/pmg9/chapter/foreword.](https://www.nice.org.uk/article/pmg9/chapter/foreword)
9. Manca A, Hawkins N, Sculpher MJ. Estimating mean QALYs in trial-based cost-effectiveness analysis: the importance of controlling for baseline utility. Health Economics. 2005;14(5):487-96.
10. Ramsey SD, Willke RJ, Glick H, Reed SD, Augustovski F, Jonsson B, et al. Cost-Effectiveness Analysis Alongside Clinical Trials II—An ISPOR Good Research Practices Task Force Report. Value in Health. 2015;18(2):161-72.
11. Drummond MF, Sculpher MJ, Claxton K, Stoddart GL, Torrance GW. Methods for the Economic Evaluation of Health Care Programmes: Oxford University Press; 2015.
12. Husereau D, Drummond M, Petrou S, Carswell C, Moher D, Greenberg D, et al. Consolidated Health Economic Evaluation Reporting Standards (CHEERS)—Explanation and Elaboration: A Report of the ISPOR Health Economic Evaluation Publication Guidelines Good Reporting Practices Task Force. Value in Health. 2013;16(2):231-50.
13. Schram ME, Spuls PI, Leeflang MM, Lindeboom R, Bos JD, Schmitt J. EASI, (objective) SCORAD and POEM for atopic eczema: responsiveness and minimal clinically important difference. Allergy. 2012;67(1):99-106

1. Where available data within memory cells will be used as they are more normalised to potentially large differences between patients in the naïve vs memory compartment due to age. [↑](#footnote-ref-1)
